# A Genomic and Transcriptomic Overview of MATE, ABC, and MFS Transporters in *Citrus sinensis* Interaction with *Xanthomonas citri* subsp. *citri*

**DOI:** 10.3390/plants9060794

**Published:** 2020-06-25

**Authors:** Maria H. M. Julião, Saura R. Silva, Jesus A. Ferro, Alessandro M. Varani

**Affiliations:** Department of Technology, School of Agricultural and Veterinary Sciences, São Paulo State University, Jaboticabal 14884-900, Brazil; m.juliao@unesp.br (M.H.M.J.); saura.silva@unesp.br (S.R.S.); jesus.ferro@unesp.br (J.A.F.)

**Keywords:** citrus canker, comparative genomics, membrane transporters, differentially expressed genes, plant-pathogen interactions

## Abstract

The multi-antimicrobial extrusion (MATE), ATP-binding cassette (ABC), and major facilitator superfamily (MFS) are the main plant transporters families, playing an essential role in the membrane-trafficking network and plant-defense mechanism. The citrus canker type A (CC), is a devastating disease caused by *Xanthomonas citri* subsp. *citri* (Xac), affecting all citrus species. In this work, we performed an in silico analysis of genes and transcripts from MATE, ABC, and MFS families to infer the role of membrane transporters in Citrus-Xac interaction. Using as reference, the available *Citrus sinensis* genome and the citrus reference transcriptome from CitrusKB database, 67 MATE, 91 MFS, and 143 ABC genes and 82 MATE, 139 MFS, and 226 ABC transcripts were identified and classified into subfamilies. Duplications, alternative-splicing, and potentially non-transcribed transporters’ genes were revealed. Interestingly, MATE I and ABC G subfamilies appear differently regulated during Xac infection. Furthermore, *Citrus* spp. showing distinct levels of CC susceptibility exhibited different sets of transporters transcripts, supporting dissimilar molecular patterns of membrane transporters in Citrus-Xac interaction. According to our findings, 4 MATE, 10 ABC, and 3 MFS are potentially related to plant-defense mechanisms. Overall, this work provides an extensive analysis of MATE, ABC, and MFS transporters’ in Citrus-Xac interaction, bringing new insights on membrane transporters in plant-pathogen interactions.

## 1. Introduction

Plant-pathogen interactions result in macro and microscopic changes in the host plant, involving a wide range of morphological, biochemical, genetic, and molecular processes [1,2,3,4]. The defense-related processes triggered by plants during the interaction are fast and targeted to counter-attack the pathogen to maintain the cellular homeostasis [5]. One of the most studied and devastating plant diseases affecting all commercial citrus cultivars in production areas around the world is the citrus canker type A (CC), caused by the Gram-negative bacterium *Xanthomonas citri* subsp. *citri* (Xac) [6].

The Xac infects the *Citrus* spp. tissues through the penetration in stomatal pores or wounds made by thorns and insects [7]. The symptoms of infection start like high injuries soaked with water and evolve until it forms the cankers, reaching plant defoliation, and premature fall of fruits [7]. Moreover, distinct susceptibility levels to CC across *Citrus* spp. were observed [8], reflecting a complex array of plant-pathogen interaction and defense-response mechanisms [9].

Among the mechanisms involved in the defense responses, plants also developed and expanded transport mechanisms to excrete or sequester a broad range of synthesized molecules and xenobiotics to provide an efficient immune response [10,11]. A large number of primary and secondary active transporter genes found in plants improve the plant competition and adaptation to stress conditions [12], such as under the CC disease. The major transporters’ families multi-antimicrobial extrusion protein (MATE), ATP-binding cassette transporters (ABC), and major facilitator superfamily (MFS) play an essential role in the plants’ membrane-trafficking network [13]. They are ubiquitously present in prokaryotic and eukaryotes organisms, presenting distinct protein structures and sources of energy used to execute the transport of molecules [14,15].

Identifying and understanding the features and roles of membrane transporter proteins in plant species, such as the MATE, ABC, and MFS transporters in *Citrus* spp., has only been possible due to the growing number of genomic and transcriptomic data available in the public databases. Despite that, information concerning membrane transporters in *Citrus* spp. is still patchy. Thus, the importance of citriculture along with the constant threat to citrus cultivation and the posing substantial economic impacts of the CC disease prompted us to characterize and investigate the roles of these important membrane transporter families into the Citrus-Xac interaction.

As part of our efforts to understand the Citrus-Xac interaction, we built an in vivo database for the transcriptome of citrus and Xac interactome (CitrusKB database, http://bioinfo.deinfo.uepg.br/citrus/) [9]. The CitrusKB integrates gene expression data from eight *Citrus* spp. showing different CC susceptibility levels (e.g., from the less susceptible species: ‘Kumquat’ *Fortunella* spp., tangerine mandarin ‘Satsuma’ *C. unshiu*, and tangerine mandarin ‘Ponkan’ *C. reticulata*, to the intermediate susceptible as sweet oranges cultivars ‘Pera Rio’ and ‘Valencia’ *C. sinensis*, and highly susceptible as sweet oranges ‘Hamlin’ and ‘Bahia’ *C. sinensis*, and Acid lime ‘Galego’ *C. aurantifolia*) [9]. Furthermore, the CitrusKB provides the citrus reference transcriptome (CRT), composed of non-redundant sequences derived from the eight *Citrus* spp. available at CitrusKB. [9]. Using the CRT data from CitrusKB along with the public *C. sinensis* genome assembled at chromosome-scale (v2.0 HZAU), an in silico analysis was conducted with the aims to identify, classify, and propose potential roles for the MATE, ABC, and MFS gene families at genomic and transcriptomic contexts. Our results are not only providing novel insights about the main family of membrane transporters in *Citrus* spp. defense-related processes but also revealing a complex layer of the history of these gene families’ expansions and the transcriptional regulation under a plant-pathogen interaction.

## 2. Results

### 2.1. Identification of MATE, ABC and MFS Genes in the C. sinensis Genome

**Figure 1:**
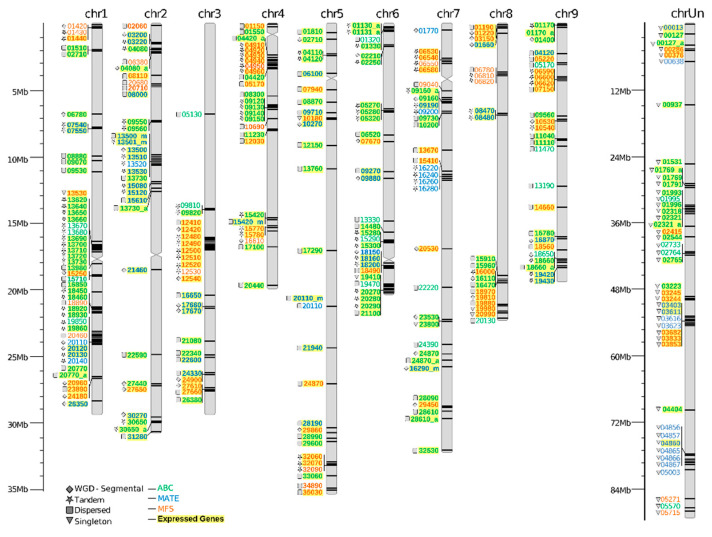
Genome distribution and gene duplication events of multi-antimicrobial extrusion protein (MATE), ATP-binding cassette transporters (ABC), and major facilitator superfamily (MFS) genes from *C. sinensis*. The size of a chromosome is indicated by its relative length given in Mb. Chromosome numbers (Chr) are indicated at the top of each chromosome. Gene duplication events are indicated with symbols. Genes with transcriptomic evidence from the citrus reference transcriptome under *Xanthomonas citri* subsp. *citri* (Xac) infection were shaded using yellow color and bold letters. The *C. sinensis* unplaced chromosome (chrUn) was not considered in the comparative genome analyses.

**Figure 2:**
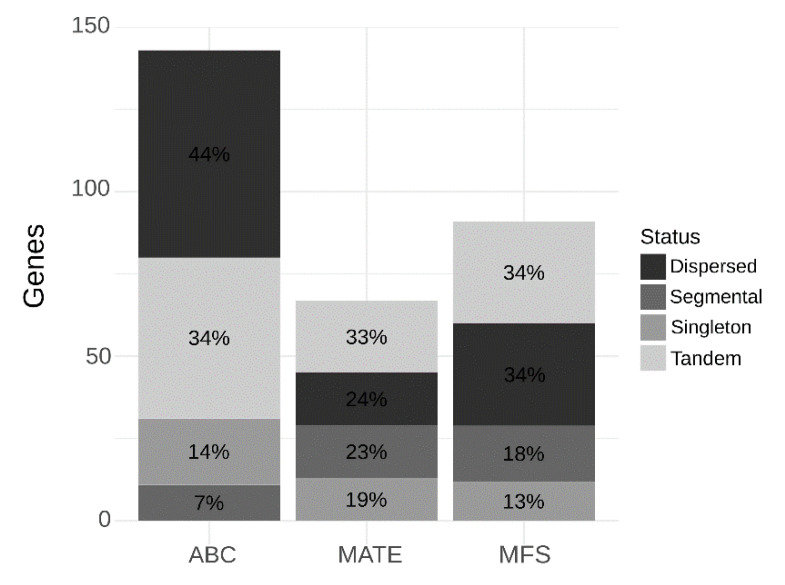
Duplication events of MATE, ABC, and MFS genes in the *C. sinensis* genome.

A total of 67 MATE, 143 ABC, and 91 MFS genes were identified in the *C. sinensis* genome (named hereafter as CsMATE, CsABC, and CsMFS, respectively). The genes synteny and their homologous chromosomal regions, corresponding to dispersed, tandem, proximal, and whole-genome/segmental duplication (WGD) events were mapped to *C. sinensis* chromosomes (2n = 18) showing an uneven distribution (Figure 1). Only assembled scaffolds representing the nine pseudochromosomes (Chr) were used in the following results. Most of the CsMATE, CsABC, and CsMFS genes were located in Chr 2, Chr 1, and Chr 4, respectively. Mostly CsMATE correspond to tandem duplicates, while the dispersed genes correspond to the majority of CsABC genes, and the CsMFS show the same distribution of tandem and dispersed genes (Figure 2). Interestingly, a smaller fraction of all membrane transporter genes was derived from WGD (Figure 2), suggesting their origin was related to the ancient γ triplication event shared by all core eudicots [16].

Moreover, we estimated the selection pressure acting on subfamilies from both MATE, ABC, and MFS families based on the dissimilarity level between the non-synonymous substitution (dN) and synonymous substitution (dS) values (Table S2). The average δ (dN − dS) and ω (dN/dS) value of subfamilies ranges from −34.90 to −7.87 and from 0.0819 to 0.299, indicating that a negative selection acted against extreme polymorphic variants. In particular, the subfamilies ABC C, MATE II, and inorganic phosphate transporter (PHT) appeared to be subject to a strong negative selection pressure.

### 2.2. Phylogenetic Analyses of MATE, ABC and MFS Genomic and Transcriptomic Sequences

**Figure 3:**
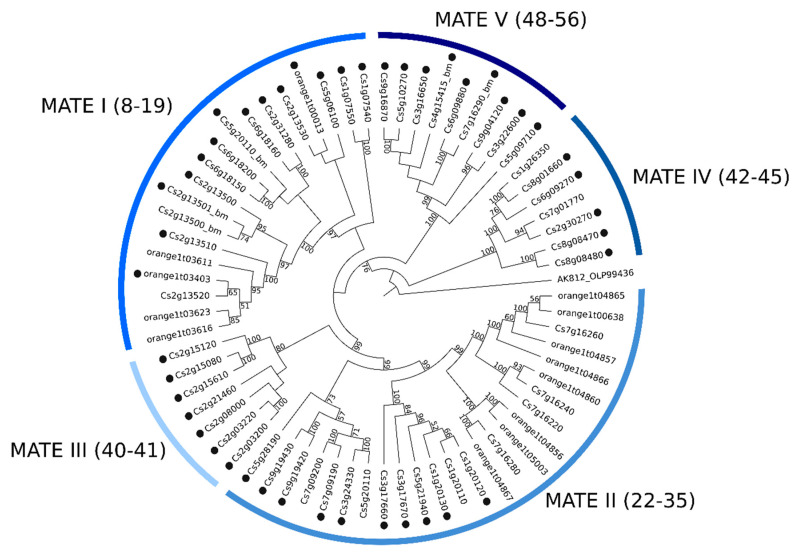
Maximum likelihood tree of 67 MATE amino acid sequences from *C. sinensis* genome. Numbers above the branches represent bootstrap values. The black circles represent genes that have an associated transcript sequence in the citrus reference transcriptome (CRT). AK812_OLP99436 was used as outgroup (Symbiodinium microadriaticum str. CCMP2467, Gene: slc47a1, multidrug, and toxin extrusion protein 1).

**Figure 4:**
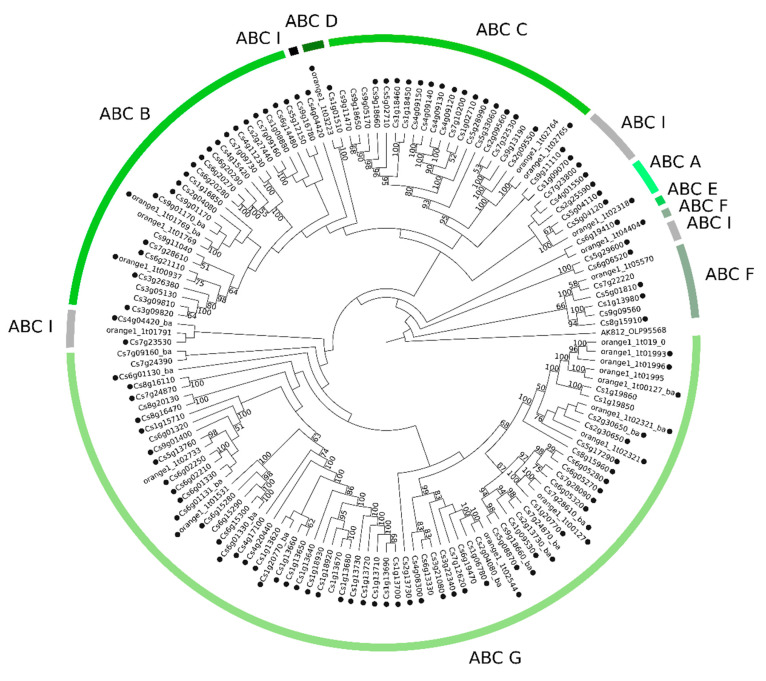
Maximum likelihood tree of 143 ABC amino acid sequences from *C. sinensis* genome. Numbers above the branches represent bootstrap values. The black circles represent genes that have an associated transcript sequence in the CRT. AK812_OLP95568 was used as outgroup (Symbiodinium microadriaticum str. CCMP2467, gene ABCF3, ATP-binding cassette subfamily F member 3).

**Figure 5:**
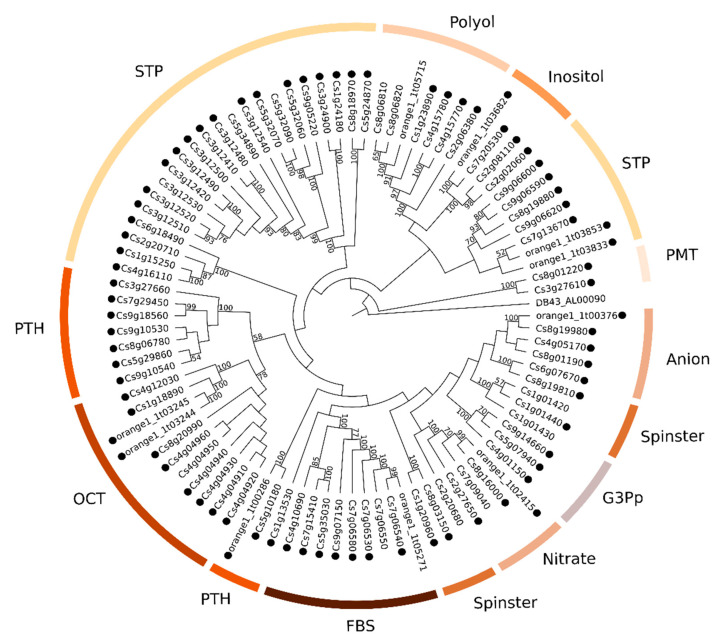
Maximum likelihood tree of 91 MFS amino acid sequences from *C. sinensis* genome. Numbers above the branches represent bootstrap values. The black circles represent genes that have an associated transcript sequence in the CRT. DB43_AL00090 was used as outgroup (Parachlamydia acanthamoebae, gene ywtG, putative metabolite transport protein YwtG).

The CRT’s re-annotation allowed us to identify a total of 82 MATE, 226 ABC, and 139 MFS transcripts (named hereafter as CstMATE, CstABC, and CstMFS, respectively). The maximum likelihood (ML) method was used to construct phylogenetic trees using datasets from genomic and transcriptomic sequences. The MATE genomic (Figure 3) and transcriptomic (Figure S1) phylogenetic trees revealed five clades designated as MATE I–MATE V. The ABC genomic (Figure 4) and transcriptomic (Figure S2) phylogenetic trees placed eight clades, distributed according to the eight known ABC subfamilies commonly found in plants (ABC A–ABC G and ABC I). The MFS family harbors 14 subfamilies, whose genomic (Figure 5) and transcriptomic (Figure S3) sequences were assigned into several clades (sugar transporters (STP), polyol, inositol, monosaccharide-sensing (PMT), anion, spinster, G3Pp, nitrate, folate-biopterin (FBS), PHT, and organic cation/carnitine (OCT)). The most abundant subfamilies identified in the *C. sinensis* genome and CRT corresponds to MATE I, ABC G, and STP subfamilies.

### 2.3. Global Characteristics of MATE, ABC and MFS Transcripts in Citrus spp.

Conserved transmembrane domains (TMD), nucleotide-binding domains (NDB), and motifs related to MATE, ABC, and MFS proteins (Table S1) were found in all selected and annotated transcripts sequences. The length of the CstMATE sequences varied from 92 to 611 aa, the predicted molecular weight (MW) and isoelectric point (pI) ranged from 10 to 65.5 kDa and, from 4.2 to 10.5, respectively. The length of the CstABC sequences varied from 114 to 1841 aa, and the predicted MW and pI ranged from 12 to 205 kDa and, from 4.3 to 11, respectively. The length of the CstMFS sequences varied from 87 to 747 aa, and the predicted MW and pI ranged from 9.6 to 80.1 kDa and, from 6.6 to 10.3, respectively. Both CstMATE, CstABC,, and CstMFS sequences were mostly located on the plasma membrane, except some ABC members (from subfamilies B, C, D, E, F, and I) located in the chloroplast (representing 10% of the total), and about 15% of both MATE (from subfamilies I, II, III, and V) and MFS (from subfamilies STP, PMT, and OCT) located in the vacuole (Table S1).

### 2.4. Transcriptome-Wide Identification of MATE, ABC and MFS Transcripts in Citrus spp.

At least 72% of CsMATE, 94% of CsABC, and 76% of CsMFS genes were transcribed under the Citrus-Xac interaction (Figure 1). Three CstABC transcripts showed no corresponding genes in the *C. sinensis* genome, even using tBlastn approaches. Since CitrusKB CRT was generated from eight *Citrus* spp. [9], this result may indicate that these transcripts are derived from other than *C sinensis*. Moreover, most transcripts were expressed by genes located on Chr 2, Chr 1, and Chr 4 for MATE, ABC, and MFS families, respectively (Figure 1, Table S4). Putative alternative splicing (AS) events were predicted in genes corresponding to multiples transcripts in more than 40% of both CsMATE (22 out of 48) and CsABC (51 out of 132), and CsMFS (28 out of 78) expressed genes (Table S4). In general, most genes that possibly underwent alternative splicing events produced two or three isoforms. Most isoforms identified belonged to ABC G (37%) and STP (26%) subfamilies, while in MATE, they were proportionally distributed between the subfamilies (Table S4). Moreover, the dispersed duplicates were the most predominant duplication events in the expressed genes from CsMATE, CsABC, and CsMFS families (Table S4).

### 2.5. MATE, ABC and MFS Gene Expression Analysis Based on CitrusKB Knowledge Base

**Figure 6:**
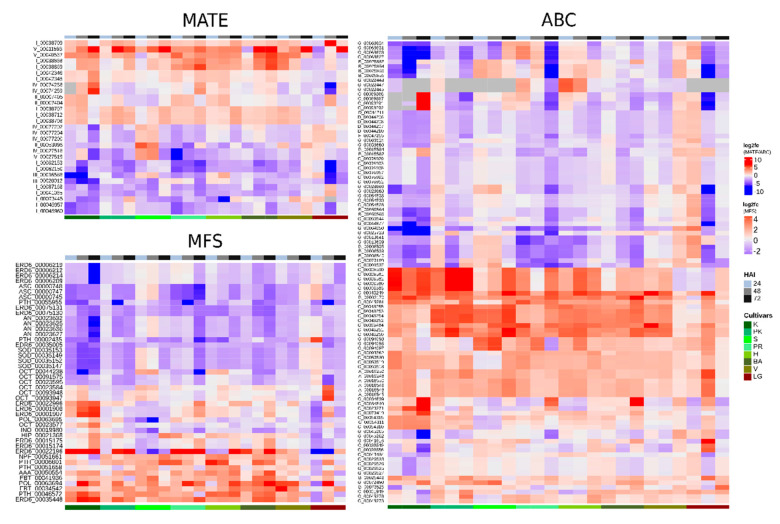
Heatmap of expression profiles of MATE, ABC, and MFS transcripts differentially expressed on at least one hours after bacterial inoculation (HAI) in a species. K: ‘Kumquat’, PK: ‘Ponkan’, S: ‘Satsuma’, PR: ‘Pera Rio’, H: ‘Hamlin’, BA: ‘Bahia’, V: ‘Valencia’, and LG: ‘Galego’. The identification of each transcript is given by the abbreviation of its respective subfamily followed by its identification number in the CRT (Figure S4 show the clusterized heatmap).

A total of 29 CstMATE, 100 CstABC, and 45 CstMFS transcripts were classified as differentially expressed (DE) in CitrusKB, whose some of them achieved six-fold expression after Xac inoculation (Figure 6, and in more details Figure S4). The patterns of gene expression after Xac infection are intricate (Figure 6), making it difficult to find precise results that may indicate some correlation of gene expression between the *Citrus* spp. in clustered heatmaps. Similarly, the membrane transporters seem to be under a complex global regulation in response to stresses or co-regulated with other genes [16,17], thus, endorsing our findings. Therefore, these results support a complex involvement of all transporters’ families in the array of plant-pathogen interaction and defense-response mechanisms of *Citrus* spp. against Xac.

The highest number of DE transcripts were from MATE I (23 DE), ABC G (63 DE), and STP (20 DE) subfamilies (Table S4, Figure 6). Among the up-regulated genes, the highest log_2_FC values were exhibited by MATE I and ABC C and G subfamilies, which can reflect a potential for high mRNA accumulation and protein production, supporting a role of the MATE I and ABC G subfamilies in the citrus response to Xac.

**Figure 7:**
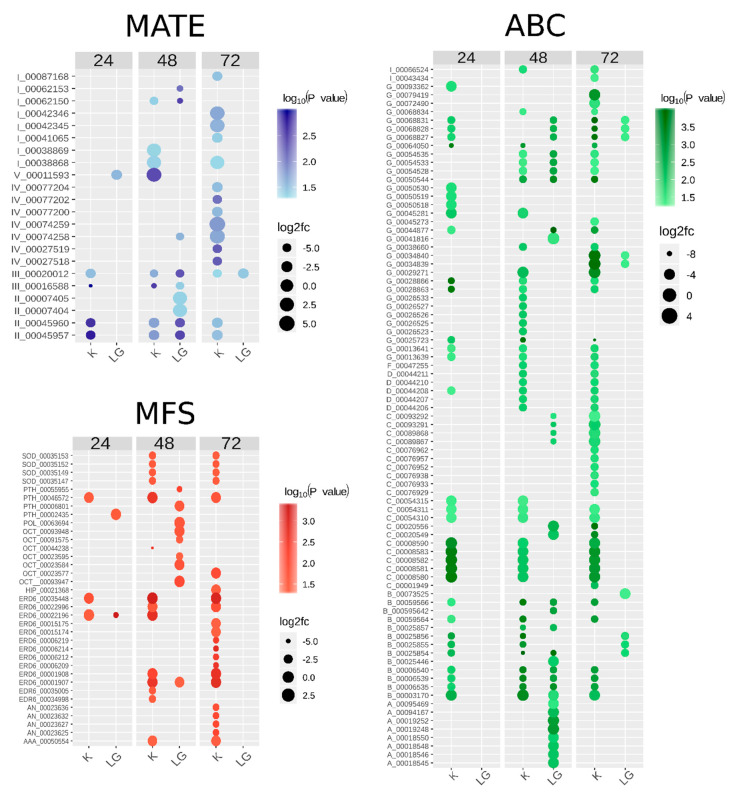
Bubble chart of MATE, ABC, and MFS transcripts differentially expressed in response to Xac infection in 24, 48, and 72 hours after bacterial inoculation. K: ‘Kumquat’, LG: Acid Lime ‘Galego’. The color of markers represents the −log_10_(*p*-value) values and the size is proportional to the log_2_FC values. The identification of each transcript is given by the abbreviation of its respective subfamily followed by its identification number in the CRT.

Furthermore, to better understand the regulation of membrane transporter genes in the Citrus-Xac interaction, we selected the most contrasting *Citrus* spp. to CC susceptibility level, ‘Kumquat’ (the less susceptible) and ‘Galego’ (the most susceptible) to analyze the DE transcripts. Interestingly, the species presented both different numbers of DE transcripts and different sets of transcripts in each hours after bacterial inoculation (HAI) (Figure 7), which can reflect their different levels of CC susceptibility and the molecular mechanisms of plant defense against Xac. Most transcripts were DE in all HAI in ’Kumquat’, while in ’Galego’ they did not show differential expression or showed only after 72 HAI. Similarly, no DE transcript from ABC family was observed at 24 HAI (Figure 7), which can be related to a late activation of the primary transporter genes in the highly susceptible species to CC.

## 3. Discussion

### 3.1. Identification of MATE, ABC and MFS Genes in the C. sinensis Genome

We identified 67 MATE, 143 ABC, and 91 MFS genes in the *C. sinensis* genome. A variable number of membrane transporter genes are found in plant genomes species, whose differences in the gene family size across species may be a result of lineage-specific expansions [18]. That diversity may imply in the expansion and diversification of membrane transporter families since ABC transporters and secondary active transporters (including MATE and MFS) are primarily found in plants from distinct environments [12]. Indeed, the plants require different sets of transporters to maintain primary metabolism under changing conditions [12].

The CsMATE, CsABC, and CsMFS genes were unevenly distributed into the *C. sinensis* nine chromosomes (Figure 1). This result is consistent with previous reports on the distribution of MATE and ABC genes in plant species [19,20,21]. The Chr 2 harbors about 24% of the CsMATE genes, likewise, 19% of the MATE gene family in *Gossypium arboreum* was located in Crh 10, and 17% in Chr 12 of *G. raimondii* genomes [22]. In contrast, approximately 18% of CsMFS genes were located in Chr 9. The highest numbers of CsABC genes were found on Chr 1 (about 17% of the annotated genes), similarly to the 20% of rice ABC genes in Chr 1, while 15% of *Solanaceae* ABC genes were located in Chr 12, 30% of *Arabidopsis thaliana* ABC genes in Chr 3, and 26% of grape ABC genes in Chr 9 [19].

We conducted an in silico gene classification to infer the homologous chromosomal regions of CsMATE, CsABC, and CsMFS genes. Approximately 21% of both CsMATE and CsABC genes and 10% of CsMFS were classified as singletons (Figure 2). Gene duplication is a source of genetic novelty, acting in gene family expansion and protein functional diversification in large gene families [23]. Distinct groups of transporter genes were expanded during the evolution of plants, multiplied to enable many aspects of a sessile plants’ lifestyle [12] by sending information between cells and exchanging substances and toxic compounds [11]. For instance, the larger ABC subfamilies (B, C, and G) were enriched by gene duplications, as previously found in six plant genomes (*Oryza sativa*, *Solanum lycopersicum*, *S. tuberosum*, *A. thaliana*, *Vitis vinifera*, and *Volvox carteri*) and the yeast *Saccharomyces cerevisiae* [19].

The tandem duplicates were the most abundant duplication event in CsMATE genes, comprising 37% (Figure 2). Tandem events constituted about 36% of MATE gene duplications occurred in *Arabidopsis* [24], 21% in soybean [20], 20% in rice [24], and 55% in tomato [25], indicating a considerable implication of this evolutionary mechanism in the expansion of the MATE family and suggesting for a similar expansion process in *C. sinensis*. In contrast to MATE, the most common duplication events of CsABC and CsMFS genes originate from dispersed duplication (Figure 2). Tandem and dispersed duplicates may arise by unequal crossing over between similar alleles, and translocations originated by transposable elements (TEs), respectively [18]. Taken together, these findings strongly suggest for genomic evolution in the opposite direction for the membrane transporters’ families (i.e., unequal crossing over for CsMATE, and translocations originated by TEs for CsABC and CsMFS). Moreover, the duplication events may have also contributed to the expansion and functional redundancy, and sub-functionalization of membrane transporter families in *C. sinensis*.

### 3.2. Transcriptome-Wide Identification of MATE, ABC and MFS Transcripts in Citrus spp.

A total of 82 MATE, 226 ABC, and 139 MFS transporters were identified in the citrus reference transcriptome. The amino acid sequences length varied from 89 to 611 aa in CstMATE sequences, from 77 to 1841 aa in CstABC sequences, and from 87 to 747 aa in CstMFS sequences. More than 60% of CstMATE, CstABC, and CstMFS sequences showed pI values higher than 7.0 (Table S3), indicating an overall global primary character. A variation in physicochemical and molecular properties like sequence length, molecular weight and, pI was previously reported in MATE and ABC sequences from monocot and dicot plant species [19,24]. Furthermore, the subcellular localization analyses revealed that the plasma membrane as the central cell membrane system identified in the three families (Table S3). Indeed, the two primary sites of plant MATE transporters are either the plasma membranes or the vacuolar membranes [26]. However, the localization of *Citrus* spp. membrane transporters requires further functional validation.

The majority of MATE, ABC, and MFS transcripts correspond to genes with transcriptional evidence associated with CRT. However, some genes were supposed not to be transcribed (Figure 1). The putative non-expression of some genes can reflect in an energy-saving action underlying specific environmental conditions or a tissue-specific expression since a tissue-specific expression was reported for some MATE and ABC genes in maize [27,28]. Thus, it is tempting to speculate that the set of non-expressed genes may be nonessential in the tissue used to assemble the CRT from CitrusKB (leaves) and/or to Citrus-Xac interaction.

Moreover, we verified some examples of more than one citrus transcript from MATE, ABC, and MFS families associated with the same gene identification from the *C. sinensis* genome (Tables S2 and S3). This finding may indicate alternative splicing events occurring during the transcription. Stress conditions frequently induce alternative splicing events in plants [29]. In biotic stresses, the primary AS-based regulation mechanism in plant responses is the rearrangement of exons and modular domains, representing a source of transcriptome and proteome diversity, influencing the number of functional proteins produced quantitatively regulating the gene expression [30]. However, further studies are required to determine which of the citrus AS events are biologically relevant during the Citrus-Xac interaction.

### 3.3. Phylogenetic Based Classification and Gene Expression Analyses

Phylogenetic analyses were conducted for MATE, ABC, and MFS using genes (Figure 3, Figure 4 and Figure 5) and transcripts sequences (Figures S1–S3). The transporter’s functions and substrate specificity typically correlate with clades, and together with computational annotation analyses provide a satisfactory base for making functional and evolutionary predictions [21,28,31,32,33]. For instance, our results supported for 60% on average of amino acid conservation between *Citrus* spp. and *A. thaliana* MATE, ABC, and MFS proteins. Moreover, the gene expression analysis using the CRT data contributed to shedding light on the involvement of the membrane transporters members in the Citrus-Xac interaction. The MATE, ABC, and MFS candidate genes, highlighted as potential targets for further biotechnological studies, are discussed in the following sections.

#### 3.3.1. Phylogenetics and Gene Expression Analyses of MATE Sequences

The five main clades were shown in the MATE phylogenetic trees (Figure 3, Figure S1), designated as subfamilies MATE I–V. In soybean and potato, six MATE subfamilies were identified according to the previous phylogenetic studies. In contrast, four subfamilies were identified in rice, *Arabidopsis*, and tomato, and three in cotton [24,34,35], suggesting a diversification among plant species and a high performance of membrane transporters in the complex plant processes.

The MATE I subfamily contains 19 genomic sequences from C. sinensis and a total of 26 transcripts annotated as detoxification proteins (DTX, as the classification of *A. thaliana* MATE members; [36]) 8–19 from the CRT (Table S3). Considering the contrasting species, ‘Kumquat’ (K) and acid lime ‘Galego’ (LG), the two isoforms from Cs1g07540 (annotated as DTX19) were highly DE at 72 HAI in ‘Kumquat’. A direct role in either the vacuolar sequestration or the cellular efflux of toxins was proposed to AtDTX19. Moreover, the ability to sequester the toxic cation tetramethylammonium expressed and required in different tissues [37], can explain the plasma membrane and endomembrane system locations predicted for the eight CstDTX19. Then, the CstDTX19 transcripts can regulate the sequestration and, consequently, the tolerance towards xenobiotics after three days in Xac infection, which likely provides efficient adaptive stress responses to the CC disease.

The MATE II subfamily contains 25 genomic sequences from *C. sinensis* and a total of 22 transcripts annotated as (DTX 22–35) from the CRT (Table S3). Of these, eight CstDTX27 transcripts share at least 70% of similarity with OsMATE1, reported to negatively regulate disease tolerance apart from governing growth and development in maize [38]. The variation in gene expression profiles among different *Citrus* spp. observed in the MATE II transcripts (Figure 6) can potentially facilitate a stress-related response. Then, we suggest an involvement of CstDTX27 transcripts in disease tolerance regulation, which were shown to be promising candidates for further exploitation.

Moreover, secondary metabolites transporters from diverse plant species share 68% to 83% of sequence similarity with both CstDTX35 sequences. For instance, VvAM1 and VvAM3 (anthocyanin-acyl glucosides uptake) [39], MtMATE2 (transport of glycosylated flavonoids) [40], AtDTX35 (flavonoid levels and metabolism changes) [41], and SlMTP77 (transport of anthocyanins into the vacuole) [42]. These findings suggest a potential role for MATE II members related to anthocyanin sequestration in vegetative tissues.

Changings in secondary metabolite levels during plant-pathogen interaction has been observed in *Citrus* spp. [43,44]. For instance, in leaves from ‘Hamlin’ and ‘Valencia’ sweet oranges, the flavonoid glycosides, polymethoxylated flavones, and hydroxycinnamates compounds levels increased more than 10-fold after Candidatus Liberibacter infection [44]. Similarly, MATE II members showed differential expression after Xac infection (Figure 6), representing a great involvement of the secondary metabolites’ secretory pathway in citrus defense response to Xac infection.

The MATE III, IV, and V subfamilies seem to be involved in metal tolerance. Some transcripts from the CRT and the AtDTX46 are localized at the chloroplast compartment, which is proposed to most likely transports related substances such as phenolic acids [45]. The gene expression of transcripts from both families was mostly negatively regulated after Xac infection in the less and intermediate susceptible *Citrus* spp. (‘Kumquat’, ‘Ponkan’, and ‘Pera Rio’). This finding may indicate that metal detoxification and tolerance tests can be performed to better conclusions in Citrus-Xac impact. Citrus plants could secrete phenolics by MATE III and IV transporters to take up apoplasmic precipitated iron, acting in metal detoxification and tolerance due to its close relation with characterized non-citrate transporters. Likewise, the CstDTX48 and CstDTX49 transcripts share the most significant alignments with MtMATE55 and AtDTX48 (74% to 86% of Sequence Similarity and 92% to 97% of Sequence Coverage), transporters involved in iron homeostasis [46,47]. The gene expression values of CstDTX48 after bacterial infection were high and increasing, especially in 72 HAI, reaching more than 128-fold times in expression, suggesting a great involvement of this member in cellular homeostasis during stress responses.

#### 3.3.2. Phylogenetics and Gene Expression Analyses of ABC Sequences

Based on the phylogenetic relationships, eight ABC subfamilies were identified (ABC A–ABC G, except ABC H; Figure 4, Figure S2), a conserved structure in plant species [19]. We identified three genomic sequences from ABC A subfamily in the *C. sinensis* genome. All of them have at least one transcript associated with the CRT (Figure 4), totaling eight transcripts from the CRT (Table S3). Members belonging to subfamily ABC A were reported to be involved in salt stress and lipid metabolism by mediating the transport of fatty acids to the endoplasmic reticulum [48]. The fatty acid metabolism represents a point of convergence and modulation of crosstalk between diverse defense signaling cascades involving salicylic and jasmonic acids [49]. Eight CstABC A members were identified in *Citrus* spp., whose six members exhibited a significant impact in their gene expression after Xac infection, which were up-regulated at 48 and 72HAI (Figure 6, Table S3). The transport of the fatty acids in *Citrus* spp. seems to be mainly positively regulated before two days of infection, influencing the metabolic enzymes involved in fatty acid and signal molecules during the defense response to Xac.

A total of 26 and 21 genomic sequences from ABC B and ABC C subfamilies, respectively, were identified in the *C. sinensis* genome, of which 40 ABC B and 40 ABC C transcripts were identified in the CRT (Table S3), which could indicate for a potential role of these subfamilies in the *Citrus* spp. defense responses. Both subfamilies members are involved with the transport of plant metabolites. The ABC B subfamily is reported to be involved in auxin transport, translocation of xenobiotics, and alkaloids [50,51]. In contrast, ABC C members are involved in the transport of glutathione conjugates and vacuolar transporter of anthocyanins [52,53]. Nine ABC B transcripts were DE at 24 HAI in ‘Kumquat’, while no transcript in ‘Galego’, suggesting a more efficient response in the less susceptible species (Figure 7). Patterns of high gene expression were identified in ABC C transcripts, especially in the less susceptible *Citrus* spp. (Figure 6 and Figure 7), which points to a more powerful generation of anthocyanins and conjugated metabolites to control/cease the bacterial infection.

The wide range of putative transporters of plant metabolites, differentially expressed after Xac infection, can represent a substantial effect of Xac mechanisms on the metabolite composition of the citrus plants. This finding may also support a large number of ABC B, ABC C, and MATE II transporters expressed by *Citrus* spp. under Xac infection, suggesting a contribution with the secreted compounds to pathogen defense at the leaf surface of citrus plants. Thus, considering the gene expression pattern of these families in *Citrus* spp., and the fact that different transporter families may act mutually to transport a particular metabolite [13,15], it is possible to speculate for a cooperative transport mediated by the ABC B, ABC C, and MATE II members for the extrusion of defense-related molecules in *Citrus* spp.

The ABC subfamilies comprising the smallest numbers of members in the *Citrus sinensis* genome were ABC D, ABC E, and ABC F (two, one, and eight, respectively; Figure 4). A peroxisomal import of substrates for β-oxidation and the glyoxylate cycle, jasmonic acid precursors, and/or its acyl-CoA derivative into the peroxisome has been suggested for the ABC D subfamily [54]. The DE transcripts were expressed by two of the less susceptible species (e.g., ‘Ponkan’ and ‘Kumquat’). No regulation was observed in the highly susceptible species ‘Galego’ (Table S5, Figure 7). This suggests a role of ABC D members in lipid metabolism and signaling in the susceptible citrus plants to CC in response to Xac infection. Still, the ABC E subfamily was found to be the smallest amongst the ABC subfamilies in *A. thaliana*, rice, maize, grape, and tomato. It was represented in most genomes by a single-copy gene [32,33,55,56]. The ABC E members are involved in ribosome biogenesis and recycling in eukaryotes; however, the mutant phenotypes in plants remain to be explained [57].

Nevertheless, no DE transcript belonging to subfamily E was found, suggesting no direct function of ABC E subfamily in Citrus-Xac interaction. The seven ABC F members identified in the CRT, on the other hand, exhibited negative log_2_FC values, and only TCONS_00047255 were down-regulated (Table S5). The LrABCF1 gene from *Lilium regale* functions as a positive regulator of plant defense against cucumber mosaic virus, tobacco rattle virus, and *Botrytis cinerea* in petunia [58]. Then, ABC F members might be involved in stress-associated control during Citrus-Xac interaction, acting as a late negative regulator of plant defense.

Furthermore, we also identified 72 genomic sequences from ABC G subfamily in the *C. sinensis* genome, of which 57 of them have at least one transcript associated in the CRT (Figure 4), and a total of 102 transcripts in the CRT (Table S3), representing more than 40% of annotated CstABC transporters. A close rate was observed in *A. thaliana*, *O. sativa*, *S. lycopersicum*, *S. tuberosum*, and *V. vinifera* genomes [19]. In addition to the most significant number of members, the subfamily G harbors the largest number of DE transcripts and the genes that most underwent AS events, reflecting family diversification. Its outstanding diversification is thought to be linked to the requirement of a reliable system to address molecules as nutrients or secondary metabolites to the appropriate cellular compartments for adaptation and survival of plants on the land environment [12]. Proteins belonging to the ABC G subfamily are involved in response to biotic stress and contribute to the transport of signaling molecules or secretion defense-related metabolites and a wide diversity of substrates [59,60]. For instance, the mRNA level of AtABCG36 is elevated during plant infection with virulent and avirulent strains of the bacterial pathogen *Pseudomonas syringae* [61] as observed in the less susceptible species (‘Kumquat’), which up-regulated a higher number of ABC G transcripts than the highly susceptible species (‘Galego’) (Figure 7).

It was also shown that ABCG32 in *Arabidopsis* and HvABCG31 in barley are required for the formation of a functional cuticle, acting as the first barrier against abiotic and biotic stresses [62,63]. Still comparing the contrasting species, the ABCG32 related transcripts from CRT were up-regulated at all HAI in ‘Kumquat’ and not deferentially expressed in ‘Galego’ whereas ABCG31 transcripts were up-regulated only at 72 HAI in both species (Figure 7). Since the expression of ABC G proteins in plants is predominately in leaves and roots [64], the expression patterns of ABC subfamily G members during Citrus-Xac interaction might indicate a role in the extrusion of defensive compounds and barriers development in citrus leaves. In ultimate, it may minimize stress-induced damage and counteracting the pathogen.

#### 3.3.3. Phylogenetics and Gene Expression Analyses of MFS Sequences

The major facilitator superfamily harbors the highest number of subfamilies (14) in *C. sinensis*, compared to the MATE and ABC families. The number of genomic sequences varied from 2 to 23 in *C. sinensis* and from 3 to 38 transcriptomic sequences from the CRT (Figure 6 and Figure S3). The largest subfamily, the STP, comprises 23 genes, while 38 transcripts were expressed in the CRT. The STP family is also the more abundant MFS subfamily in *A. thaliana*, which includes 106 out of 218 members [65], mediating the uptake of a wide range of sugar substrates which are adjusted to the type of tissue, developmental stage, metabolic state, and environmental conditions [66]. Some STP members were characterized to be involved in biotic stresses, such as STP3 and STP4, involved in strikingly distinct induction kinetics by catalyzing monosaccharide import into classic sinks [67]. Moreover, leaf STP8 is involved in importing sugars for component systems of secondary products and degradative enzymes important in plant-insect interactions [66]. The DE transcripts from the STP family were highly recruited after Xac infection, especially by the less susceptible species to CC and in 48 and 72 HAI (Table S5, Figure 7), suggesting a role in depriving the apoplastically growing pathogens by limiting their sugar source.

Curiously, the MFS members also act in the inorganic phosphate (Pi) transport activities in the plant, which belong to the inorganic phosphate transporter subfamily (PHT). The PHT proteins in *A. thaliana* are involved in the transport of Pi between the cytosol and chloroplasts, heterotrophic plastids, and the golgi apparatus [68]. We identified seven genomic sequences in the *C. sinensis* genome and ten transcripts expressed in the CRT, of whose four was DE (Table S5). In the less susceptible species to CC, ‘Kumquat’, the transcript TCONS_00046572 was up-regulated after Xac infection in all the HAI while in the highly susceptible ‘Galego’, three other transcripts were regulated 24 and 48 HAI (Figure 7). Then, the PHT members may be involved in the plants complex network of maintenance of Pi homeostasis through regulation of the abundance of PHTs in the citrus leaves during the defense responses to Xac.

On the other hand, the smallest subfamilies (Spinster, PMT, Nitrate and Peptide, Inositol, Ascorbate, and Anion) are probably responsible for the transport of anions, inositols, sphingolipids, and lipids, including six or fewer genes in the *C. sinensis* genome. Furthermore, the available information on these MFS subfamily members is still poorly investigated or not yet addressed experimentally. Despite that, we were able to infer CsMFS members’ potential in meeting the carbohydrate demand of cells responding to bacterial stress, providing an efficient adaptive fit to the CC disease.

In conclusion, we hypothesize a complex strategy adopted by citrus plant cells for regulation of multiple members from MATE II, ABC B, ABC C, ABC G, and STP subfamilies as the source of proper defense responses to Xac infection. According to our findings, some membrane transporters members are highlighting as potential targets for functional studies (Table 1, Figure S5).

## 4. Materials and Methods

### 4.1. Identification of MATE, ABC and MFS Genes in the C. sinensis Genome

The latest version of *Citrus sinensis* genomic data (‘Valencia’ version 2.0) was retrieved (https://www.citrusgenomedb.org/analysis/186). The MATE, ABC, and MFS transporters genes from *C. sinensis* (CsMATE, CsABC, and CsMFS, respectively) were retrieved by keyword search using the *Arabidopsis thaliana* UniProt [69] nomenclature for MATE [35,36], ABC [70], and MFS [65] against the annotation files. We conducted two additional searches: (a) a tblastN using the sequences previously identified in the first search as query against the *C. sinensis* amino acid sequences and (b) a blastp using *Arabidopsis thaliana* MATE, ABC, and MFS amino acid sequences from UniProt database [69]. The presence of MATE, ABC, and MFS-related protein domains (Table S1) in the potential *C. sinensis* genes identified were manually verified using InterProScan [71] results.

The homologous chromosomal regions events that occurred in *C. sinensis* were inferred using the Duplicate Gene Classifier from MCXScan toolkit [72]. We considered the anchor genes in collinear blocks like WGD. Paralogs far each other on chromosomes without conserved synteny like dispersed duplicates. Two gene copies closely located on the chromosome but separated by up ten genes like tandem genes. The genes distribution and duplication events were mapped to *C. sinensis* nine pseudomolecules (and one additional, the unplaced pseudomolecule).

Moreover, the selective pressure in the ABC, MATE, and MFS genes were investigated by determining the non-synonymous and synonymous nucleotide substitution, indicated as δ, using the Nei–Gojobori method [73], with *p*-value <0.05. For that, all the coding genes sequences were previously aligned using MUSCLE-Codon alignment [74] with default parameters. All positions with less than 80% site coverage were eliminated. To test for a variance, we performed the bootstrap method with 500 replicates. Evolutionary analyses were conducted in MEGA10 [75]. In addition, we used the SLAC algorithm [76] implemented in the Datamonkey server [77] to search the proportion of sites under selection (ω).

### 4.2. Transcriptome-Wide Identification of MATE, ABC and MFS Transcripts in Citrus spp.

The citrus reference transcriptome (retrieved from CitrusKB, [9]) was re-annotated with Blast2GO [78], using UniProt Viridiplantae database [68], and InterProScan [71]. Searches to select potential MATE, ABC, and MFS sequences expressed by *Citrus* spp. were conducted in the same way as genomic sequence searches. The sequence length, isoelectric point value, and molecular weight of MATE, ABC, and MFS amino acid sequences were predicted by EMBOSS-PepStat [79]. The subcellular location was predicted by WoLF PSORT [80].

Each CstMATE, CstABC, and CstMFS transcript was mapped in the *C. sinensis* genome using the tBlastN tool to identify their corresponding genes. To find all the possible transcripts generated by membrane transporter genes and to propose the occurrence of potential alternative splicing events and isoforms generation, the alignments were investigated manually (using a minimum of 90% identity, minimal alignment length of 100 amino acids, and expect the value of 1e-5).

### 4.3. Phylogenetic Analyses of MATE, ABC and MFS Genomic and Transcriptomic Sequences

MATE, ABC, MFS, and outgroup amino acid sequences from proteins predicted from the *C. sinensis* genome were aligned using the multiple sequence aligner MAFFT version 7.4 [81], with standard parameters. The outgroup sequences AK812_OLP99436, AK812_OLP95568, and DB43_AL00090, were retrieved from HMMER Ensembl Genomes. The best-of-fit model LG+G+F [82] was estimated for both ABC and MFS datasets, while WAG+G+F [83] for the MATE dataset (PhyML 3.0, [84]). Similarly, the MATE, ABC, and MFS amino acid sequences from the CRT were aligned using MAFFT, with standard parameters. The best phylogenetic model LG+G+F [82] was estimated for both MATE and ABC datasets, while VT+G+F [85] was best for the MFS dataset (PhyML 3.0, [84]). All evolutionary models were performed considering the Akaike information criteria.

Phylogenetic analyses were conducted in the Portal CIPRES v. 3.3 [86]. The RaxML-HPC2 [87] tool was chosen for the maximum likelihood approach. The branches’ support of the ML trees was evaluated by rapid bootstrap [84] with 1000 replicates. The branch support values are displayed in each clade of the phylogenetic trees. Clades without values correspond to the absence of bootstrap support.

### 4.4. MATE, ABC and MFS Gene Expression Analysis Based on CitrusKB Knowledge Base

Gene expression data from *C. reticulata*, *C. unshiu*, *C. sinensis* (‘Pera Rio’, ‘Valencia’, ‘Hamlin’, and ‘Bahia’ cultivars), *C. aurantifolia*, and *Fortunella* spp. were obtained from CitrusKB [9]. To filter the DE transcripts that probably mostly impacted the plant defense responses under Citrus-Xac infection we used a threshold of *p*-value <0.05 and log_2_fold-change (log_2_FC) values higher than 1 (up-regulated) or lower than −1 (down-regulated) (DESeq package [88]). The −log_10_(*p*-value) and log_2_FC values of the DE transcripts were plotted in a volcano plot using the ggplot2 package in R [89]. Heatmaps of DEs expression profiles were constructed using the ComplexHeatmap package in R [90]. Besides, the expression and significance values of DE transcripts from the less and the highly susceptible species to CC (‘Kumquat’ and ‘Galego’, respectively) were plotted using the ggplot2 package in R [89].

## 5. Conclusions

MATE, ABC, and MFS transporters are essential proteins under plant-pathogen interactions due to their role in the excretion or uptake of macronutrients, defense-related compounds, xenobiotics, and metabolic products. Five MATE, eight ABC, and fourteen MFS subfamilies were identified in *C. sinensis*. Among those, we identified four MATE, ten ABC, and three MFS members whose putative roles during the Citrus-Xac interaction revealed potential targets for functional studies. Moreover, the subfamilies MATE I and ABC G are key players in managing the compounds produced by plants during the defense responses. The transcriptional evidence of both membrane transporter family members based on CRT data supports the hypothesis for a highly complex gene expression regulation during the Citrus-Xac interaction, which reflects the intricate defense-related processes in CC susceptibility levels between *Citrus* spp. Furthermore, alternative splicing and gene duplication events may represent evolutionary strategies to increase the membrane transporters’ numbers and functional diversification in *Citrus* spp. for proper stress responses to the CC disease. Altogether our findings offer useful information, highlighting membrane transporters for potential biotechnological applications. In addition, these results may also represent a basis for the functional characterization of MATE, ABC, and MFS transporters in *Citrus* spp., helping to address several biological questions concerning plant membrane transporters families.

## Figures and Tables

**Table 1:** Promising candidates for further exploitation based on their potential role in the Citrus-Xac interaction.

Gene	Acession Number	Subfamily in *C. sinensis*	Closest Homolog	*A. thaliana* Homolog	Potential Role in the Citrus-Xac Interaction
Cs1g07540	XP_006464779.1	MATE I	*Herrania umbratica* DTX18	At3g23560	Regulation of the tolerance towards xenobiotics
Cs2g13530	XP_006468796.1	MATE I	*Pistacia vera* DTX16	At5g52450	
Cs1g20130	XP_006466279.1	MATE II	*Quercus lobata* DTX27	At5g65380	Disease tolerance regulation
Cs7g09190	XP_006483932.1	MATE II	*Pistacia vera* DTX29	At3g26590	
Cs3g26380	XP_006473687.1	ABC B	*Pistacia vera* ABC B13	At1g27940	Transport of secondary metabolites (antocyanin, flavonoids, alkaloids, etc.)
Cs6g20270	XP_006482502.1	ABC B	*Pistacia vera* ABC B15	At3g28345	
Cs7g10200	XP_006484035.1	ABC C	*Pistacia vera* ABC C15	At3g13080	
Cs1g18450	KDO65310.1	ABC C	*Populus alba* ABC C8	At3g21250	
orange1.1t02765	XP_006493359.1	ABC C	*Pistacia vera* ABC C13	At2g07680	
orange1.1t02321	XP_006492936.1	ABC G	*Hevea brasiliensis* ABC G15	At3g21090	Transport of signaling molecules, secretion of defensive compounds, and barriers development in citrus leaves
Cs5g17290	XP_006478168.1	ABC G	*Gossypium raimondii* ABC G11	At1g17840	
Cs8g16470	XP_024958158.1	ABC G	*Pistacia vera* ABC G31	At2g29940	
Cs9g01400	XP_015388954.1	ABC G	*Populus trichocarpa* ABC G37	At3g53480	
Cs4g17100	XP_006475761.1	ABC G	*Pistacia vera* ABC G32	At2g26910	
Cs3g24900	XP_006473508.3	STP	*Pyrus x bretschneideri* ERD6-like 6	At1g75220	Limit a sugar source of *Xanthomonas citri* subsp. *citri*
Cs1g24180	KDO79562.1	STP	*Durio zibethinus* ERD6-like 6	At1g69650	
Cs5g32060	XP_006479807.1	STP	*Pistacia vera* ERD6-like 7	At2g45820	

## Supplementary Materials

The following are available online at https://www.mdpi.com/2223-7747/9/6/794/s1, Figure S1: Maximum likelihood tree of MATE amino acid sequences expressed by *Citrus* spp. identified in the citrus reference transcriptome. Numbers above the branches represent bootstrap values. AK812_OLP99436 was used as outgroup (*Symbiodinium microadriaticum str*. *CCMP2467*, Gene: slc47a1, multidrug, and toxin extrusion protein 1), Figure S2: Maximum likelihood tree of ABC amino acid sequences expressed by *Citrus* spp. identified in the citrus reference transcriptome. Numbers above the branches represent bootstrap values. DB43_AL00090 was used as outgroup (*Parachlamydia acanthamoebae*, gene ywtG, putative metabolite transport protein YwtG), Figure S3: Maximum likelihood tree of MFS amino acid sequences expressed by *Citrus* spp. identified in the citrus reference transcriptome. Numbers above the branches represent bootstrap values. DB43_AL00090 was used as outgroup. (*Parachlamydia acanthamoebae*, gene ywtG, putative metabolite transport protein YwtG), Figure S4: Clustered heatmaps of MATE, ABC, and MFS differentially expressed transcripts on at least one HAI in a species. K: ‘Kumquat’, PK: ‘Ponkan’, S: ‘Satsuma’, PR: ‘Pera Rio’, H: ‘Hamlin’, BA: ‘Bahia’, V: ‘Valencia’, LG: ‘Galego’, and HAI: Hours after bacterial inoculation. The identification of each transcript is given by the abbreviation of its respective subfamily followed by its identification number in the CRT, Figure S5: Volcano plot of MATE, ABC, and MFS differentially expressed transcripts. The 50 differentially expressed transcripts (Derived from the 4 MATE, 10 ABC, and 3 MFS that are potentially related to plant-defense mechanisms) associated with the plant membrane transporters gene candidates who most significantly impacted the plant defense responses under Citrus-Xac infection are annotated in the plot, Table S1: Domains and motifs related to MATE, ABC, and MFS proteins used in the proteins manually verification, Table S2: Estimation of non-synonymous and synonymous substitutions mean dissimilarity for each sub-family (δ = d_N_ − d_S_ and ω = d_N_/d_S_), Table S3: MATE, ABC, and MFS transcripts expressed by *Citrus* spp., molecular weight, isoelectric point, sequence length, subcellular localization, and putative gene name from *C. sinensis*, Table S4: MATE, ABC, and MFS expressed genes by *Citrus* spp., including gene duplication status, number of putative isoforms produced, and the annotation, Table S5: MATE, ABC, and MFS differentially expressed, Log_2_FC values according to the time points after bacterial inoculation (HAI) and species, p-value, and annotation.
