# Supplementary material for: A Genomic and Transcriptomic Overview of MATE, ABC, and MFS Transporters in Citrus sinensis Interaction with Xanthomonas citri subsp. citri
Source: Plants (Basel). 2020 Jun 25;9(6):794. doi: 10.3390/plants9060794 (PMC7356318; doi:10.3390/plants9060794)
Supplement: Supplementary file 1 [file plants-09-00794-s001.pdf]

plants

MDPI

*Article*

# A Genomic and Transcriptomic Overview of MATE, ABC, and MFS Transporters in *Citrus sinensis* Interaction with *Xanthomonas citri* subsp. *citri*

**Maria H. M. Julião, Saura R. Silva, Jesus A. Ferro and Alessandro M. Varani ***

Department of Technology, School of Agricultural and Veterinary Sciences, São Paulo State University,14884-900, Jaboticabal, Brazil; m.juliao@unesp.br (M.H.M.J.); saura.silva@unesp.br (S.R.S.); jesus.ferro@unesp.br (J.A.F.)
* Correspondence: alessandro.varani@unesp.br

## Supplementary Materials

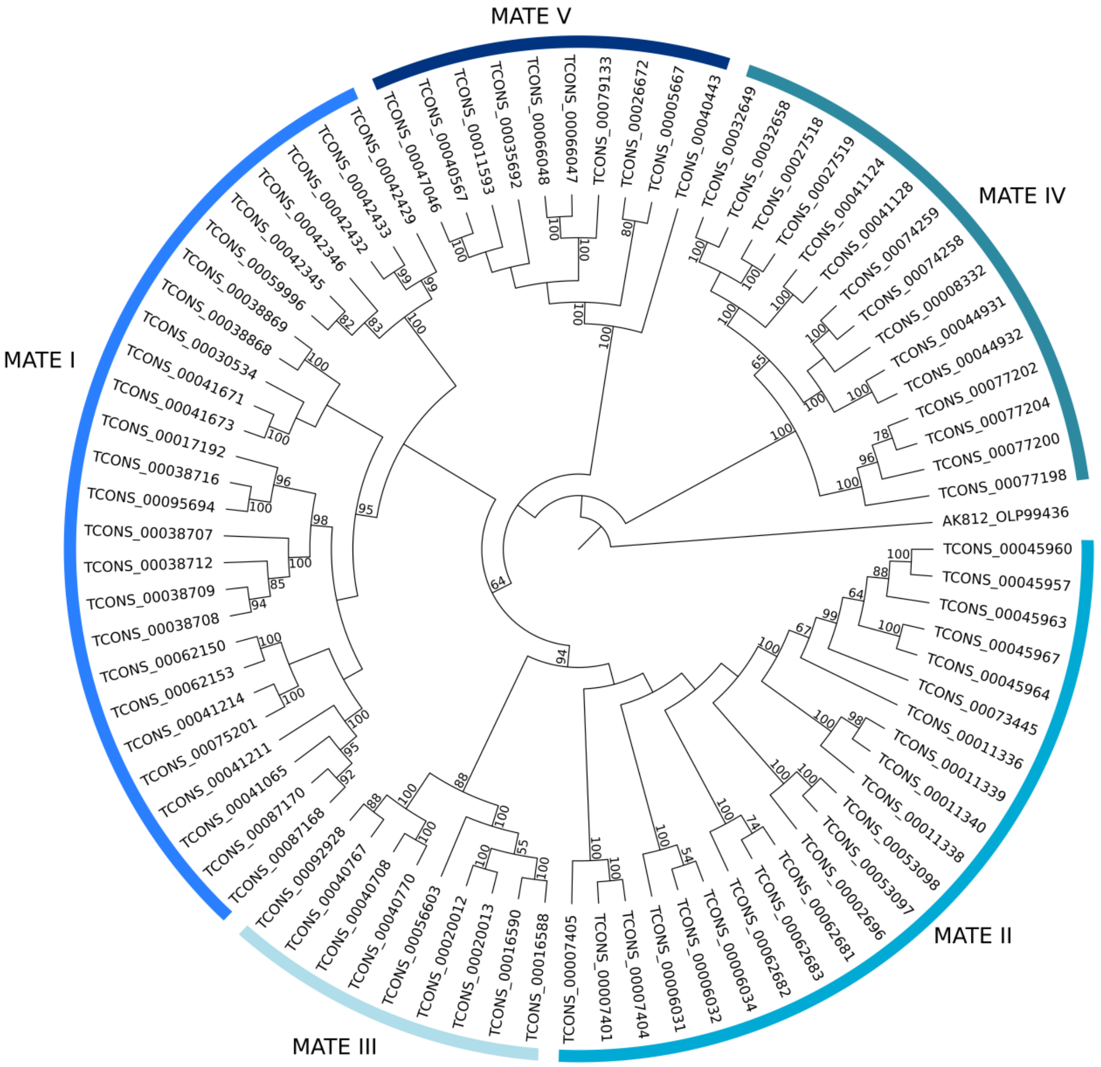


**Figure S1.** Maximum likelihood tree of MATE amino acid sequences expressed by *Citrus* spp. identified in the citrus reference transcriptome. Numbers above the branches represent bootstrap values. AK812_OLP99436 was used as outgroup (*Symbiodinium microadriaticum str. CCMP2467, Gene: slc47a1, multidrug, and toxin extrusion protein 1*).

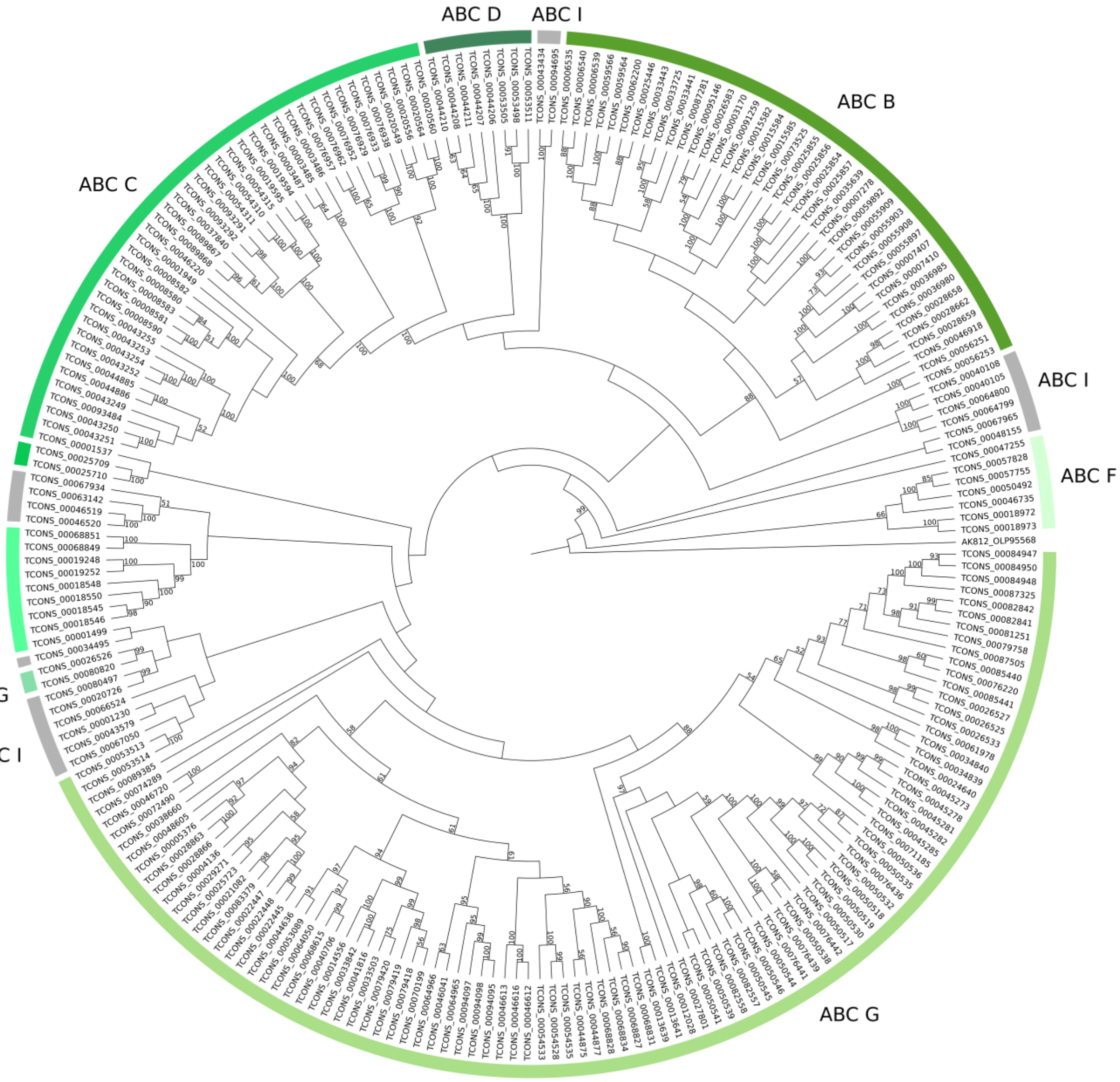


**Figure S2.** Maximum likelihood tree of ABC amino acid sequences expressed by *Citrus* spp. identified in the citrus reference transcriptome. Numbers above the branches represent bootstrap values. DB43_AL00090 was used as outgroup (*Parachlamydia acanthamoebae, gene ywtG, putative metabolite transport protein YwtG*).

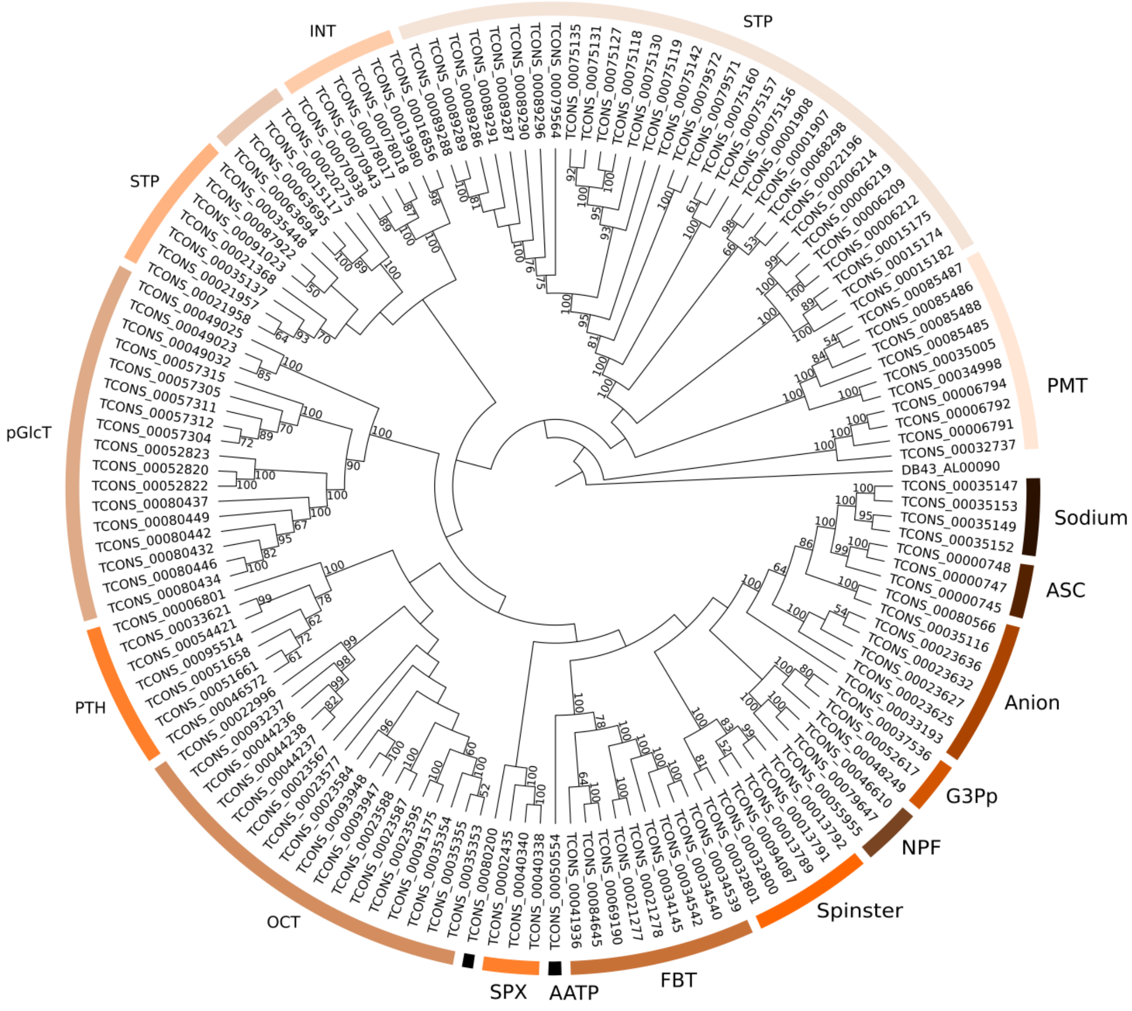


**Figure S3.** Maximum likelihood tree of MFS amino acid sequences expressed by *Citrus* spp. identified in the citrus reference transcriptome. Numbers above the branches represent bootstrap values. DB43_AL00090 was used as outgroup. (*Parachlamydia acanthamoebae, gene ywtG, putative metabolite transport protein YwtG*).

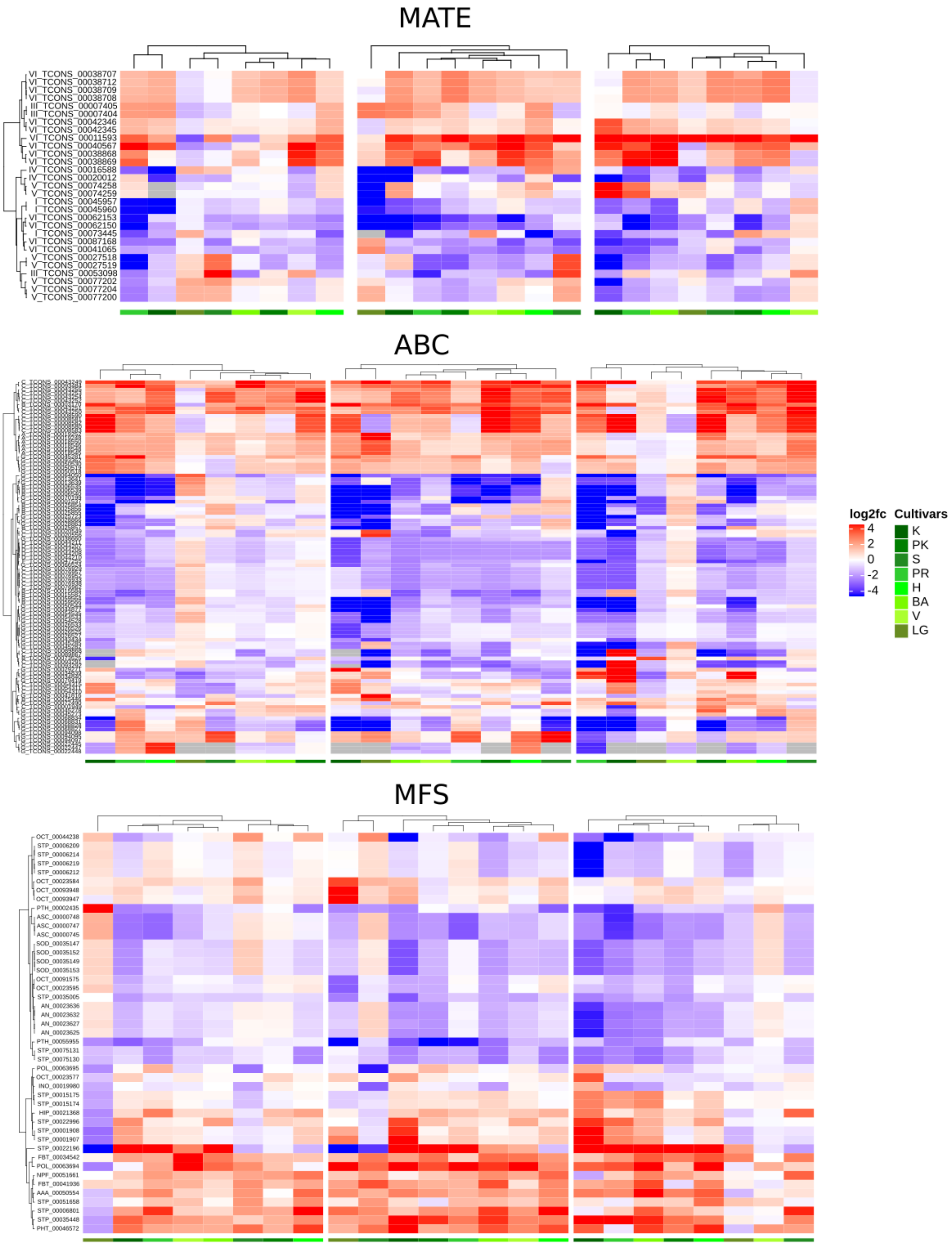


**Figure S4.** Clustered heatmaps of MATE, ABC, and MFS differentially expressed transcripts on at least one HAI in a species. K: 'Kumquat', PK: 'Ponkan', S: 'Satsuma', PR: 'Pera Rio', H: 'Hamlin', BA: 'Bahia', V: 'Valencia', LG: 'Galego', and HAI: Hours after bacterial inoculation. The identification of each transcript is given by the abbreviation of its respective subfamily followed by its identification number in the CRT.

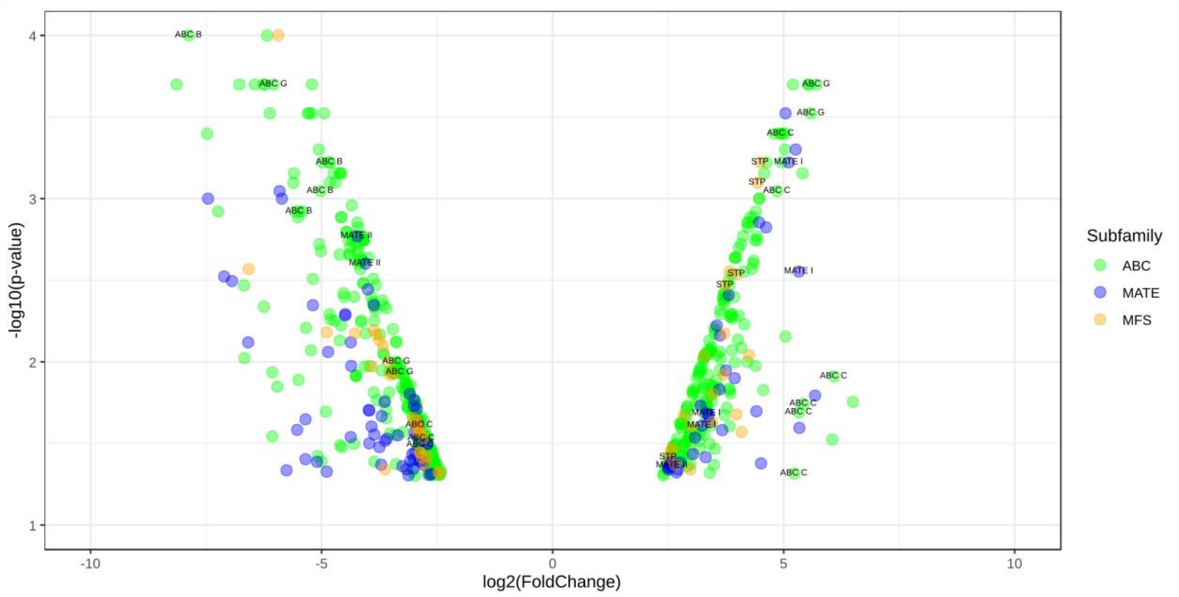


**Figure S5.** Volcano plot of MATE, ABC, and MFS differentially expressed transcripts. The 50 differentially expressed transcripts (Derived from the 4 MATE, 10 ABC, and 3 MFS that are potentially related to plant-defense mechanisms) associated with the plant membrane transporters gene candidates who most significantly impacted the plant defense responses under Citrus-Xac infection are annotated in the plot.

**Table S1.** Domains and motifs related to MATE, ABC, and MFS proteins used in the proteins manually verification.

| Family | Accession | Name | Integrated Signatures | GO Terms |
|---|---|---|---|---|
| ABC | IPR000515 | ABC transporter type 1, transmembrane domain MetI-like | PF00528, cd06261, PS50928 | GO:0055085,GO:0016020 |
| | IPR002491 | ABC transporter periplasmic binding domain | PF01497, PS50983 | |
| | IPR003439 | ABC transporter-like | PF00005, PS50893 | GO:0005524,GO:0016887 |
| | IPR003760 | ABC transporter substrate-binding protein PnrA-like | PF02608 | GO:0005886 |
| | IPR003838 | ABC transporter permease protein domain | PF02687 | GO:0016020 |
| | IPR010929 | CDR ABC transporter | PF06422 | GO:0005524,GO:0042626,GO:0055085,GO:0016021 |
| | IPR011527 | ABC transporter type 1, transmembrane domain | PF00664, PF06472, PF13748, PS50929 | GO:0005524,GO:0042626,GO:0055085,GO:0016021 |
| | IPR013563 | Oligopeptide/dipeptide ABC transporter, C-terminal | TIGR01727,PF08352 | GO:0000166,GO:0005524,GO:0015833 |
| | IPR013581 | Plant PDR ABC transporter associated | PF08370 | |
| | IPR022182 | ABC transporter phosphate permease PstC, N-terminal domain | PF12501 | GO:0006817 |
| | IPR032524 | ABC transporter Uup, C-terminal | PF16326 | GO:0003677 |
| | IPR032550 | PBP-dependent ABC transporters TM subunit, N-terminal | PF16296 | |
| | IPR040856 | Glucose ABC transporter, C-terminal | PF17847 | |
| MATE | IPR002528 | Multi antimicrobial extrusion protein | PF01554,TIGR00797,PIRSF006603 | GO:0015297,GO:0042910,GO:0055085,GO:0016020 |
| MFS | IPR036259 | MFS transporter superfamily | SSF103473 | |
| | IPR011701 | Major facilitator superfamily | PF07690 | GO:0022857,GO:0055085 |
| | IPR020846 | Major facilitator superfamily domain | PS50850 | GO:0022857 |
| | IPR020846 | Major facilitator superfamily domain | PS50850 | GO:0022857 |
| | IPR024989 | Major facilitator superfamily associated domain | PF12832 | |
| | IPR005828 | Major facilitator, sugar transporter-like | PF00083 | GO:0022857,GO:0055085,GO:0016021 |

| | | | |
|---|---|---|---|
| IPR000109 | Proton-dependent oligopeptide transporter family | PTHR11654 PF00854 | GO:0022857,GO:0055085,GO:0016020 |
| IPR004768 | Oligopeptide transporter | TIGR00926 | GO:0035673,GO:0006857,GO:0016021 |
| IPR004813 | Oligopeptide transporter, OPT superfamily | TIGR00728,PF03169 | GO:0055085 |
| IPR004814 | Oligopeptide transporter OPT | TIGR00733 | |
| IPR004316 | SWEET sugar transporter | PF03083 | GO:0016021 |
| IPR005828 | Major facilitator, sugar transporter-like | PF00083 | GO:0022857,GO:0055085,GO:0016021 |
| IPR007271 | Nucleotide-sugar transporter | PIRSF005799, PF04142, PTHR10231 | GO:0015165,GO:0090481,GO:0000139,GO:0016021 |
| IPR012404 | Nucleotide-sugar transporter-related | PIRSF036436 | GO:0016021 |
| IPR018180 | Sugar transporter SWEET1 | PTHR10791:SF44, PTHR10791:SF155 | GO:0051119 |
| IPR025601 | ATP-binding sugar transporter-like protein | PF13856 | |
| IPR008509 | Molybdate-anion transporter | PF05631, PTHR23516 | GO:0015098,GO:0015689,GO:0016021 |

**Table S2.** Estimation of non-synonymous and synonymous substitutions mean dissimilarity for each sub-family ($\delta = d_N - d_S$ and $\omega = d_N / d_S$).

| Gene families | Nei-Gojobori method | SLAC method | |
|---|---|---|---|
| | δ | *p*-Value | ω |
| ABC A | −10.99 | 1.00E−10 | 0.0819 |
| ABC B | −27.98 | 1.00E−10 | 170 |
| ABC C | −34.90 | 1.00E−10 | 202 |
| ABC D | −8.66 | 1.00E−10 | -* |
| ABC E | - | - | - |
| ABC F | −9.18 | 1.00E−10 | 194 |
| ABC G | −24.24 | 1.00E−10 | 215 |
| ABC I | −7.87 | 1.00E−10 | 112 |
| MATE I | −23.60 | 1.00E−10 | 244 |
| MATE II | −27.03 | 1.00E−10 | 230 |
| MATE III | −20.86 | 1.00E−10 | 224 |
| MATE IV | −16.31 | 1.00E−10 | 189 |
| MATE V | −18.31 | 1.00E−10 | 0.0639 |
| MFS Anion | −12.83 | 1.00E−10 | 145 |
| MFS Ascorbate | −27.67 | 1.00E−10 | 146 |
| MFS FBT | −13.85 | 1.00E−10 | 187 |
| MFS G3Pp | −18.47 | 1.00E−10 | 86 |
| MFS Inositol | −11.09 | 1.00E−10 | 194 |
| MFS OTH | - | - | - |
| MFS PMT | −16.93 | 1.00E−10 | -* |
| MFS Polyol | −25.47 | 1.00E−10 | 128 |
| MFS PTH | −32.21 | 1.00E−10 | 0.0934 |
| MFS Spinster | −10.06 | 1.00E−10 | 299 |
| MFS STP | −18.54 | 1.00E−10 | 291 |

** SLAC can only be perfomed with a minimum of two sequences. In the table are reported: the average nucleotide diversity (π) of the ABC subfamilies analyzed using the Nei-Gojobori and SLAC methods and the selection ($dN > dS$ ;$dN/dS > 1$) or negative selection($dN < dS$ ; $dN/dS < 1$). of strict-neutrality ($dN = dS$ ;$dN/dS = 1$) in favor of the alternative hypothesis positive probability ($p$-Value $< 0.05$) of rejecting the null hypothesis.

**Table S3.** MATE, ABC, and MFS transcripts expressed by *Citrus* spp., molecular weight, isoelectric point, sequence length, subcellular localization, and putative gene name from *C. sinensis*.

| Transcript | Molecular Weigh | Isoelectric Point | Sequence Length | Sublocalization | Annotation | Subfamily | Putative Gene |
|---|---|---|---|---|---|---|---|
| TCONS_00095694 | 11907.7 | 4.1957 | 111 | chloroplast | DTX_14 | MATE I | Cs2g13500 |
| TCONS_00087170 | 27328.3 | 4.5576 | 244 | plasma membrane | DTX_8 | MATE I | Cs6g18200 |
| TCONS_00087168 | 20442.3 | 8.3374 | 183 | E.R. | DTX_8 | MATE I | Cs6g18200 |
| TCONS_00075201 | 49998.5 | 8.6245 | 461 | plasma membrane | DTX_12 | MATE I | Cs2g31280 |
| TCONS_00062153 | 31277.8 | 8.6791 | 287 | vacuole | DTX_14 | MATE I | Cs5g20110_m |
| TCONS_00062150 | 49446.9 | 8.9154 | 454 | plasma membrane | DTX_14 | MATE I | Cs5g19630 |
| TCONS_00059996 | 10086.6 | 6.7034 | 92 | E.R. | DTX_19 | MATE I | Cs1g07550 |
| TCONS_00042433 | 56145.1 | 8.2104 | 506 | plasma membrane | DTX_19 | MATE I | Cs1g07550 |
| TCONS_00042432 | 34605.6 | 9.2806 | 309 | plasma membrane | DTX_19 | MATE I | Cs1g07550 |
| TCONS_00042429 | 19791.2 | 7.9528 | 179 | vacuole | DTX_19 | MATE I | Cs1g07550 |
| TCONS_00042346 | 52550.9 | 6.6813 | 481 | plasma membrane | DTX_19 | MATE I | Cs1g07540 |
| TCONS_00042345 | 40783 | 6.4827 | 371 | E.R. | DTX_19 | MATE I | Cs1g07540 |
| TCONS_00041673 | 36382.5 | 8.3743 | 328 | plasma membrane | DTX_16 | MATE I | orange1.1t00013 |
| TCONS_00041671 | 58872.5 | 7.8564 | 538 | plasma membrane | DTX_16 | MATE I | orange1.1t00013 |
| TCONS_00041214 | 28026.8 | 8.5859 | 256 | plasma membrane | DTX_12 | MATE I | Cs6g18160 |
| TCONS_00041211 | 56198.5 | 7.1551 | 513 | plasma membrane | DTX_8 | MATE I | Cs6g18150 |
| TCONS_00041065 | 24054.1 | 8.2445 | 223 | vacuole | DTX_8 | MATE I | Cs6g18200 |
| TCONS_00038869 | 54635.3 | 7.7156 | 502 | plasma membrane | DTX_16 | MATE I | Cs2g13530 |
| TCONS_00038868 | 41382.3 | 8.2776 | 375 | plasma membrane | DTX_16 | MATE I | Cs2g13530 |
| TCONS_00038716 | 54731.5 | 5.0645 | 500 | plasma membrane | DTX_14 | MATE I | Cs2g13500 |
| TCONS_00038712 | 35180.5 | 5.2449 | 320 | plasma membrane | DTX_12 | MATE I | Cs2g13510 |
| TCONS_00038709 | 50403.4 | 7.9799 | 461 | plasma membrane | DTX_12 | MATE I | Cs2g13510 |
| TCONS_00038708 | 54657.3 | 8.1167 | 502 | plasma membrane | DTX_12 | MATE I | Cs2g13510 |
| TCONS_00038707 | 27145.1 | 6.8017 | 244 | plasma membrane | DTX_12 | MATE I | Cs2g13510 |
| TCONS_00030534 | 52006.4 | 7.9821 | 476 | plasma membrane | DTX_16 | MATE I | Cs5g06100 |
| TCONS_00017192 | 27457.8 | 4.5817 | 250 | plasma membrane | DTX_14 | MATE I | Cs2g13501_m |
| TCONS_00073445 | 52576.4 | 6.5865 | 476 | plasma membrane | DTX_27 | MATE II | Cs5g21940 |
| TCONS_00062683 | 55735.5 | 5.2952 | 507 | plasma membrane | DTX_33 | MATE II | Cs5g28190 |
| TCONS_00062682 | 57912.2 | 7.837 | 525 | plasma membrane | DTX_33 | MATE II | Cs5g28190 |
| TCONS_00062681 | 30391.1 | 7.1433 | 278 | vacuole | DTX_33 | MATE II | Cs5g28190 |
| TCONS_00053098 | 55726.9 | 6.7161 | 509 | plasma membrane | DTX_35 | MATE II | Cs9g19420 |
| TCONS_00053097 | 52762.7 | 8.5186 | 482 | plasma membrane | DTX_35 | MATE II | Cs9g19430 |
| TCONS_00045967 | 54929.9 | 6.3298 | 499 | plasma membrane | DTX_27 | MATE II | Cs1g20120 |
| TCONS_00045964 | 37222 | 5.2667 | 335 | E.R. | DTX_27 | MATE II | Cs1g20120 |
| TCONS_00045963 | 27335.1 | 7.9173 | 244 | plasma membrane | DTX_27 | MATE II | Cs1g20120 |
| TCONS_00045960 | 54381.5 | 8.216 | 499 | plasma membrane | DTX_27 | MATE II | Cs1g20130 |
| TCONS_00045957 | 37170 | 7.8896 | 338 | plasma membrane | DTX_27 | MATE II | Cs1g20130 |
| TCONS_00011340 | 53151 | 6.4797 | 487 | plasma membrane | DTX_27 | MATE II | Cs3g17660 |

| | | | | | | | |
|---|---|---|---|---|---|---|---|
| TCONS_00011339 | 36043.5 | 7.4298 | 323 | plasma membrane | DTX_27 | MATE II | Cs3g17660 |
| TCONS_00011338 | 19623.3 | 7.1645 | 183 | vacuole | DTX_27 | MATE II | Cs3g17660 |
| TCONS_00011336 | 56101 | 7.9652 | 512 | plasma membrane | DTX_27 | MATE II | Cs3g17670 |
| TCONS_00007405 | 51604.6 | 10.1562 | 464 | plasma membrane | DTX_29 | MATE II | Cs7g09190 |
| TCONS_00007404 | 57249.7 | 6.969 | 527 | plasma membrane | DTX_29 | MATE II | Cs7g09190 |
| TCONS_00007401 | 37381.2 | 6.8374 | 341 | plasma membrane | DTX_29 | MATE II | Cs7g09190 |
| TCONS_00006034 | 43126.5 | 7.8135 | 398 | vacuole | DTX_33 | MATE II | Cs3g24330 |
| TCONS_00006032 | 25481.8 | 8.4818 | 230 | vacuole | DTX_33 | MATE II | Cs3g24330 |
| TCONS_00006031 | 18326.5 | 6.8755 | 173 | vacuole | DTX_33 | MATE II | Cs3g24330 |
| TCONS_00002696 | 57273 | 5.4118 | 524 | plasma membrane | DTX_34 | MATE II | orange1.1t00390 |
| TCONS_00092928 | 33423.7 | 8.6776 | 303 | vacuole | DTX_41 | MATE III | Cs2g15080 |
| TCONS_00056603 | 59547.2 | 8.1005 | 547 | plasma membrane | DTX_40 | MATE III | Cs2g21460 |
| TCONS_00040770 | 23853 | 6.4396 | 219 | E.R. | DTX_41 | MATE III | Cs2g15080 |
| TCONS_00040767 | 55481.7 | 7.5818 | 505 | plasma membrane | DTX_41 | MATE III | Cs2g15120 |
| TCONS_00040708 | 28640.7 | 7.2885 | 257 | vacuole | DTX_41 | MATE III | Cs2g15610 |
| TCONS_00020013 | 27074.2 | 5.6849 | 252 | vacuole | DTX_40 | MATE III | Cs2g08000 |
| TCONS_00020012 | 54024.1 | 7.6783 | 501 | plasma membrane | DTX_40 | MATE III | Cs2g08000 |
| TCONS_00016590 | 23209.7 | 9.9123 | 215 | vacuole | DTX_40 | MATE III | Cs2g03200 |
| TCONS_00016588 | 56297.2 | 6.0341 | 519 | plasma membrane | DTX_40 | MATE III | Cs2g03220 |
| TCONS_00077204 | 60550.5 | 8.6079 | 557 | chloroplast | DTX_46 | MATE IV | Cs8g08470 |
| TCONS_00077202 | 28053.4 | 7.6464 | 257 | chloroplast | DTX_46 | MATE IV | Cs8g08470 |
| TCONS_00077200 | 43554.2 | 8.5898 | 398 | plasma membrane | DTX_46 | MATE IV | Cs8g08470 |
| TCONS_00077198 | 59496.7 | 7.7824 | 548 | plasma membrane | DTX_46 | MATE IV | Cs8g08480 |
| TCONS_00074259 | 40292.9 | 9.7167 | 375 | plasma membrane | DTX_42 | MATE IV | orange1.1t03403 |
| TCONS_00074258 | 58134.8 | 7.4094 | 538 | plasma membrane | DTX_42 | MATE IV | orange1.1t03403 |
| TCONS_00044932 | 57227.2 | 7.8844 | 535 | plasma membrane | DTX_42 | MATE IV | Cs2g30270 |
| TCONS_00044931 | 31956.1 | 5.1502 | 299 | plasma membrane | DTX_42 | MATE IV | Cs2g30270 |
| TCONS_00041128 | 29661.1 | 10.5478 | 279 | chloroplast | DTX_44 | MATE IV | Cs6g09270 |
| TCONS_00041124 | 44027.1 | 10.24 | 413 | chloroplast | DTX_44 | MATE IV | Cs6g09270 |
| TCONS_00032658 | 30083.9 | 10.0467 | 282 | plasma membrane | DTX_45 | MATE IV | Cs8g01660 |
| TCONS_00032649 | 63766.5 | 9.2068 | 602 | plasma membrane | DTX_45 | MATE IV | Cs8g01660 |
| TCONS_00027519 | 35806.2 | 10.3576 | 330 | chloroplast | DTX_45 | MATE IV | Cs1g26350 |
| TCONS_00027518 | 65503.2 | 9.2159 | 611 | E.R. | DTX_45 | MATE IV | Cs1g26350 |
| TCONS_00008332 | 26412.6 | 10.2381 | 246 | plasma membrane | DTX_43 | MATE IV | Cs1g26350 |
| TCONS_00079133 | 50154 | 7.6444 | 465 | vacuole | DTX_53 | MATE V | Cs7g16290_m |
| TCONS_00066048 | 53856.7 | 8.5624 | 496 | plasma membrane | DTX_53 | MATE V | Cs6g09880 |
| TCONS_00066047 | 36822.5 | 8.4209 | 337 | plasma membrane | DTX_53 | MATE V | Cs6g09880 |
| TCONS_00047046 | 57180.6 | 7.7634 | 525 | plasma membrane | DTX_49 | MATE V | Cs9g16870 |
| TCONS_00040567 | 56054.2 | 7.4348 | 513 | plasma membrane | DTX_49 | MATE V | Cs5g10270 |
| TCONS_00040443 | 58872.7 | 8.3841 | 543 | plasma membrane | DTX_56 | MATE V | Cs5g09710 |
| TCONS_00035692 | 57003.4 | 8.0683 | 531 | plasma membrane | DTX_51 | MATE V | Cs4g15415_m |
| TCONS_00026672 | 54470.4 | 8.2021 | 501 | plasma membrane | DTX_55 | MATE V | Cs9g04120 |
| TCONS_00011593 | 61807.3 | 7.8303 | 586 | plasma membrane | DTX_48 | MATE V | Cs3g16650 |
| TCONS_00005667 | 37133 | 6.4647 | 336 | plasma membrane | DTX_54 | MATE V | Cs3g22600 |

| Transcript | Molecular Weigh | Isoelectric Point | Sequence Length | Sublocalization | Subfamily | Putative Gene |
|---|---|---|---|---|---|---|
| TCONS_00068851 | 188525,17 | 7,0271 | 1695 | plasma membrane | ABC A | orange1.1t02318 |
| TCONS_00068849 | 205029,33 | 7,8267 | 1841 | plasma membrane | ABC A | orange1.1t02318 |
| TCONS_00019252 | 78376,39 | 7,2099 | 706 | plasma membrane | ABC A | Cs5g04120 |
| TCONS_00019248 | 53839,28 | 5,2737 | 483 | plasma membrane | ABC A | Cs5g04120 |
| TCONS_00018550 | 70976,26 | 8,4755 | 637 | plasma membrane | ABC A | Cs5g04110 |
| TCONS_00018548 | 65454,57 | 7,1179 | 589 | plasma membrane | ABC A | Cs5g04110 |
| TCONS_00018546 | 106798,08 | 8,0549 | 956 | plasma membrane | ABC A | Cs5g04110 |
| TCONS_00018545 | 101276,4 | 7,2179 | 908 | plasma membrane | ABC A | Cs5g04110 |
| TCONS_00095146 | 51233,21 | 7,3486 | 478 | plasma membrane | ABC B | Cs2g04080 |
| TCONS_00091259 | 76008,04 | 7,8674 | 703 | plasma membrane | ABC B | Cs9g01170 |
| TCONS_00087281 | 124578,86 | 7,0244 | 1139 | plasma membrane | ABC B | Cs9g11040 |
| TCONS_00073525 | 171667,2 | 9,2634 | 1560 | plasma membrane | ABC B | Cs1g16850 |
| TCONS_00062200 | 148975,56 | 8,3082 | 1359 | plasma membrane | ABC B | orange1.1t00937 |
| TCONS_00059892 | 137715,87 | 9,0217 | 1255 | plasma membrane | ABC B | Cs4g11230 |
| TCONS_00059566 | 99686,33 | 8,6588 | 903 | plasma membrane | ABC B | Cs3g09820 |
| TCONS_00059564 | 142127,25 | 8,645 | 1292 | plasma membrane | ABC B | Cs3g09820 |
| TCONS_00056253 | 77986,37 | 9,6879 | 702 | plasma membrane | ABC B | Cs1g08880 |
| TCONS_00056251 | 81372,51 | 10,0389 | 731 | plasma membrane | ABC B | Cs1g08880 |
| TCONS_00055909 | 79846,69 | 8,3853 | 709 | plasma membrane | ABC B | Cs2g27440 |
| TCONS_00055908 | 46192,76 | 7,4341 | 407 | mitocondria | ABC B | Cs2g27440 |
| TCONS_00055903 | 76001,21 | 8,5797 | 677 | plasma membrane | ABC B | Cs2g27440 |
| TCONS_00055897 | 52723,24 | 9,3561 | 467 | plasma membrane | ABC B | Cs2g27440 |
| TCONS_00046918 | 86191,02 | 9,387 | 791 | plasma membrane | ABC B | Cs9g16780 |
| TCONS_00036985 | 64853,55 | 8,4383 | 611 | plasma membrane | ABC B | Cs5g12150 |
| TCONS_00036980 | 68987,4 | 8,6972 | 647 | plasma membrane | ABC B | Cs5g12150 |
| TCONS_00035639 | 134759,42 | 9,172 | 1230 | plasma membrane | ABC B | Cs4g15420 |
| TCONS_00033725 | 46021,68 | 9,9774 | 412 | chloroplast | ABC B | Cs7g28610 |

| | | | | | | |
|---|---|---|---|---|---|---|
| TCONS_00033443 | 144915,27 | 7,2087 | 1303 | plasma membrane | ABC B | Cs7g28610 |
| TCONS_00033441 | 82815,6 | 5,4403 | 747 | plasma membrane | ABC B | Cs7g28610 |
| TCONS_00028662 | 63740,34 | 8,6188 | 582 | plasma membrane | ABC B | Cs6g14480 |
| TCONS_00028659 | 64880,75 | 9,341 | 592 | plasma membrane | ABC B | Cs6g14480 |
| TCONS_00028658 | 17715,28 | 7,1293 | 160 | cytosol | ABC B | Cs6g14480 |
| TCONS_00026583 | 133450,29 | 7,2295 | 1236 | plasma membrane | ABC B | Cs9g01170_a |
| TCONS_00025857 | 136076,59 | 7,5088 | 1247 | plasma membrane | ABC B | Cs6g20290 |
| TCONS_00025856 | 112925,74 | 8,4603 | 1027 | plasma membrane | ABC B | Cs6g20280 |
| TCONS_00025855 | 73470,79 | 8,2938 | 668 | vacuole | ABC B | Cs6g20270 |
| TCONS_00025854 | 71274,68 | 7,2363 | 647 | plasma membrane | ABC B | Cs6g20270 |
| TCONS_00025446 | 136438,12 | 8,5551 | 1253 | plasma membrane | ABC B | Cs6g21110 |
| TCONS_00015585 | 68155,5 | 7,0146 | 632 | plasma membrane | ABC B | Cs2g04070 |
| TCONS_00015584 | 144638,8 | 8,1973 | 1332 | plasma membrane | ABC B | Cs2g04080 |
| TCONS_00015582 | 91599,66 | 9,194 | 837 | plasma membrane | ABC B | Cs2g04080 |
| TCONS_00007410 | 37102,54 | 10,0755 | 326 | plasma membrane | ABC B | Cs7g09160 |
| TCONS_00007407 | 96751,66 | 9,7628 | 854 | plasma membrane | ABC B | Cs7g09160 |
| TCONS_00007278 | 133938,42 | 9,0203 | 1224 | plasma membrane | ABC B | Cs7g09730 |
| TCONS_00006540 | 85588,31 | 7,1752 | 789 | plasma membrane | ABC B | Cs3g26380 |
| TCONS_00006539 | 136639,36 | 8,4429 | 1260 | plasma membrane | ABC B | Cs3g26380 |
| TCONS_00006535 | 88318,42 | 7,5953 | 819 | plasma membrane | ABC B | Cs3g26380 |
| TCONS_00003170 | 140125,78 | 6,6978 | 1299 | plasma membrane | ABC B | orange1.1t01769_a |
| TCONS_00093484 | 56051,65 | 6,1313 | 508 | plasma membrane | ABC C | Cs4g09150 |
| TCONS_00093292 | 19006,58 | 10,4364 | 161 | plasma membrane | ABC C | Cs1g18450 |
| TCONS_00093291 | 21974,13 | 9,6543 | 191 | plasma membrane | ABC C | Cs1g18450 |
| TCONS_00089868 | 61573,8 | 8,5267 | 555 | chloroplast | ABC C | Cs1g18450 |
| TCONS_00089867 | 37479,06 | 7,5084 | 332 | nucleus | ABC C | Cs1g18450 |
| TCONS_00076962 | 160756,62 | 6,6066 | 1433 | plasma membrane | ABC C | orange1.1t02765 |
| TCONS_00076957 | 164017,51 | 6,6624 | 1465 | plasma membrane | ABC C | orange1.1t02765 |

| | | | | | | |
|---|---|---|---|---|---|---|
| TCONS_00076952 | 110325,28 | 6,7756 | 980 | plasma membrane | ABC C | orange1.1t02765 |
| TCONS_00076938 | 94373,23 | 7,9232 | 835 | plasma membrane | ABC C | orange1.1t02765 |
| TCONS_00076933 | 59151,17 | 6,8578 | 530 | plasma membrane | ABC C | orange1.1t02765 |
| TCONS_00076929 | 30369,87 | 7,6572 | 273 | chloroplast | ABC C | orange1.1t02765 |
| TCONS_00054315 | 137138,23 | 8,3994 | 1234 | plasma membrane | ABC C | Cs9g18660 |
| TCONS_00054311 | 165306,37 | 8,1658 | 1485 | plasma membrane | ABC C | Cs9g18660 |
| TCONS_00054310 | 99858,66 | 8,8131 | 895 | plasma membrane | ABC C | Cs9g18660 |
| TCONS_00046220 | 149463,19 | 7,5066 | 1343 | plasma membrane | ABC C | Cs5g28990 |
| TCONS_00044886 | 129092,47 | 7,5305 | 1156 | plasma membrane | ABC C | Cs1g13700 |
| TCONS_00044885 | 61122,26 | 8,0095 | 547 | plasma membrane | ABC C | Cs4g09140 |
| TCONS_00043255 | 165906,5 | 6,5071 | 1496 | plasma membrane | ABC C | Cs4g09120 |
| TCONS_00043254 | 166553,59 | 7,1469 | 1495 | plasma membrane | ABC C | Cs4g09130 |
| TCONS_00043253 | 98197,19 | 5,9232 | 885 | plasma membrane | ABC C | Cs4g09130 |
| TCONS_00043252 | 163674,91 | 7,1965 | 1469 | plasma membrane | ABC C | Cs1g13720 |
| TCONS_00043251 | 145109,16 | 6,4705 | 1300 | nucleus | ABC C | Cs4g09140 |
| TCONS_00043250 | 47502,63 | 5,656 | 425 | chloroplast | ABC C | Cs4g09140 |
| TCONS_00043249 | 56270,37 | 9,0061 | 501 | plasma membrane | ABC C | Cs4g09150 |
| TCONS_00037840 | 164345,38 | 8,7054 | 1475 | plasma membrane | ABC C | Cs1g18460 |
| TCONS_00020564 | 127020,95 | 5,5519 | 1142 | plasma membrane | ABC C | Cs2g09550 |
| TCONS_00020560 | 183757,62 | 6,5966 | 1635 | plasma membrane | ABC C | Cs2g09550 |
| TCONS_00020556 | 147399,58 | 9,2353 | 1307 | plasma membrane | ABC C | Cs2g09560 |
| TCONS_00020549 | 61168,52 | 9,6605 | 543 | plasma membrane | ABC C | Cs7g32530 |
| TCONS_00019595 | 141203,84 | 6,6556 | 1261 | plasma membrane | ABC C | Cs5g02710 |
| TCONS_00019594 | 89232,09 | 6,1392 | 799 | plasma membrane | ABC C | Cs5g02710 |
| TCONS_00008590 | 87945,08 | 9,2257 | 781 | plasma membrane | ABC C | Cs7g10200 |
| TCONS_00008583 | 165591,13 | 8,4506 | 1472 | plasma membrane | ABC C | Cs7g10200 |
| TCONS_00008582 | 99974,37 | 6,9255 | 893 | plasma membrane | ABC C | Cs7g10200 |
| TCONS_00008581 | 87533,28 | 8,4347 | 779 | plasma membrane | ABC C | Cs7g10200 |

| | | | | | | |
|---|---|---|---|---|---|---|
| TCONS_00008580 | 162302,14 | 8,4242 | 1444 | plasma membrane | ABC C | Cs7g10200 |
| TCONS_00003487 | 90298,15 | 6,2127 | 807 | plasma membrane | ABC C | Cs5g33060 |
| TCONS_00003486 | 169310,85 | 7,6464 | 1513 | plasma membrane | ABC C | Cs5g33060 |
| TCONS_00003485 | 48814,36 | 7,1194 | 435 | chloroplast | ABC C | Cs5g33060 |
| TCONS_00001949 | 168678,36 | 7,849 | 1514 | plasma membrane | ABC C | Cs1g02710 |
| TCONS_00053511 | 57332,25 | 9,1375 | 513 | cytosol | ABC D | Cs1g01510 |
| TCONS_00053505 | 155020,14 | 9,3954 | 1384 | plasma membrane | ABC D | Cs1g01510 |
| TCONS_00053498 | 96849,32 | 7,3367 | 865 | cytosol | ABC D | Cs1g01510 |
| TCONS_00044211 | 88875,29 | 7,4742 | 788 | chloroplast | ABC D | orange1.1t03223 |
| TCONS_00044210 | 35136,86 | 7,4169 | 314 | nucl_plas | ABC D | orange1.1t03223 |
| TCONS_00044208 | 53120,39 | 6,0318 | 472 | chloroplast | ABC D | orange1.1t03223 |
| TCONS_00044207 | 58140,61 | 9,0197 | 510 | peroxissome | ABC D | orange1.1t03223 |
| TCONS_00044206 | 59869,57 | 5,4323 | 532 | E.R. | ABC D | orange1.1t03223 |
| TCONS_00025710 | 72131,23 | 7,9791 | 639 | chloroplast_mitocondrial | ABC E | Cs6g19410 |
| TCONS_00025709 | 37972,64 | 6,2521 | 335 | cytosol | ABC E | Cs6g19410 |
| TCONS_00057828 | 14732,98 | 10,6387 | 134 | nucleus | ABC F | Cs9g09560 |
| TCONS_00057755 | 17521,35 | 8,1524 | 158 | golgi | ABC F | Cs1g13980 |
| TCONS_00050492 | 83228,16 | 6,2344 | 747 | cytosol | ABC F | Cs1g13980 |
| TCONS_00047255 | 79816,03 | 7,9346 | 708 | chloroplast | ABC F | orange1.1t04404 |
| TCONS_00046735 | 66209,91 | 6,6092 | 593 | cytosol | ABC F | Cs8g15910 |
| TCONS_00018973 | 29364,85 | 8,145 | 266 | mitocondria | ABC F | Cs5g01810 |
| TCONS_00018972 | 12084,95 | 10,238 | 114 | chloroplast | ABC F | Cs5g01810 |
| TCONS_00094098 | 41685,77 | 6,4893 | 369 | plasma membrane | ABC G | orange1.1t01993 |
| TCONS_00094097 | 29178,53 | 7,8791 | 255 | plasma membrane | ABC G | orange1.1t01993 |
| TCONS_00094095 | 45512,18 | 6,8219 | 401 | plasma membrane | ABC G | orange1.1t01993 |
| TCONS_00089385 | 15598,15 | 5,5119 | 149 | vacuole | ABC G | Cs1g01420 |
| TCONS_00087505 | 26682,38 | 6,9439 | 233 | plasma membrane | ABC G | Cs6g01131_a |
| TCONS_00087325 | 41403,15 | 5,8926 | 363 | E.R. | ABC G | Cs6g01330 |

| | | | | | | |
|---|---|---|---|---|---|---|
| TCONS_00085441 | 132593,43 | 7,8679 | 1174 | plasma membrane | ABC G | Cs5g13760 |
| TCONS_00085440 | 18175,52 | 6,887 | 159 | vacuole | ABC G | Cs5g13760 |
| TCONS_00084950 | 86749,31 | 6,5015 | 775 | plasma membrane | ABC G | Cs6g01330 |
| TCONS_00084948 | 137934,65 | 6,5931 | 1218 | plasma membrane | ABC G | Cs6g01330 |
| TCONS_00084947 | 141227,66 | 6,6279 | 1247 | plasma membrane | ABC G | Cs6g01330 |
| TCONS_00083379 | 66930,45 | 9,2959 | 604 | plasma membrane | ABC G | orange1.1t02544 |
| TCONS_00082842 | 42303,44 | 9,0279 | 364 | plasma membrane | ABC G | Cs6g02250 |
| TCONS_00082841 | 17329,96 | 9,9551 | 149 | nucleus | ABC G | Cs6g02250 |
| TCONS_00082558 | 93748,86 | 9,3593 | 829 | plasma membrane | ABC G | Cs1g13620 |
| TCONS_00082557 | 58835,62 | 7,0814 | 521 | cytosol | ABC G | Cs1g13620 |
| TCONS_00081251 | 165085,24 | 8,2034 | 1458 | plasma membrane | ABC G | Cs6g02210 |
| TCONS_00080820 | 26350,81 | 9,8679 | 231 | cytosol | ABC G | Cs6g02250 |
| TCONS_00079758 | 46775,38 | 6,8769 | 422 | plasma membrane | ABC G | Cs6g02250 |
| TCONS_00079420 | 29858,47 | 9,0717 | 263 | vacuole | ABC G | Cs6g05320 |
| TCONS_00079419 | 81275,02 | 8,0374 | 737 | plasma membrane | ABC G | Cs6g05280 |
| TCONS_00079418 | 72483,81 | 8,092 | 651 | plasma membrane | ABC G | Cs6g05270 |
| TCONS_00076442 | 149230,7 | 7,7144 | 1326 | plasma membrane | ABC G | Cs1g18920 |
| TCONS_00076441 | 148195,03 | 7,9792 | 1311 | plasma membrane | ABC G | Cs1g18930 |
| TCONS_00076439 | 144892,13 | 7,7566 | 1283 | plasma membrane | ABC G | Cs1g18930 |
| TCONS_00076436 | 30907,98 | 10,1429 | 265 | cytosol | ABC G | ? |
| TCONS_00076220 | 144152,63 | 8,9493 | 1280 | plasma membrane | ABC G | Cs5g13760 |
| TCONS_00074289 | 95598,55 | 7,8067 | 846 | plasma membrane | ABC G | Cs6g01130_a |
| TCONS_00072490 | 124041,34 | 8,8628 | 1111 | plasma membrane | ABC G | Cs7g24870 |
| TCONS_00071185 | 166799,14 | 8 | 1472 | plasma membrane | ABC G | orange1.1t01531 |
| TCONS_00070199 | 66615,51 | 8,7726 | 592 | plasma membrane | ABC G | Cs6g05270 |
| TCONS_00068834 | 13370,37 | 10,9972 | 122 | cytosol | ABC G | orange1.1t02321_a |
| TCONS_00068831 | 37862,85 | 7,9148 | 330 | plasma membrane | ABC G | orange1.1t02321 |
| TCONS_00068828 | 41946,33 | 8,0557 | 376 | mitocondria | ABC G | orange1.1t02321 |

| | | | | | | |
|---|---|---|---|---|---|---|
| TCONS_00068827 | 76953,72 | 8,2362 | 683 | plasma membrane | ABC G | orange1.1t02321 |
| TCONS_00068615 | 68722,2 | 9,2709 | 612 | plasma membrane | ABC G | Cs7g24870_a |
| TCONS_00064966 | 49804,12 | 8,8014 | 449 | golgi | ABC G | orange1.1t00127_a |
| TCONS_00064965 | 25345,04 | 7,3462 | 223 | plasma membrane | ABC G | orange1.1t01996 |
| TCONS_00064050 | 73309,61 | 8,6615 | 648 | plasma membrane | ABC G | Cs1g09530 |
| TCONS_00061978 | 148436,35 | 7,7382 | 1314 | plasma membrane | ABC G | Cs1g15710 |
| TCONS_00054535 | 55518,6 | 9,4559 | 493 | plasma membrane | ABC G | Cs5g17290 |
| TCONS_00054533 | 54966,21 | 8,2126 | 500 | cytosol | ABC G | Cs5g17290 |
| TCONS_00054528 | 79707,16 | 8,6803 | 715 | plasma membrane | ABC G | Cs5g17290 |
| TCONS_00053514 | 67987,7 | 6,2911 | 604 | cytosol | ABC G | Cs1g01500 |
| TCONS_00053513 | 37178,16 | 6,849 | 329 | cytosol | ABC G | Cs1g01500 |
| TCONS_00053089 | 67472,76 | 9,1039 | 605 | plasma membrane | ABC G | Cs9g18660_a |
| TCONS_00050546 | 101744,58 | 9,5249 | 899 | plasma membrane | ABC G | Cs1g13620 |
| TCONS_00050545 | 164163,46 | 8,8657 | 1455 | plasma membrane | ABC G | Cs1g13620 |
| TCONS_00050544 | 160180,46 | 8,1096 | 1425 | plasma membrane | ABC G | Cs1g13640 |
| TCONS_00050541 | 159978,23 | 8,6815 | 1412 | plasma membrane | ABC G | Cs1g13650 |
| TCONS_00050539 | 148567,09 | 8,8585 | 1315 | plasma membrane | ABC G | Cs1g13660 |
| TCONS_00050538 | 162306,23 | 7,3781 | 1436 | plasma membrane | ABC G | Cs1g13680 |
| TCONS_00050536 | 163592,29 | 7,9602 | 1446 | plasma membrane | ABC G | Cs1g13690 |
| TCONS_00050535 | 162134,52 | 8,3761 | 1435 | plasma membrane | ABC G | Cs1g13700 |
| TCONS_00050532 | 164929,9 | 8,1443 | 1459 | plasma membrane | ABC G | Cs1g13710 |
| TCONS_00050530 | 111391,01 | 8,5203 | 993 | plasma membrane | ABC G | Cs1g13720 |
| TCONS_00050519 | 165990,72 | 8,3549 | 1469 | plasma membrane | ABC G | Cs4g09130 |
| TCONS_00050518 | 72409,85 | 7,2202 | 640 | plasma membrane | ABC G | Cs1g13720 |
| TCONS_00050517 | 150420,48 | 8,3174 | 1332 | plasma membrane | ABC G | Cs1g13730 |
| TCONS_00048605 | 76380,14 | 9,2969 | 674 | plasma membrane | ABC G | Cs4g08300 |
| TCONS_00046720 | 123969,03 | 8,822 | 1118 | plasma membrane | ABC G | Cs8g16110 |
| TCONS_00046616 | 80359,31 | 8,9366 | 723 | plasma membrane | ABC G | Cs8g15960 |

| | | | | | | |
|---|---|---|---|---|---|---|
| TCONS_00046613 | 53455,62 | 8,9756 | 487 | plasma membrane | ABC G | Cs8g15960 |
| TCONS_00046612 | 77511 | 9,1194 | 697 | plasma membrane | ABC G | Cs8g15960 |
| TCONS_00046041 | 43833,54 | 8,0435 | 396 | nucleus | ABC G | Cs1g19860 |
| TCONS_00045285 | 164392,97 | 8,8255 | 1458 | plasma membrane | ABC G | Cs6g15300 |
| TCONS_00045282 | 110790,16 | 8,918 | 988 | plasma membrane | ABC G | Cs6g15280 |
| TCONS_00045281 | 159327,4 | 7,339 | 1403 | plasma membrane | ABC G | orange1.1t01531 |
| TCONS_00045278 | 100677,11 | 7,2409 | 889 | plasma membrane | ABC G | Cs6g01330_a |
| TCONS_00045273 | 158528,42 | 7,0977 | 1403 | plasma membrane | ABC G | Cs6g15300 |
| TCONS_00044877 | 77939,37 | 9,6856 | 697 | plasma membrane | ABC G | Cs2g30650 |
| TCONS_00044875 | 73989,92 | 9,3344 | 661 | plasma membrane | ABC G | Cs2g30650_a |
| TCONS_00044636 | 71045,37 | 7,8188 | 627 | plasma membrane | ABC G | Cs5g08870 |
| TCONS_00041816 | 94991,58 | 9,531 | 846 | plasma membrane | ABC G | orange1.1t00127 |
| TCONS_00040706 | 69007,7 | 9,431 | 610 | plasma membrane | ABC G | Cs2g13730_a |
| TCONS_00038660 | 68983,76 | 9,1971 | 622 | chloroplast | ABC G | Cs2g13730 |
| TCONS_00034840 | 159924,72 | 9,1767 | 1427 | plasma membrane | ABC G | Cs8g16470 |
| TCONS_00034839 | 94000,45 | 9,4414 | 836 | plasma membrane | ABC G | Cs8g16470 |
| TCONS_00033842 | 45799,06 | 8,5341 | 406 | plasma membrane | ABC G | Cs7g28610_a |
| TCONS_00033503 | 58962,31 | 9,9116 | 525 | plasma membrane | ABC G | Cs7g28090 |
| TCONS_00029271 | 77528,86 | 8,7514 | 698 | golgi | ABC G | Cs7g12620 |
| TCONS_00028866 | 85084,45 | 9,3389 | 772 | plasma membrane | ABC G | ? |
| TCONS_00028863 | 54444,1 | 8,2338 | 490 | plasma membrane | ABC G | ? |
| TCONS_00027801 | 60334,67 | 7,243 | 531 | plasma membrane | ABC G | Cs1g20770_a |
| TCONS_00026533 | 122293,91 | 7,9901 | 1086 | plasma membrane | ABC G | Cs9g01400 |
| TCONS_00026527 | 45575,32 | 8,0846 | 397 | cytosol | ABC G | Cs9g01400 |
| TCONS_00026526 | 25722,7 | 10,3057 | 224 | cytosol | ABC G | Cs9g01400 |
| TCONS_00026525 | 119657,27 | 7,0831 | 1051 | plasma membrane | ABC G | Cs9g01400 |
| TCONS_00025723 | 70994,53 | 9,0574 | 637 | plasma membrane | ABC G | Cs6g19470 |
| TCONS_00024640 | 140163,24 | 8,5262 | 1250 | plasma membrane | ABC G | Cs8g16470 |

| | | | | | | |
|---|---|---|---|---|---|---|
| TCONS_00022448 | 57413,25 | 8,6982 | 520 | plasma membrane | ABC G | Cs1g06780 |
| TCONS_00022447 | 35552,84 | 8,0065 | 309 | plasma membrane | ABC G | Cs1g06780 |
| TCONS_00022445 | 38000,1 | 8,3426 | 345 | chloroplast | ABC G | Cs1g06780 |
| TCONS_00021082 | 45553,87 | 10,0202 | 413 | chloroplast | ABC G | Cs2g04080_a |
| TCONS_00014556 | 72039,37 | 9,8131 | 646 | plasma membrane | ABC G | Cs1g20770 |
| TCONS_00013641 | 161335,96 | 9,1147 | 1422 | plasma membrane | ABC G | Cs4g17100 |
| TCONS_00013639 | 101491,62 | 9,3168 | 893 | plasma membrane | ABC G | Cs4g17100 |
| TCONS_00012028 | 162300,73 | 7,0452 | 1436 | plasma membrane | ABC G | Cs4g20440 |
| TCONS_00005376 | 27256,27 | 7,9397 | 243 | plasma membrane | ABC G | Cs3g21080 |
| TCONS_00004136 | 75199,05 | 9,0489 | 679 | plasma membrane | ABC G | Cs3g22340 |
| TCONS_00001537 | 44936,87 | 7,1453 | 399 | cytosol | ABC G | Cs5g33740 |
| TCONS_00001499 | 22256,97 | 9,7118 | 198 | nucleus | ABC G | orange1.1t01659 |
| TCONS_00094695 | 16011,68 | 6,7221 | 146 | cytosol | ABC I | Cs4g04420 |
| TCONS_00080497 | 54738 | 7,0896 | 497 | chloroplast | ABC I | Cs7g22890 |
| TCONS_00067965 | 36406,82 | 6,9638 | 332 | chloroplast | ABC I | Cs7g23800 |
| TCONS_00067934 | 45051,76 | 4,3434 | 394 | cytosol | ABC I | Cs7g23530 |
| TCONS_00067050 | 23654,66 | 5,5354 | 199 | chloroplast | ABC I | Cs5g33740_a |
| TCONS_00066524 | 87011,44 | 5,8905 | 791 | plasma membrane | ABC I | Cs9g11110 |
| TCONS_00064800 | 29884,54 | 7,4136 | 273 | chloroplast | ABC I | Cs1g09070 |
| TCONS_00064799 | 19497,76 | 9,619 | 177 | chloroplast | ABC I | Cs1g09070 |
| TCONS_00063142 | 39195,32 | 8,494 | 349 | cytosol | ABC I | Cs6g06520 |
| TCONS_00048155 | 27477,28 | 10,4245 | 245 | chloroplast | ABC I | Cs4g01550 |
| TCONS_00046520 | 32993,69 | 6,3034 | 294 | nucleus | ABC I | Cs5g29600 |
| TCONS_00046519 | 23299,67 | 5,9505 | 201 | cytosol | ABC I | Cs5g29600 |
| TCONS_00043579 | 22787,67 | 10,0709 | 206 | vacuole | ABC I | Cs4g04420_a |
| TCONS_00043434 | 34979,9 | 7,246 | 317 | plasma membrane | ABC I | Cs4g04420 |
| TCONS_00040108 | 33348,38 | 9,8604 | 304 | chloroplast | ABC I | Cs2g25590 |
| TCONS_00040105 | 18404,9 | 10,7889 | 168 | chloroplast | ABC I | Cs2g25590 |

| | | | | | | |
|---|---|---|---|---|---|---|
| TCONS_00034495 | 32847,23 | 10,5325 | 291 | E.R. | ABC I | Cs7g09160_a |
| TCONS_00020726 | 62921,7 | 5,439 | 565 | chloroplast | ABC I | Cs2g07970 |
| TCONS_00001230 | 67732,03 | 4,9734 | 614 | chloroplast | ABC I | orange1.1t01791 |

| Transcript | Molecular Weigh | Isoelectric Point | Sequence Lenght | Sublocalization | Annotation | Subfamily | Putative Gene |
|---|---|---|---|---|---|---|---|
| TCONS_00050554 | 64111.66 | 10.0865 | 584 | plasma membrane | Plastidic ATP/ADP | AATP | Cs1g13530 |
| TCONS_00080566 | 47904.98 | 8.7492 | 442 | plasma membrane | Anion | Anion | Cs6g07670 |
| TCONS_00035116 | 58078.05 | 10.0500 | 533 | chloroplast | Anion | Anion | Cs8g19810 |
| TCONS_00033193 | 53111.68 | 6.6901 | 489 | plasma membrane | Anion | Anion | Cs8g01190 |
| TCONS_00023636 | 58006.20 | 9.4607 | 526 | plasma membrane | Anion | Anion | Cs4g05170 |
| TCONS_00023632 | 80151.32 | 5.1906 | 747 | plasma membrane | Anion | Anion | Cs4g05170 |
| TCONS_00023627 | 76808.37 | 5.3881 | 715 | cytosol | Anion | Anion | Cs4g05170 |
| TCONS_00023625 | 53190.22 | 4.8059 | 495 | vacuole | Anion | Anion | Cs4g05170 |
| TCONS_00000748 | 44855.05 | 9.5612 | 412 | plasma membrane | Ascorbate | ASC | orange1.1t00376 |
| TCONS_00000747 | 69186.39 | 9.2324 | 620 | plasma membrane | Ascorbate | ASC | orange1.1t00376 |
| TCONS_00000745 | 54291.40 | 9.6342 | 490 | plasma membrane | Ascorbate | ASC | orange1.1t00376 |
| TCONS_00084645 | 62349.91 | 10.0206 | 574 | plasma membrane | Folate-Biopterin | FBT | Cs5g35030 |
| TCONS_00069190 | 44647.85 | 9.4466 | 411 | vacuole | Folate-Biopterin | FBT | Cs7g15410 |
| TCONS_00041936 | 55065.03 | 10.2587 | 496 | plasma membrane | Folate-Biopterin | FBT | Cs4g10690 |
| TCONS_00034542 | 56388.42 | 7.7881 | 511 | plasma membrane | Folate-Biopterin | FBT | Cs7g06530 |
| TCONS_00034540 | 54750.77 | 8.7188 | 492 | plasma membrane | Folate-Biopterin | FBT | Cs7g06540 |
| TCONS_00034539 | 53060.91 | 8.6966 | 476 | plasma membrane | Folate-Biopterin | FBT | Cs7g06540 |
| TCONS_00034145 | 54711.45 | 5.2402 | 499 | plasma membrane | Folate-Biopterin | FBT | Cs7g06580 |
| TCONS_00021278 | 40658.16 | 4.9001 | 379 | plasma membrane | Folate-Biopterin | FBT | Cs9g07150 |
| TCONS_00021277 | 61865.69 | 8.2114 | 569 | plasma membrane | Folate-Biopterin | FBT | Cs9g07150 |
| TCONS_00052617 | 57942.40 | 9.2053 | 538 | plasma membrane | Glycerol-3-phosphate | G3Pp | Cs5g07940 |
| TCONS_00048249 | 54218.49 | 7.0189 | 499 | plasma membrane | Glycerol-3-phosphate | G3Pp | Cs4g01150 |

| | | | | | | | |
|---|---|---|---|---|---|---|---|
| TCONS_00037536 | 59172.52 | 9.0540 | 547 | plasma membrane | Glycerol-3-phosphate | G3Pp | Cs9g14660 |
| TCONS_00078018 | 54928.07 | 5.0839 | 511 | plasma membrane | Inositol | INT | orange1.1t03682 |
| TCONS_00078017 | 35324.40 | 5.7519 | 323 | vacuole | Inositol | INT | orange1.1t03682 |
| TCONS_00070943 | 24612.76 | 7.1867 | 227 | plasma membrane | Inositol | INT | orange1.1t03682 |
| TCONS_00070938 | 19476.51 | 5.5685 | 177 | chloroplast | Inositol | INT | Cs7g20530 |
| TCONS_00019980 | 63731.35 | 9.2529 | 586 | plasma membrane | Inositol | INT | Cs2g08110 |
| TCONS_00016856 | 62789.41 | 8.2940 | 581 | plasma membrane | Inositol | INT | Cs2g02060 |
| TCONS_00079647 | 57487.21 | 9.3174 | 530 | plasma membrane | Nitrate | NPF | orange1.1t02415 |
| TCONS_00055955 | 57942.40 | 9.2053 | 538 | plasma membrane | Nitrate | NPF | Cs2g27650 |
| TCONS_00046610 | 53994.25 | 6.4793 | 493 | plasma membrane | Nitrate | NPF | Cs8g16000 |
| TCONS_00093948 | 11507.44 | 4.2497 | 105 | cytosol | Organic Cation/carnitine | OCT | Cs4g04930 |
| TCONS_00093947 | 20449.15 | 5.0770 | 189 | vacuole | Organic Cation/carnitine | OCT | Cs4g04930 |
| TCONS_00093237 | 46214.88 | 9.7904 | 422 | vacuole | Organic Cation/carnitine | OCT | Cs1g18890 |
| TCONS_00091575 | 52470.82 | 8.5248 | 489 | plasma membrane | Organic Cation/carnitine | OCT | Cs4g04960 |
| TCONS_00044238 | 38710.20 | 4.7590 | 352 | plasma membrane | Organic Cation/carnitine | OCT | orange1.1t03244 |
| TCONS_00044237 | 49867.44 | 9.2545 | 457 | plasma membrane | Organic Cation/carnitine | OCT | orange1.1t03245 |
| TCONS_00044236 | 51921.51 | 8.7318 | 473 | plasma membrane | Organic Cation/carnitine | OCT | orange1.1t03244 |
| TCONS_00035355 | 50281.97 | 9.8426 | 460 | plasma membrane | Organic Cation/carnitine | OCT | Cs8g20990 |
| TCONS_00035354 | 78531.72 | 5.0690 | 736 | plasma membrane | Organic Cation/carnitine | OCT | Cs8g20990 |
| TCONS_00035353 | 46687.91 | 9.1259 | 427 | plasma membrane | Organic Cation/carnitine | OCT | Cs8g20990 |
| TCONS_00023595 | 65079.63 | 9.2476 | 584 | vacuole | Organic Cation/carnitine | OCT | Cs4g04960 |
| TCONS_00023588 | 69186.39 | 9.2324 | 620 | plasma membrane | Organic Cation/carnitine | OCT | Cs4g04940 |
| TCONS_00023587 | 46584.12 | 9.3293 | 413 | plasma membrane | Organic Cation/carnitine | OCT | Cs4g04940 |
| TCONS_00023584 | 78745.00 | 6.6601 | 704 | plasma membrane | Organic Cation/carnitine | OCT | Cs4g04930 |
| TCONS_00023577 | 70058.08 | 5.9581 | 619 | vacuole | Organic Cation/carnitine | OCT | Cs4g04920 |
| TCONS_00023567 | 27538.92 | 8.6440 | 255 | vacuole | Organic Cation/carnitine | OCT | Cs4g04910 |
| TCONS_00022996 | 58555.62 | 7.6381 | 539 | plasma membrane | Organic Cation/carnitine | OCT | Cs4g12030 |
| TCONS_00085488 | 25587.50 | 10.4829 | 231 | plasma membrane | D-xylose-proton | Other | Cs5g24870 |

| | | | | | | | |
|---|---|---|---|---|---|---|---|
| TCONS_00085487 | 56188.34 | 8.5209 | 523 | plasma membrane | D-xylose-proton | Other | Cs5g24870 |
| TCONS_00085486 | 27290.24 | 8.9826 | 252 | plasma membrane | D-xylose-proton | Other | Cs5g24870 |
| TCONS_00085485 | 28525.64 | 7.0525 | 259 | endoplasmic reticulum | D-xylose-proton | Other | Cs5g24870 |
| TCONS_00035153 | 58563.74 | 7.8722 | 539 | plasma membrane | Sodium-dependent Phosphate | Other | Cs8g19980 |
| TCONS_00035152 | 29663.34 | 9.4923 | 268 | plasma membrane | Sodium-dependent Phosphate | Other | Cs8g19980 |
| TCONS_00035149 | 56898.38 | 8.7905 | 519 | plasma membrane | Sodium-dependent Phosphate | Other | Cs8g19980 |
| TCONS_00035147 | 28856.39 | 9.1738 | 265 | endoplasmic reticulum | Sodium-dependent Phosphate | Other | Cs8g19980 |
| TCONS_00035005 | 60537.00 | 9.8988 | 559 | chloroplast | D-xylose-proton | Other | Cs8g18970 |
| TCONS_00034998 | 49308.64 | 9.2279 | 458 | plasma membrane | D-xylose-proton | Other | Cs8g18970 |
| TCONS_00080449 | 25266.33 | 8.6770 | 234 | plasma membrane | Plastidic Glucose | pGlcT | Cs4g16110 |
| TCONS_00080446 | 34604.22 | 6.7392 | 316 | chloroplast | Plastidic Glucose | pGlcT | Cs4g16110 |
| TCONS_00080442 | 54588.16 | 8.4681 | 504 | endoplasmic reticulum | Plastidic Glucose | pGlcT | Cs4g16110 |
| TCONS_00080437 | 39948.48 | 8.6022 | 364 | plasma membrane | Plastidic Glucose | pGlcT | Cs4g16110 |
| TCONS_00080434 | 59681.48 | 7.4355 | 547 | plasma membrane | Plastidic Glucose | pGlcT | Cs4g16110 |
| TCONS_00080432 | 57482.92 | 7.3998 | 526 | plasma membrane | Plastidic Glucose | pGlcT | Cs4g16110 |
| TCONS_00057315 | 32360.57 | 4.6924 | 299 | plasma membrane | Plastidic Glucose | pGlcT | Cs2g20710 |
| TCONS_00057312 | 59681.48 | 7.4355 | 547 | plasma membrane | Plastidic Glucose | pGlcT | Cs2g20710 |
| TCONS_00057311 | 60456.87 | 8.5433 | 558 | plasma membrane | Plastidic Glucose | pGlcT | Cs2g20710 |
| TCONS_00057305 | 26365.00 | 8.0920 | 243 | plasma membrane | Plastidic Glucose | pGlcT | Cs2g20710 |
| TCONS_00057304 | 40797.39 | 7.8636 | 372 | plasma membrane | Plastidic Glucose | pGlcT | Cs2g20710 |
| TCONS_00052823 | 67072.51 | 10.1052 | 622 | plasma membrane | Plastidic Glucose | pGlcT | Cs1g15250 |
| TCONS_00052822 | 44326.31 | 9.9976 | 420 | plasma membrane | Plastidic Glucose | pGlcT | Cs1g15250 |
| TCONS_00052820 | 42249.51 | 9.2611 | 401 | plasma membrane | Plastidic Glucose | pGlcT | Cs1g15250 |
| TCONS_00049032 | 67072.51 | 10.1052 | 622 | plasma membrane | Plastidic Glucose | pGlcT | Cs6g18490 |
| TCONS_00049025 | 59172.52 | 9.0540 | 547 | chloroplast | Plastidic Glucose | pGlcT | Cs6g18490 |
| TCONS_00049023 | 56360.41 | 8.9078 | 514 | plasma membrane | Plastidic Glucose | pGlcT | Cs6g18490 |
| TCONS_00063695 | 57461.27 | 7.7683 | 535 | plasma membrane | Polyol | PMT | Cs4g15780 |
| TCONS_00063694 | 58621.50 | 9.2732 | 541 | plasma membrane | Polyol | PMT | Cs4g15770 |

| | | | | | | | |
|---|---|---|---|---|---|---|---|
| TCONS_00032737 | 78531.72 | 5.0690 | 736 | plasma membrane | Monosaccharide-sensing | PMT | Cs8g01220 |
| TCONS_00020275 | 55711.14 | 5.9938 | 519 | plasma membrane | Polyol | PMT | Cs2g06380 |
| TCONS_00015117 | 31335.97 | 10.3404 | 285 | plasma membrane | Polyol | PMT | Cs1g23890 |
| TCONS_00006794 | 80151.32 | 5.1906 | 747 | plasma membrane | Monosaccharide-sensing | PMT | Cs3g27610 |
| TCONS_00006792 | 57576.63 | 5.2352 | 525 | plasma membrane | Monosaccharide-sensing | PMT | Cs3g27610 |
| TCONS_00006791 | 56239.69 | 7.2794 | 509 | vacuole | Monosaccharide-sensing | PMT | Cs3g27610 |
| TCONS_00095514 | 37362.12 | 6.4038 | 338 | plasma membrane | Phosphate | PTH | Cs8g06780 |
| TCONS_00054421 | 59451.34 | 8.1361 | 534 | plasma membrane | Inorganic Phosphate | PTH | Cs9g18560 |
| TCONS_00051661 | 56655.36 | 9.6122 | 520 | plasma membrane | Phosphate | PTH | Cs9g10540 |
| TCONS_00051658 | 57030.88 | 9.0777 | 519 | plasma membrane | Inorganic Phosphate | PTH | Cs9g10530 |
| TCONS_00046572 | 59253.94 | 8.9326 | 542 | plasma membrane | Inorganic Phosphate | PTH | Cs5g29860 |
| TCONS_00033621 | 44188.37 | 8.0427 | 402 | plasma membrane | Inorganic Phosphate | PTH | Cs7g29450 |
| TCONS_00006801 | 59714.57 | 8.7491 | 538 | plasma membrane | Phosphate | PTH | Cs3g27660 |
| TCONS_00040340 | 70058.08 | 5.9581 | 619 | plasma membrane | SPX Domain | PTH5 | Cs5g10180 |
| TCONS_00002435 | 78745.00 | 6.6601 | 704 | plasma membrane | SPX Domain | PTH5 | orange1.1t00286 |
| TCONS_00040338 | 35145.90 | 8.1386 | 324 | plasma membrane | SPX Domain | PTH8 | Cs5g10180 |
| TCONS_00094087 | 17718.65 | 5.6301 | 162 | plasma membrane | Sphingolipid | Spinster | Cs8g03150 |
| TCONS_00080200 | 36297.97 | 6.9506 | 329 | plasma membrane | Spinster | Spinster | Cs1g01440 |
| TCONS_00032801 | 39484.82 | 9.8127 | 358 | plasma membrane | Sphingolipid | Spinster | Cs8g03150 |
| TCONS_00032800 | 31663.76 | 9.2530 | 283 | plasma membrane | Sphingolipid | Spinster | Cs8g03150 |
| TCONS_00013792 | 55268.53 | 6.0920 | 511 | plasma membrane | Sphingolipid | Spinster | Cs1g20960 |
| TCONS_00013791 | 59854.78 | 6.0936 | 555 | plasma membrane | Sphingolipid | Spinster | Cs1g20960 |
| TCONS_00013789 | 43218.56 | 7.8872 | 402 | plasma membrane | Sphingolipid | Spinster | Cs1g20960 |
| TCONS_00091023 | 56188.34 | 8.5209 | 523 | plasma membrane | Sugar | STP | Cs7g13670 |
| TCONS_00089296 | 32378.30 | 4.7276 | 305 | vacuole | ERD6-like | STP | Cs3g12510 |
| TCONS_00089291 | 24114.36 | 6.2618 | 224 | vacuole | ERD6-like | STP | Cs3g12510 |
| TCONS_00089290 | 15851.97 | 9.4787 | 141 | vacuole | ERD6-like | STP | Cs3g12510 |
| TCONS_00089289 | 15826.60 | 10.1332 | 140 | vacuole | ERD6-like | STP | Cs3g12490 |

| | | | | | | | |
|---|---|---|---|---|---|---|---|
| TCONS_00089288 | 14512.28 | 10.3399 | 127 | vacuole | ERD6-like | STP | Cs3g12490 |
| TCONS_00089287 | 17976.93 | 4.1788 | 170 | vacuole | ERD6-like | STP | Cs3g12510 |
| TCONS_00089286 | 10553.12 | 3.6463 | 106 | extracellular space | ERD6-like | STP | Cs3g12500 |
| TCONS_00087922 | 39182.79 | 4.9863 | 364 | vacuole | Sugar | STP | orange1.1t03853 |
| TCONS_00079572 | 37886.64 | 8.5662 | 343 | plasma membrane | ERD6-like | STP | Cs3g12480 |
| TCONS_00079571 | 35426.84 | 8.7952 | 323 | plasma membrane | ERD6-like | STP | Cs3g12410 |
| TCONS_00079564 | 33506.96 | 4.6451 | 312 | plasma membrane | ERD6-like | STP | Cs3g12420 |
| TCONS_00075160 | 35354.20 | 5.1028 | 335 | vacuole | ERD6-like | STP | Cs3g12540 |
| TCONS_00075157 | 51904.03 | 6.4758 | 488 | vacuole | ERD6-like | STP | Cs3g12540 |
| TCONS_00075156 | 26943.60 | 7.1075 | 249 | chloroplast | ERD6-like | STP | Cs3g12540 |
| TCONS_00075142 | 9660.60 | 7.1045 | 87 | cytosol | ERD6-like | STP | Cs3g12510 |
| TCONS_00075135 | 21854.92 | 9.5502 | 198 | vacuole | ERD6-like | STP | Cs3g12510 |
| TCONS_00075131 | 21252.06 | 8.6742 | 193 | vacuole | ERD6-like | STP | Cs3g12510 |
| TCONS_00075130 | 24761.96 | 6.6831 | 226 | plasma membrane | ERD6-like | STP | Cs3g12520 |
| TCONS_00075127 | 40277.71 | 9.2776 | 366 | plasma membrane | ERD6-like | STP | Cs3g12520 |
| TCONS_00075119 | 19638.09 | 9.3633 | 174 | plasma membrane | ERD6-like | STP | Cs3g12510 |
| TCONS_00075118 | 38016.95 | 8.8851 | 345 | plasma membrane | ERD6-like | STP | Cs3g12520 |
| TCONS_00068298 | 34159.84 | 9.2197 | 308 | plasma membrane | ERD6-like | STP | Cs9g05220 |
| TCONS_00035448 | 56360.41 | 8.9078 | 514 | plasma membrane | Sugar | STP | orange1.1t03833 |
| TCONS_00035137 | 49901.46 | 6.8871 | 458 | plasma membrane | Sugar | STP | Cs8g19880 |
| TCONS_00022196 | 38754.00 | 9.5455 | 355 | plasma membrane | ERD6-like | STP | Cs9g05220 |
| TCONS_00021958 | 47871.04 | 10.4165 | 435 | plasma membrane | Sugar | STP | Cs9g06590 |
| TCONS_00021957 | 57487.21 | 9.3174 | 530 | plasma membrane | Sugar | STP | Cs9g06600 |
| TCONS_00021368 | 58006.20 | 9.4607 | 526 | plasma membrane | Sugar | STP | Cs9g06620 |
| TCONS_00015182 | 37001.28 | 8.2215 | 339 | plasma membrane | ERD6-like | STP | Cs1g24180 |
| TCONS_00015175 | 53131.24 | 8.4353 | 490 | plasma membrane | ERD6-like | STP | Cs1g24180 |
| TCONS_00015174 | 26326.06 | 9.4997 | 242 | plasma membrane | ERD6-like | STP | Cs1g24180 |
| TCONS_00006219 | 55197.80 | 9.0178 | 506 | plasma membrane | ERD6-like | STP | Cs3g24900 |

| | | | | | | | |
|---|---|---|---|---|---|---|---|
| TCONS_00006214 | 25287.62 | 9.0791 | 230 | plasma membrane | ERD6-like | STP | Cs3g24900 |
| TCONS_00006212 | 34157.52 | 8.2489 | 316 | plasma membrane | ERD6-like | STP | Cs3g24900 |
| TCONS_00006209 | 34835.72 | 6.3963 | 321 | plasma membrane | ERD6-like | STP | Cs3g24900 |
| TCONS_00001908 | 14411.12 | 7.0042 | 133 | vacuole | ERD6-like | STP | Cs5g32070 |
| TCONS_00001907 | 54498.57 | 7.7054 | 506 | plasma membrane | ERD6-like | STP | Cs5g32060 |

**Table S4.** MATE, ABC, and MFS expressed genes by *Citrus* spp., including gene duplication status, number of putative isoforms produced, and the annotation.

| Gene ID | Duplication Status | Isoforms | Subfamily |
|---|---|---|---|
| Cs2g13500_m | Dispersed | 1 | MATE I |
| Cs2g31280 | Dispersed | 1 | MATE I |
| Cs3g16650 | Dispersed | 1 | MATE I |
| Cs5g06100 | Dispersed | 1 | MATE I |
| Cs5g19630 | Dispersed | 1 | MATE I |
| Cs5g20110_m | Dispersed | 1 | MATE I |
| Cs6g18150 | WGD | 1 | MATE I |
| Cs2g13500 | WGD | 2 | MATE I |
| orange1t00013 | Singleton | 2 | MATE I |
| Cs6g18160 | Tandem | 1 | MATE I |
| Cs1g07540 | Tandem | 2 | MATE I |
| Cs2g13530 | Tandem | 2 | MATE I |
| Cs6g18200 | Tandem | 3 | MATE I |
| Cs1g07550 | Tandem | 4 | MATE I |
| Cs2g13510 | Tandem | 4 | MATE I |
| Cs5g21940 | Dispersed | 1 | MATE II |
| Cs3g17670 | WGD | 1 | MATE II |
| Cs1g20120 | WGD | 3 | MATE II |
| Cs3g17660 | WGD | 3 | MATE II |
| Cs1g20130 | Tandem | 2 | MATE II |
| Cs3g24330 | Dispersed | 3 | MATE II |
| Cs5g28190 | Dispersed | 3 | MATE II |
| orange1t00390 | Singleton | 1 | MATE II |
| Cs9g19420 | Tandem | 1 | MATE II |
| Cs9g19430 | Tandem | 1 | MATE II |
| Cs7g09190 | Tandem | 3 | MATE II |
| Cs2g15610 | Dispersed | 1 | MATE III |
| Cs2g08000 | Dispersed | 2 | MATE III |
| Cs2g03200 | WGD | 1 | MATE III |
| Cs2g21460 | WGD | 1 | MATE III |
| Cs2g03220 | Tandem | 1 | MATE III |
| Cs2g15120 | Tandem | 1 | MATE III |
| Cs2g15080 | Tandem | 2 | MATE III |
| Cs6g09270 | Dispersed | 2 | MATE IV |
| Cs2g30270 | WGD | 2 | MATE IV |
| Cs8g01660 | WGD | 2 | MATE IV |
| Cs1g26350 | WGD | 3 | MATE IV |
| orange1t03403 | Singleton | 2 | MATE IV |
| Cs8g08480 | Tandem | 1 | MATE IV |
| Cs8g08470 | Tandem | 3 | MATE IV |
| Cs3g22600 | Dispersed | 1 | MATE V |

| | | | |
|---|---|---|---|
| Cs4g15415_m | Dispersed | 1 | MATE V |
| Cs5g09710 | Dispersed | 1 | MATE V |
| Cs9g04120 | Dispersed | 1 | MATE V |
| Cs5g10270 | WGD | 1 | MATE V |
| Cs7g16290_m | WGD | 1 | MATE V |
| Cs9g16870 | WGD | 1 | MATE V |
| Cs6g09880 | WGD | 2 | MATE V |
| Cs5g04110 | Tandem | 4 | ABC A |
| Cs5g04120 | Tandem | 2 | ABC A |
| orange1t02318 | Singleton | 2 | ABC A |
| Cs1g08880 | Dispersed | 2 | ABC B |
| Cs1g16850 | Dispersed | 1 | ABC B |
| Cs2g04070 | Tandem | 1 | ABC B |
| Cs2g04080 | Tandem | 3 | ABC B |
| Cs2g04080_a | WGD | 1 | ABC B |
| Cs2g27440 | WGD | 4 | ABC B |
| Cs3g09820 | Tandem | 1 | ABC B |
| Cs3g26380 | Singleton | 3 | ABC B |
| Cs4g11230 | Dispersed | 1 | ABC B |
| Cs4g15420 | Tandem | 1 | ABC B |
| Cs5g12150 | Dispersed | 2 | ABC B |
| Cs6g06520 | Dispersed | 1 | ABC B |
| Cs6g14480 | Singleton | 3 | ABC B |
| Cs6g19410 | Singleton | 2 | ABC B |
| Cs6g20270 | Tandem | 2 | ABC B |
| Cs6g20280 | Tandem | 1 | ABC B |
| Cs6g20290 | Tandem | 1 | ABC B |
| Cs6g21110 | Dispersed | 1 | ABC B |
| Cs7g09160 | WGD | 2 | ABC B |
| Cs7g09730 | Dispersed | 1 | ABC B |
| Cs7g28610 | Dispersed | 3 | ABC B |
| Cs9g01170 | Tandem | 1 | ABC B |
| Cs9g01170_a | Tandem | 1 | ABC B |
| Cs9g11040 | Dispersed | 1 | ABC B |
| Cs9g16780 | Dispersed | 1 | ABC B |
| orange1t00937 | Singleton | 1 | ABC B |
| orange1t01769_a | Dispersed | 1 | ABC B |
| Cs1g02710 | Dispersed | 1 | ABC C |
| Cs1g13700 | Tandem | 2 | ABC C |
| Cs1g13720 | Tandem | 1 | ABC C |
| Cs1g18450 | Tandem | 4 | ABC C |
| Cs1g18460 | Tandem | 1 | ABC C |
| Cs2g09550 | Tandem | 2 | ABC C |

| | | | |
|---|---|---|---|
| Cs2g09560 | Tandem | 1 | ABC C |
| Cs4g09120 | Tandem | 1 | ABC C |
| Cs4g09130 | Tandem | 3 | ABC C |
| Cs4g09140 | Tandem | 3 | ABC C |
| Cs4g09150 | Tandem | 2 | ABC C |
| Cs5g02710 | WGD | 2 | ABC C |
| Cs5g28990 | Dispersed | 1 | ABC C |
| Cs5g33060 | Dispersed | 3 | ABC C |
| Cs7g10200 | Dispersed | 5 | ABC C |
| Cs7g32530 | Dispersed | 1 | ABC C |
| Cs9g18660 | Tandem | 3 | ABC C |
| orange1t02765 | Singleton | 6 | ABC C |
| Cs1g01510 | Dispersed | 3 | ABC D |
| orange1t03223 | Singleton | 5 | ABC D |
| Cs1g13980 | Dispersed | 2 | ABC F |
| Cs5g01810 | Dispersed | 2 | ABC F |
| Cs8g15910 | Dispersed | 1 | ABC F |
| Cs9g09560 | Dispersed | 1 | ABC F |
| orange1t04404 | Singleton | 1 | ABC F |
| Cs1g09530 | Dispersed | 1 | ABC G |
| Cs1g15710 | Dispersed | 1 | ABC G |
| Cs1g20770 | Dispersed | 1 | ABC G |
| Cs1g20770_a | Dispersed | 1 | ABC G |
| Cs2g13730 | Dispersed | 1 | ABC G |
| Cs2g13730_a | Dispersed | 1 | ABC G |
| Cs3g21080 | Dispersed | 1 | ABC G |
| Cs3g22340 | Dispersed | 1 | ABC G |
| Cs4g08300 | Dispersed | 1 | ABC G |
| Cs4g20440 | Dispersed | 1 | ABC G |
| Cs5g08870 | Dispersed | 1 | ABC G |
| Cs5g33740 | Dispersed | 1 | ABC G |
| Cs1g13620 | Tandem | 4 | ABC G |
| Cs1g13690 | Tandem | 1 | ABC G |
| Cs5g17290 | Dispersed | 3 | ABC G |
| Cs6g05320 | Tandem | 1 | ABC G |
| Cs6g01130_a | Tandem | 1 | ABC G |
| Cs7g28090 | Dispersed | 1 | ABC G |
| Cs1g13720 | Tandem | 3 | ABC G |
| Cs6g01131_a | Tandem | 1 | ABC G |
| orange1t01659 | Singleton | 1 | ABC G |
| Cs1g13680 | Tandem | 1 | ABC G |
| Cs1g01420 | WGD | 1 | ABC G |
| Cs6g15280 | Tandem | 1 | ABC G |

| | | | |
|---|---|---|---|
| Cs6g01330_a | Tandem | 1 | ABC G |
| Cs7g24870 | WGD | 1 | ABC G |
| orange1t01996 | Singleton | 1 | ABC G |
| Cs9g01400 | WGD | 4 | ABC G |
| Cs6g05280 | Tandem | 1 | ABC G |
| Cs1g18930 | Tandem | 2 | ABC G |
| Cs6g05270 | Tandem | 2 | ABC G |
| Cs1g13660 | Tandem | 2 | ABC G |
| Cs7g28610_a | Dispersed | 1 | ABC G |
| orange1t02544 | Singleton | 1 | ABC G |
| Cs2g30650 | Tandem | 1 | ABC G |
| Cs7g24870_a | Dispersed | 1 | ABC G |
| Cs7g12620 | Dispersed | 1 | ABC G |
| Cs8g15960 | Dispersed | 3 | ABC G |
| Cs1g13710 | Tandem | 1 | ABC G |
| Cs1g18920 | Tandem | 1 | ABC G |
| Cs9g18660_a | Dispersed | 1 | ABC G |
| orange1t00127 | Singleton | 1 | ABC G |
| Cs1g06780 | WGD | 3 | ABC G |
| Cs1g13650 | Tandem | 1 | ABC G |
| orange1t01531 | Singleton | 2 | ABC G |
| orange1t01993 | Singleton | 3 | ABC G |
| orange1t00127_a | Dispersed | 1 | ABC G |
| Cs6g02210 | WGD | 1 | ABC G |
| orange1t02321_a | Dispersed | 1 | ABC G |
| Cs1g19860 | Tandem | 1 | ABC G |
| Cs8g16470 | Dispersed | 3 | ABC G |
| Cs1g13730 | Tandem | 1 | ABC G |
| Cs6g02250 | Tandem | 4 | ABC G |
| orange1t02321 | Singleton | 3 | ABC G |
| Cs1g13640 | Tandem | 1 | ABC G |
| Cs1g01500 | Dispersed | 2 | ABC G |
| Cs4g17100 | Dispersed | 2 | ABC G |
| Cs6g15300 | Tandem | 2 | ABC G |
| Cs8g16110 | WGD | 1 | ABC G |
| Cs5g13760 | Dispersed | 3 | ABC G |
| Cs6g19470 | Dispersed | 1 | ABC G |
| Cs2g30650_a | Tandem | 1 | ABC G |
| Cs6g01330 | Tandem | 4 | ABC G |
| Cs9g11110 | Dispersed | 1 | ABC I |
| Cs4g01550 | Dispersed | 1 | ABC I |
| Cs1g09070 | Dispersed | 2 | ABC I |
| Cs2g25590 | Dispersed | 2 | ABC I |

| | | | |
|---|---|---|---|
| Cs4g04420 | Dispersed | 2 | ABC I |
| Cs5g29600 | Dispersed | 2 | ABC I |
| Cs4g16980 | WGD | 1 | ABC I |
| Cs7g23530 | WGD | 1 | ABC I |
| Cs7g23800 | Singleton | 1 | ABC I |
| orange1t01791 | Singleton | 1 | ABC I |
| Cs5g33740_a | Tandem | 1 | ABC I |
| Cs4g04420_a | Dispersed | 1 | ABC I |
| Cs7g09160_a | Tandem | 1 | ABC I |
| Cs2g07970 | Dispersed | 1 | ABC I |
| Cs7g22890 | Dispersed | 1 | ABC I |
| Cs1g13530 | Singleton | 1 | AATP |
| Cs4g05170 | Dispersed | 4 | Anion |
| Cs6g07670 | WGD | 1 | Anion |
| Cs8g01190 | Dispersed | 1 | Anion |
| Cs8g19810 | WGD | 1 | Anion |
| orange1.1t00376 | Singleton | 3 | ASC |
| Cs4g10690 | Dispersed | 1 | FBT |
| Cs5g35030 | Dispersed | 1 | FBT |
| Cs7g06530 | Tandem | 1 | FBT |
| Cs7g06540 | Tandem | 2 | FBT |
| Cs7g06580 | Tandem | 1 | FBT |
| Cs7g15410 | Dispersed | 1 | FBT |
| Cs9g07150 | Dispersed | 2 | FBT |
| Cs4g01150 | Dispersed | 1 | G3Pp |
| Cs5g07940 | Dispersed | 1 | G3Pp |
| Cs9g14660 | Dispersed | 1 | G3Pp |
| Cs2g02060 | Dispersed | 1 | INT |
| Cs2g08110 | Dispersed | 1 | INT |
| Cs7g20530 | Dispersed | 1 | INT |
| orange1.1t03682 | Singleton | 3 | INT |
| Cs2g27650 | WGD | 1 | NPF |
| Cs8g16000 | Tandem | 1 | NPF |
| orange1.1t02415 | Singleton | 1 | NPF |
| Cs1g18890 | Dispersed | 1 | OCT |
| Cs4g04910 | Tandem | 1 | OCT |
| Cs4g04920 | Tandem | 1 | OCT |
| Cs4g04930 | Tandem | 3 | OCT |
| Cs4g04940 | Tandem | 2 | OCT |
| Cs4g04960 | Tandem | 2 | OCT |
| Cs4g12030 | Dispersed | 1 | OCT |
| Cs8g20990 | Dispersed | 3 | OCT |
| orange1.1t03244 | Singleton | 2 | OCT |
| orange1.1t03245 | Singleton | 1 | OCT |

| | | | |
|---|---|---|---|
| Cs5g24870 | Dispersed | 4 | Other |
| Cs8g18970 | Dispersed | 2 | Other |
| Cs8g19980 | Dispersed | 4 | Other |
| Cs1g15250 | WGD | 3 | pGlcT |
| Cs2g20710 | Dispersed | 5 | pGlcT |
| Cs4g16110 | WGD | 6 | pGlcT |
| Cs6g18490 | Dispersed | 3 | pGlcT |
| Cs1g23890 | Dispersed | 1 | PMT |
| Cs2g06380 | Dispersed | 1 | PMT |
| Cs3g27610 | WGD | 3 | PMT |
| Cs4g15770 | Tandem | 1 | PMT |
| Cs4g15780 | Tandem | 1 | PMT |
| Cs8g01220 | WGD | 1 | PMT |
| Cs3g27660 | Dispersed | 1 | PTH |
| Cs5g29860 | WGD | 1 | PTH |
| Cs7g29450 | Dispersed | 1 | PTH |
| Cs8g06780 | Dispersed | 1 | PTH |
| Cs9g10530 | WGD | 1 | PTH |
| Cs9g10540 | Tandem | 1 | PTH |
| Cs9g18560 | Dispersed | 1 | PTH |
| Cs5g10180 | Singleton | 2 | PTH5 |
| orange1.1t00286 | Singleton | 1 | PTH5 |
| Cs1g01440 | Tandem | 1 | Spinster |
| Cs1g20960 | WGD | 3 | Spinster |
| Cs8g03150 | WGD | 3 | Spinster |
| Cs1g24180 | WGD | 3 | STP |
| Cs3g12410 | WGD | 1 | STP |
| Cs3g12420 | Tandem | 1 | STP |
| Cs3g12480 | Tandem | 1 | STP |
| Cs3g12490 | Tandem | 2 | STP |
| Cs3g12500 | Tandem | 1 | STP |
| Cs3g12510 | Tandem | 8 | STP |
| Cs3g12520 | Tandem | 3 | STP |
| Cs3g12540 | WGD | 3 | STP |
| Cs3g24900 | WGD | 4 | STP |
| Cs5g32060 | Tandem | 1 | STP |
| Cs5g32070 | Tandem | 1 | STP |
| Cs7g13670 | Dispersed | 1 | STP |
| Cs8g19880 | Dispersed | 1 | STP |
| Cs9g05220 | Dispersed | 2 | STP |
| Cs9g06590 | Tandem | 1 | STP |
| Cs9g06600 | Tandem | 1 | STP |
| Cs9g06620 | Tandem | 1 | STP |
| orange1.1t03833 | Singleton | 1 | STP |
| orange1.1t03853 | Singleton | 1 | STP |

**Table S5.** MATE, ABC, and MFS differentially expressed, $Log_2FC$ values according to the time points after bacterial inoculation (HAI) and species, *p*-value, and annotation.

| Transcript ID | $Log_2FC$ | HAI | *p*-value | Subfamily |
|---|---|---|---|---|
| TCONS_00087169 | -3.86 | K72 | 0.03 | MATE I |
| TCONS_00087168 | -3.25 | PR72 | 0.04 | MATE I |
| TCONS_00087168 | -3.75 | K72 | 0.03 | MATE I |
| TCONS_00077204 | -2.88 | K72 | 0.03 | MATE IV |
| TCONS_00077202 | -4.37 | K72 | 0.01 | MATE IV |
| TCONS_00077200 | -2.93 | K72 | 0.03 | MATE IV |
| TCONS_00074259 | 5.68 | K72 | 0.02 | MATE IV |
| TCONS_00074258 | -5.53 | LG48 | 0.03 | MATE IV |
| TCONS_00074258 | 4.41 | K72 | 0.02 | MATE IV |
| TCONS_00073445 | -5.76 | H48 | 0.05 | MATE II |
| TCONS_00062153 | -3.12 | BA72 | 0.05 | MATE I |
| TCONS_00062153 | -3.35 | PK48 | 0.03 | MATE I |
| TCONS_00062153 | -3.70 | PK72 | 0.02 | MATE I |
| TCONS_00062153 | -3.92 | PR48 | 0.02 | MATE I |
| TCONS_00062153 | -3.97 | BA48 | 0.03 | MATE I |
| TCONS_00062153 | -4.86 | PR72 | 0.01 | MATE I |
| TCONS_00062153 | -6.59 | LG48 | 0.01 | MATE I |
| TCONS_00062150 | -3.08 | BA72 | 0.04 | MATE I |
| TCONS_00062150 | -3.59 | PR48 | 0.03 | MATE I |
| TCONS_00062150 | -3.64 | PK72 | 0.02 | MATE I |
| TCONS_00062150 | -4.36 | PR72 | 0.01 | MATE I |
| TCONS_00062150 | -4.37 | PR24 | 0.03 | MATE I |
| TCONS_00062150 | -5.10 | K48 | 0.04 | MATE I |
| TCONS_00062150 | -6.94 | LG48 | 0.00 | MATE I |
| TCONS_00053098 | -3.71 | PR48 | 0.04 | MATE II |
| TCONS_00045960 | -2.72 | K72 | 0.03 | MATE II |
| TCONS_00045960 | -2.94 | BA72 | 0.02 | MATE II |
| TCONS_00045960 | -3.00 | K48 | 0.02 | MATE II |
| TCONS_00045960 | -3.87 | LG48 | 0.00 | MATE II |
| TCONS_00045960 | -3.97 | PR24 | 0.02 | MATE II |
| TCONS_00045960 | -4.05 | K24 | 0.00 | MATE II |
| TCONS_00045957 | -2.66 | PR48 | 0.05 | MATE II |
| TCONS_00045957 | -2.71 | K72 | 0.03 | MATE II |
| TCONS_00045957 | -3.00 | BA72 | 0.02 | MATE II |
| TCONS_00045957 | -3.09 | K48 | 0.02 | MATE II |
| TCONS_00045957 | -3.98 | PR24 | 0.02 | MATE II |
| TCONS_00045957 | -4.00 | LG48 | 0.00 | MATE II |
| TCONS_00045957 | -4.23 | K24 | 0.00 | MATE II |
| TCONS_00042346 | 3.33 | K72 | 0.02 | MATE I |
| TCONS_00042345 | 3.23 | K72 | 0.02 | MATE I |
| TCONS_00041065 | -2.63 | PK72 | 0.05 | MATE I |

| | | | | |
|---|---|---|---|---|
| TCONS_00041065 | -3.01 | K72 | 0.04 | MATE I |
| TCONS_00041065 | -3.04 | PR72 | 0.04 | MATE I |
| TCONS_00040567 | 4.51 | BA48 | 0.04 | MATE I |
| TCONS_00040567 | 5.34 | BA72 | 0.03 | MATE I |
| TCONS_00038869 | 2.71 | K48 | 0.05 | MATE I |
| TCONS_00038869 | 3.61 | BA48 | 0.01 | MATE I |
| TCONS_00038869 | 3.67 | PR48 | 0.03 | MATE I |
| TCONS_00038869 | 5.26 | BA72 | 0.00 | MATE I |
| TCONS_00038869 | 5.33 | PR72 | 0.00 | MATE I |
| TCONS_00038868 | 2.68 | K72 | 0.05 | MATE I |
| TCONS_00038868 | 2.76 | K48 | 0.04 | MATE I |
| TCONS_00038868 | 3.04 | H48 | 0.04 | MATE I |
| TCONS_00038868 | 3.31 | PR48 | 0.04 | MATE I |
| TCONS_00038868 | 3.75 | BA48 | 0.01 | MATE I |
| TCONS_00038868 | 3.94 | PR72 | 0.01 | MATE I |
| TCONS_00038868 | 5.11 | BA72 | 0.00 | MATE I |
| TCONS_00038712 | 2.51 | PK48 | 0.05 | MATE I |
| TCONS_00038712 | 2.58 | H72 | 0.04 | MATE I |
| TCONS_00038709 | 2.54 | PK48 | 0.04 | MATE I |
| TCONS_00038709 | 2.55 | H72 | 0.04 | MATE I |
| TCONS_00038708 | 2.54 | H72 | 0.04 | MATE I |
| TCONS_00038708 | 2.55 | PK48 | 0.04 | MATE I |
| TCONS_00038707 | 2.50 | PK48 | 0.04 | MATE I |
| TCONS_00038707 | 2.51 | H72 | 0.04 | MATE I |
| TCONS_00027519 | -2.80 | S72 | 0.04 | MATE IV |
| TCONS_00027519 | -2.88 | PR48 | 0.04 | MATE IV |
| TCONS_00027519 | -2.93 | PK48 | 0.02 | MATE IV |
| TCONS_00027519 | -4.49 | K72 | 0.01 | MATE IV |
| TCONS_00027519 | -5.86 | PR24 | 0.00 | MATE IV |
| TCONS_00027519 | 3.37 | S48 | 0.02 | MATE IV |
| TCONS_00027518 | -2.84 | S72 | 0.04 | MATE IV |
| TCONS_00027518 | -2.87 | PR48 | 0.04 | MATE IV |
| TCONS_00027518 | -2.91 | PK48 | 0.02 | MATE IV |
| TCONS_00027518 | -4.49 | K72 | 0.01 | MATE IV |
| TCONS_00027518 | -5.91 | PR24 | 0.00 | MATE IV |
| TCONS_00027518 | 3.38 | S48 | 0.02 | MATE IV |
| TCONS_00020012 | -2.98 | LG72 | 0.04 | MATE III |
| TCONS_00020012 | -3.16 | S48 | 0.05 | MATE III |
| TCONS_00020012 | -3.63 | K24 | 0.03 | MATE III |
| TCONS_00020012 | -4.89 | K72 | 0.05 | MATE III |
| TCONS_00020012 | -5.19 | LG48 | 0.00 | MATE III |
| TCONS_00020012 | -5.35 | K48 | 0.02 | MATE III |
| TCONS_00016588 | -3.01 | PK48 | 0.05 | MATE III |

| | | | | |
|---|---|---|---|---|
| TCONS_00016588 | -5.35 | LG48 | 0.04 | MATE III |
| TCONS_00016588 | -7.11 | K48 | 0.00 | MATE III |
| TCONS_00016588 | -7.46 | K24 | 0.00 | MATE III |
| TCONS_00011593 | -2.99 | LG24 | 0.03 | MATE I |
| TCONS_00011593 | 3.08 | PR48 | 0.03 | MATE I |
| TCONS_00011593 | 3.20 | H24 | 0.02 | MATE I |
| TCONS_00011593 | 3.39 | V48 | 0.02 | MATE I |
| TCONS_00011593 | 3.55 | H72 | 0.01 | MATE I |
| TCONS_00011593 | 3.62 | H48 | 0.01 | MATE I |
| TCONS_00011593 | 3.81 | K48 | 0.00 | MATE I |
| TCONS_00011593 | 4.47 | PR72 | 0.00 | MATE I |
| TCONS_00011593 | 4.62 | S48 | 0.00 | MATE I |
| TCONS_00011593 | 5.04 | PK72 | 0.00 | MATE I |
| TCONS_00007405 | 2.59 | LG48 | 0.04 | MATE II |
| TCONS_00007404 | 2.59 | LG48 | 0.04 | MATE II |
| TCONS_00094098 | −2.99 | PR72 | 0.0235 | ABC G |
| TCONS_00094098 | 2.89 | H48 | 0.0197 | ABC G |
| TCONS_00094098 | 3.17 | PR48 | 0.0175 | ABC G |
| TCONS_00094098 | 3.62 | S24 | 0.0112 | ABC G |
| TCONS_00094098 | 4.31 | S48 | 0.0027 | ABC G |
| TCONS_00094097 | −3.04 | PR72 | 0.0212 | ABC G |
| TCONS_00094097 | 2.92 | H48 | 0.0186 | ABC G |
| TCONS_00094097 | 3.26 | PR48 | 0.0144 | ABC G |
| TCONS_00094097 | 3.34 | S24 | 0.0187 | ABC G |
| TCONS_00094097 | 4.34 | S48 | 0.0025 | ABC G |
| TCONS_00094095 | −2.94 | PR72 | 0.0257 | ABC G |
| TCONS_00094095 | 2.91 | H48 | 0.0189 | ABC G |
| TCONS_00094095 | 3.26 | PR48 | 0.0146 | ABC G |
| TCONS_00094095 | 3.42 | S24 | 0.0160 | ABC G |
| TCONS_00094095 | 4.35 | S48 | 0.0024 | ABC G |
| TCONS_00093484 | 3.18 | H24 | 0.0340 | ABC C |
| TCONS_00093484 | 3.33 | H72 | 0.0183 | ABC C |
| TCONS_00093484 | 3.49 | PR72 | 0.0429 | ABC C |
| TCONS_00093484 | 3.59 | H48 | 0.0144 | ABC C |
| TCONS_00093484 | 3.64 | PK48 | 0.0096 | ABC C |
| TCONS_00093484 | 3.83 | S48 | 0.0149 | ABC C |
| TCONS_00093484 | 4.09 | S72 | 0.0082 | ABC C |
| TCONS_00093484 | 4.39 | PR24 | 0.0106 | ABC C |
| TCONS_00093484 | 6.50 | V24 | 0.0176 | ABC C |
| TCONS_00093292 | −4.91 | PR72 | 0.0202 | ABC C |
| TCONS_00093292 | −6.07 | LG48 | 0.0287 | ABC C |
| TCONS_00093292 | 5.23 | K72 | 0.0484 | ABC C |
| TCONS_00093291 | −3.39 | BA72 | 0.0423 | ABC C |
| TCONS_00093291 | −5.23 | PR72 | 0.0085 | ABC C |

| | | | | |
|---|---|---|---|---|
| TCONS_00093291 | −6.67 | LG48 | 0.0095 | ABC C |
| TCONS_00093291 | 6.09 | K72 | 0.0123 | ABC C |
| TCONS_00089868 | −2.98 | BA72 | 0.0499 | ABC C |
| TCONS_00089868 | −4.83 | PR72 | 0.0051 | ABC C |
| TCONS_00089868 | −5.05 | PK72 | 0.0019 | ABC C |
| TCONS_00089868 | −6.07 | LG48 | 0.0116 | ABC C |
| TCONS_00089868 | 5.33 | K72 | 0.0204 | ABC C |
| TCONS_00089867 | −3.40 | BA72 | 0.0280 | ABC C |
| TCONS_00089867 | −4.81 | PR72 | 0.0055 | ABC C |
| TCONS_00089867 | −5.02 | PK72 | 0.0021 | ABC C |
| TCONS_00089867 | −5.96 | LG48 | 0.0142 | ABC C |
| TCONS_00089867 | 5.43 | K72 | 0.0181 | ABC C |
| TCONS_00079419 | 4.48 | K72 | 0.0010 | ABC G |
| TCONS_00076962 | −2.81 | PR72 | 0.0354 | ABC C |
| TCONS_00076962 | −2.88 | K72 | 0.0245 | ABC C |
| TCONS_00076957 | −2.79 | PR72 | 0.0368 | ABC C |
| TCONS_00076957 | −2.87 | K72 | 0.0249 | ABC C |
| TCONS_00076952 | −2.81 | PR72 | 0.0353 | ABC C |
| TCONS_00076952 | −2.90 | K72 | 0.0232 | ABC C |
| TCONS_00076938 | −2.83 | K72 | 0.0264 | ABC C |
| TCONS_00076938 | −2.86 | PR72 | 0.0323 | ABC C |
| TCONS_00076933 | −2.81 | PR72 | 0.0358 | ABC C |
| TCONS_00076933 | −2.87 | K72 | 0.0250 | ABC C |
| TCONS_00076929 | −2.78 | PR72 | 0.0405 | ABC C |
| TCONS_00076929 | −2.84 | K72 | 0.0295 | ABC C |
| TCONS_00073525 | 3.40 | LG72 | 0.0479 | ABC B |
| TCONS_00072490 | 3.11 | K72 | 0.0264 | ABC G |
| TCONS_00072490 | 3.44 | S48 | 0.0246 | ABC G |
| TCONS_00070199 | −4.56 | PR48 | 0.0334 | ABC G |
| TCONS_00068834 | −3.54 | H72 | 0.0154 | ABC G |
| TCONS_00068834 | −4.61 | S48 | 0.0074 | ABC G |
| TCONS_00068834 | −5.00 | K48 | 0.0408 | ABC G |
| TCONS_00068834 | −5.09 | K72 | 0.0378 | ABC G |
| TCONS_00068834 | −5.42 | PR72 | 0.0012 | ABC G |
| TCONS_00068831 | −2.44 | PK24 | 0.0473 | ABC G |
| TCONS_00068831 | −2.52 | LG72 | 0.0449 | ABC G |
| TCONS_00068831 | −2.72 | H72 | 0.0285 | ABC G |
| TCONS_00068831 | −3.33 | K24 | 0.0112 | ABC G |
| TCONS_00068831 | −3.86 | S48 | 0.0056 | ABC G |
| TCONS_00068831 | −4.30 | LG48 | 0.0040 | ABC G |
| TCONS_00068831 | −4.35 | PK72 | 0.0011 | ABC G |
| TCONS_00068831 | −6.04 | K72 | 0.0002 | ABC G |
| TCONS_00068828 | −2.53 | H72 | 0.0414 | ABC G |
| TCONS_00068828 | −2.56 | LG72 | 0.0435 | ABC G |
| TCONS_00068828 | −2.72 | PK24 | 0.0303 | ABC G |

| | | | | |
|---|---|---|---|---|
| TCONS_00068828 | −3.27 | K24 | 0.0143 | ABC G |
| TCONS_00068828 | −3.67 | S48 | 0.0088 | ABC G |
| TCONS_00068828 | −4.51 | LG48 | 0.0040 | ABC G |
| TCONS_00068828 | −4.60 | PK72 | 0.0007 | ABC G |
| TCONS_00068828 | −6.23 | K72 | 0.0002 | ABC G |
| TCONS_00068827 | −2.51 | H72 | 0.0429 | ABC G |
| TCONS_00068827 | −2.56 | LG72 | 0.0432 | ABC G |
| TCONS_00068827 | −2.72 | PK24 | 0.0297 | ABC G |
| TCONS_00068827 | −3.18 | K24 | 0.0166 | ABC G |
| TCONS_00068827 | −3.65 | S48 | 0.0091 | ABC G |
| TCONS_00068827 | −4.53 | LG48 | 0.0038 | ABC G |
| TCONS_00068827 | −4.61 | PK72 | 0.0007 | ABC G |
| TCONS_00068827 | −6.26 | K72 | 0.0002 | ABC G |
| TCONS_00066524 | −2.77 | K72 | 0.0300 | ABC I |
| TCONS_00066524 | −2.92 | K48 | 0.0226 | ABC I |
| TCONS_00064050 | −2.97 | H72 | 0.0396 | ABC G |
| TCONS_00064050 | −3.24 | BA72 | 0.0269 | ABC G |
| TCONS_00064050 | −3.62 | H48 | 0.0173 | ABC G |
| TCONS_00064050 | −3.70 | PK48 | 0.0113 | ABC G |
| TCONS_00064050 | −3.80 | PR48 | 0.0172 | ABC G |
| TCONS_00064050 | −4.05 | PK72 | 0.0067 | ABC G |
| TCONS_00064050 | −5.19 | PR72 | 0.0031 | ABC G |
| TCONS_00064050 | −5.34 | PR24 | 0.0062 | ABC G |
| TCONS_00064050 | −6.68 | K72 | 0.0034 | ABC G |
| TCONS_00064050 | −7.24 | K48 | 0.0012 | ABC G |
| TCONS_00064050 | −7.48 | K24 | 0.0004 | ABC G |
| TCONS_00059566 | −2.66 | K24 | 0.0361 | ABC B |
| TCONS_00059566 | −3.88 | BA72 | 0.0031 | ABC B |
| TCONS_00059566 | −4.10 | PR72 | 0.0033 | ABC B |
| TCONS_00059566 | −4.38 | LG48 | 0.0020 | ABC B |
| TCONS_00059566 | −4.45 | K72 | 0.0016 | ABC B |
| TCONS_00059566 | −5.30 | K48 | 0.0003 | ABC B |
| TCONS_00059564 | −2.69 | K24 | 0.0339 | ABC B |
| TCONS_00059564 | −3.83 | BA72 | 0.0034 | ABC B |
| TCONS_00059564 | −4.10 | PR72 | 0.0033 | ABC B |
| TCONS_00059564 | −4.42 | LG48 | 0.0018 | ABC B |
| TCONS_00059564 | −4.47 | K72 | 0.0016 | ABC B |
| TCONS_00059564 | −5.06 | K48 | 0.0005 | ABC B |
| TCONS_00054535 | −2.55 | K48 | 0.0411 | ABC G |
| TCONS_00054535 | −2.71 | K72 | 0.0315 | ABC G |
| TCONS_00054535 | −4.14 | LG48 | 0.0023 | ABC G |
| TCONS_00054533 | −2.45 | K72 | 0.0494 | ABC G |
| TCONS_00054533 | −2.60 | K48 | 0.0377 | ABC G |
| TCONS_00054533 | −4.12 | LG48 | 0.0024 | ABC G |
| TCONS_00054528 | −2.45 | K72 | 0.0494 | ABC G |

| | | | | |
|---|---|---|---|---|
| TCONS_00054528 | −2.59 | K48 | 0.0379 | ABC G |
| TCONS_00054528 | −4.12 | LG48 | 0.0024 | ABC G |
| TCONS_00054315 | 2.44 | K24 | 0.0452 | ABC C |
| TCONS_00054315 | 2.48 | K48 | 0.0427 | ABC C |
| TCONS_00054311 | 2.39 | K24 | 0.0498 | ABC C |
| TCONS_00054311 | 2.47 | K72 | 0.0439 | ABC C |
| TCONS_00054311 | 2.52 | K48 | 0.0399 | ABC C |
| TCONS_00054310 | 2.41 | K24 | 0.0484 | ABC C |
| TCONS_00054310 | 2.61 | K48 | 0.0349 | ABC C |
| TCONS_00054310 | 2.61 | K72 | 0.0348 | ABC C |
| TCONS_00050544 | −3.60 | BA72 | 0.0047 | ABC G |
| TCONS_00050544 | −3.98 | K48 | 0.0023 | ABC G |
| TCONS_00050544 | −4.21 | PR72 | 0.0021 | ABC G |
| TCONS_00050544 | −4.57 | LG48 | 0.0007 | ABC G |
| TCONS_00050544 | −4.95 | K72 | 0.0003 | ABC G |
| TCONS_00050530 | 2.77 | K24 | 0.0238 | ABC G |
| TCONS_00050519 | 2.76 | K24 | 0.0241 | ABC G |
| TCONS_00050518 | 2.77 | K24 | 0.0238 | ABC G |
| TCONS_00047255 | −2.79 | BA72 | 0.0230 | ABC F |
| TCONS_00047255 | −2.84 | K72 | 0.0218 | ABC F |
| TCONS_00047255 | −3.07 | K48 | 0.0139 | ABC F |
| TCONS_00047255 | −3.46 | PK72 | 0.0063 | ABC F |
| TCONS_00047255 | −4.25 | PR72 | 0.0019 | ABC F |
| TCONS_00045285 | −3.87 | PR72 | 0.0154 | ABC G |
| TCONS_00045282 | −4.07 | PR72 | 0.0107 | ABC G |
| TCONS_00045281 | 3.03 | K48 | 0.0157 | ABC G |
| TCONS_00045281 | 3.11 | PR72 | 0.0182 | ABC G |
| TCONS_00045281 | 3.26 | K24 | 0.0098 | ABC G |
| TCONS_00045281 | 3.30 | PK72 | 0.0090 | ABC G |
| TCONS_00045281 | 3.39 | BA72 | 0.0074 | ABC G |
| TCONS_00045281 | 4.03 | PR24 | 0.0052 | ABC G |
| TCONS_00045278 | 3.12 | PK72 | 0.0125 | ABC G |
| TCONS_00045273 | −2.43 | K72 | 0.0460 | ABC G |
| TCONS_00044877 | −2.59 | K24 | 0.0384 | ABC G |
| TCONS_00044877 | −2.95 | BA72 | 0.0183 | ABC G |
| TCONS_00044877 | −3.40 | PR72 | 0.0118 | ABC G |
| TCONS_00044877 | −4.34 | K72 | 0.0018 | ABC G |
| TCONS_00044877 | −6.18 | LG48 | 0.0001 | ABC G |
| TCONS_00044211 | −2.72 | PR72 | 0.0401 | ABC D |
| TCONS_00044211 | −2.99 | K72 | 0.0199 | ABC D |
| TCONS_00044211 | −3.28 | K48 | 0.0113 | ABC D |
| TCONS_00044210 | −2.60 | PR72 | 0.0490 | ABC D |
| TCONS_00044210 | −2.63 | PK72 | 0.0344 | ABC D |
| TCONS_00044210 | −3.02 | K72 | 0.0186 | ABC D |
| TCONS_00044210 | −3.16 | K48 | 0.0140 | ABC D |

| | | | | |
|---|---|---|---|---|
| TCONS_00044208 | −2.46 | K24 | 0.0484 | ABC D |
| TCONS_00044208 | −2.65 | PR72 | 0.0450 | ABC D |
| TCONS_00044208 | −2.65 | PK72 | 0.0332 | ABC D |
| TCONS_00044208 | −3.15 | K72 | 0.0146 | ABC D |
| TCONS_00044208 | −3.20 | K48 | 0.0128 | ABC D |
| TCONS_00044207 | −2.59 | PR72 | 0.0495 | ABC D |
| TCONS_00044207 | −2.62 | PK72 | 0.0353 | ABC D |
| TCONS_00044207 | −3.02 | K72 | 0.0184 | ABC D |
| TCONS_00044207 | −3.18 | K48 | 0.0135 | ABC D |
| TCONS_00044206 | −2.60 | PK72 | 0.0360 | ABC D |
| TCONS_00044206 | −2.61 | PR72 | 0.0479 | ABC D |
| TCONS_00044206 | −3.09 | K72 | 0.0160 | ABC D |
| TCONS_00044206 | −3.22 | K48 | 0.0121 | ABC D |
| TCONS_00043434 | −3.05 | K72 | 0.0470 | ABC I |
| TCONS_00043255 | 3.22 | PK24 | 0.0262 | ABC C |
| TCONS_00043255 | 3.28 | BA72 | 0.0278 | ABC C |
| TCONS_00043255 | 3.40 | H24 | 0.0318 | ABC C |
| TCONS_00043255 | 3.94 | PK72 | 0.0101 | ABC C |
| TCONS_00043255 | 4.01 | PK48 | 0.0073 | ABC C |
| TCONS_00043255 | 4.21 | S72 | 0.0100 | ABC C |
| TCONS_00043254 | 2.57 | BA24 | 0.0380 | ABC C |
| TCONS_00043254 | 2.62 | H72 | 0.0328 | ABC C |
| TCONS_00043254 | 2.89 | H24 | 0.0309 | ABC C |
| TCONS_00043254 | 2.97 | BA48 | 0.0277 | ABC C |
| TCONS_00043254 | 3.05 | H48 | 0.0147 | ABC C |
| TCONS_00043254 | 3.20 | BA72 | 0.0108 | ABC C |
| TCONS_00043254 | 3.34 | S24 | 0.0194 | ABC C |
| TCONS_00043254 | 3.34 | S48 | 0.0168 | ABC C |
| TCONS_00043254 | 3.95 | PK48 | 0.0023 | ABC C |
| TCONS_00043254 | 4.03 | PK24 | 0.0020 | ABC C |
| TCONS_00043254 | 4.22 | PK72 | 0.0014 | ABC C |
| TCONS_00043254 | 4.42 | S72 | 0.0017 | ABC C |
| TCONS_00043253 | 2.69 | H72 | 0.0296 | ABC C |
| TCONS_00043253 | 2.96 | BA48 | 0.0289 | ABC C |
| TCONS_00043253 | 2.96 | H24 | 0.0278 | ABC C |
| TCONS_00043253 | 3.04 | H48 | 0.0154 | ABC C |
| TCONS_00043253 | 3.44 | S24 | 0.0175 | ABC C |
| TCONS_00043253 | 3.46 | S48 | 0.0141 | ABC C |
| TCONS_00043253 | 3.50 | BA72 | 0.0062 | ABC C |
| TCONS_00043253 | 3.88 | PK72 | 0.0029 | ABC C |
| TCONS_00043253 | 3.98 | PK48 | 0.0023 | ABC C |
| TCONS_00043253 | 4.08 | PK24 | 0.0019 | ABC C |
| TCONS_00043253 | 4.43 | S72 | 0.0018 | ABC C |
| TCONS_00043252 | 2.61 | H72 | 0.0332 | ABC C |
| TCONS_00043252 | 2.67 | BA24 | 0.0321 | ABC C |

| | | | | |
|---|---|---|---|---|
| TCONS_00043252 | 2.89 | H24 | 0.0310 | ABC C |
| TCONS_00043252 | 2.95 | BA48 | 0.0288 | ABC C |
| TCONS_00043252 | 3.03 | H48 | 0.0152 | ABC C |
| TCONS_00043252 | 3.16 | BA72 | 0.0116 | ABC C |
| TCONS_00043252 | 3.32 | S48 | 0.0175 | ABC C |
| TCONS_00043252 | 3.34 | S24 | 0.0196 | ABC C |
| TCONS_00043252 | 3.94 | PK48 | 0.0023 | ABC C |
| TCONS_00043252 | 4.12 | PK24 | 0.0017 | ABC C |
| TCONS_00043252 | 4.22 | PK72 | 0.0014 | ABC C |
| TCONS_00043252 | 4.38 | S72 | 0.0018 | ABC C |
| TCONS_00043251 | 2.48 | BA24 | 0.0452 | ABC C |
| TCONS_00043251 | 2.65 | PR72 | 0.0417 | ABC C |
| TCONS_00043251 | 2.98 | S48 | 0.0311 | ABC C |
| TCONS_00043251 | 3.17 | PK72 | 0.0116 | ABC C |
| TCONS_00043251 | 3.19 | PK24 | 0.0112 | ABC C |
| TCONS_00043251 | 3.30 | PR24 | 0.0214 | ABC C |
| TCONS_00043251 | 3.31 | V48 | 0.0192 | ABC C |
| TCONS_00043251 | 3.55 | V24 | 0.0130 | ABC C |
| TCONS_00043251 | 3.56 | H24 | 0.0091 | ABC C |
| TCONS_00043251 | 3.71 | H48 | 0.0038 | ABC C |
| TCONS_00043251 | 3.80 | BA72 | 0.0032 | ABC C |
| TCONS_00043251 | 4.80 | PK48 | 0.0004 | ABC C |
| TCONS_00043251 | 5.03 | S72 | 0.0005 | ABC C |
| TCONS_00043250 | 2.49 | H72 | 0.0438 | ABC C |
| TCONS_00043250 | 2.61 | PK72 | 0.0360 | ABC C |
| TCONS_00043250 | 2.83 | PK24 | 0.0239 | ABC C |
| TCONS_00043250 | 2.98 | V24 | 0.0424 | ABC C |
| TCONS_00043250 | 3.17 | V48 | 0.0279 | ABC C |
| TCONS_00043250 | 3.17 | PR24 | 0.0304 | ABC C |
| TCONS_00043250 | 3.42 | S48 | 0.0157 | ABC C |
| TCONS_00043250 | 3.62 | H24 | 0.0087 | ABC C |
| TCONS_00043250 | 3.71 | H48 | 0.0044 | ABC C |
| TCONS_00043250 | 3.72 | BA72 | 0.0042 | ABC C |
| TCONS_00043250 | 4.63 | PK48 | 0.0006 | ABC C |
| TCONS_00043250 | 4.96 | S72 | 0.0006 | ABC C |
| TCONS_00043249 | 2.99 | PK24 | 0.0369 | ABC C |
| TCONS_00043249 | 3.51 | PK72 | 0.0341 | ABC C |
| TCONS_00043249 | 3.77 | PK48 | 0.0191 | ABC C |
| TCONS_00043249 | 3.83 | H72 | 0.0248 | ABC C |
| TCONS_00043249 | 4.56 | H48 | 0.0149 | ABC C |
| TCONS_00043249 | 5.04 | S72 | 0.0070 | ABC C |
| TCONS_00041816 | 6.05 | LG48 | 0.0299 | ABC G |
| TCONS_00038660 | −2.42 | BA72 | 0.0476 | ABC G |
| TCONS_00038660 | −3.24 | K72 | 0.0118 | ABC G |
| TCONS_00038660 | −3.29 | K48 | 0.0105 | ABC G |

| | | | | |
|---|---|---|---|---|
| TCONS_00034840 | −2.63 | LG72 | 0.0470 | ABC G |
| TCONS_00034840 | 3.52 | PR72 | 0.0151 | ABC G |
| TCONS_00034840 | 5.53 | BA72 | 0.0002 | ABC G |
| TCONS_00034840 | 5.59 | K72 | 0.0003 | ABC G |
| TCONS_00034839 | −2.65 | LG72 | 0.0458 | ABC G |
| TCONS_00034839 | 3.88 | PR72 | 0.0085 | ABC G |
| TCONS_00034839 | 5.55 | BA72 | 0.0002 | ABC G |
| TCONS_00034839 | 5.71 | K72 | 0.0002 | ABC G |
| TCONS_00029271 | 4.15 | K48 | 0.0059 | ABC G |
| TCONS_00029271 | 5.41 | K72 | 0.0007 | ABC G |
| TCONS_00028866 | −2.77 | BA72 | 0.0263 | ABC G |
| TCONS_00028866 | −2.94 | K72 | 0.0234 | ABC G |
| TCONS_00028866 | −2.97 | K48 | 0.0213 | ABC G |
| TCONS_00028866 | −4.83 | PR72 | 0.0008 | ABC G |
| TCONS_00028866 | −5.27 | K24 | 0.0003 | ABC G |
| TCONS_00028863 | −2.73 | BA72 | 0.0290 | ABC G |
| TCONS_00028863 | −2.93 | K48 | 0.0238 | ABC G |
| TCONS_00028863 | −3.23 | K72 | 0.0146 | ABC G |
| TCONS_00028863 | −4.99 | PR72 | 0.0006 | ABC G |
| TCONS_00028863 | −5.21 | K24 | 0.0003 | ABC G |
| TCONS_00026533 | −3.31 | K48 | 0.0116 | ABC G |
| TCONS_00026527 | −3.38 | K48 | 0.0100 | ABC G |
| TCONS_00026526 | −3.36 | K48 | 0.0104 | ABC G |
| TCONS_00026525 | −3.37 | K48 | 0.0101 | ABC G |
| TCONS_00025857 | −3.53 | PK72 | 0.0114 | ABC B |
| TCONS_00025857 | −4.58 | LG48 | 0.0060 | ABC B |
| TCONS_00025857 | −5.60 | PR72 | 0.0007 | ABC B |
| TCONS_00025857 | −6.25 | K48 | 0.0046 | ABC B |
| TCONS_00025856 | −2.77 | PK72 | 0.0282 | ABC B |
| TCONS_00025856 | −2.99 | LG72 | 0.0206 | ABC B |
| TCONS_00025856 | −4.61 | K24 | 0.0022 | ABC B |
| TCONS_00025856 | −6.12 | K48 | 0.0003 | ABC B |
| TCONS_00025855 | −2.93 | PK72 | 0.0218 | ABC B |
| TCONS_00025855 | −3.05 | LG72 | 0.0193 | ABC B |
| TCONS_00025855 | −4.15 | K24 | 0.0057 | ABC B |
| TCONS_00025855 | −5.48 | K48 | 0.0012 | ABC B |
| TCONS_00025854 | −3.14 | LG72 | 0.0186 | ABC B |
| TCONS_00025854 | −3.20 | PK72 | 0.0152 | ABC B |
| TCONS_00025854 | −4.14 | K24 | 0.0056 | ABC B |
| TCONS_00025854 | −6.45 | LG48 | 0.0002 | ABC B |
| TCONS_00025854 | −7.87 | K48 | 0.0001 | ABC B |
| TCONS_00025723 | −3.96 | K24 | 0.0106 | ABC G |
| TCONS_00025723 | −5.61 | PR72 | 0.0008 | ABC G |
| TCONS_00025723 | −6.78 | K48 | 0.0002 | ABC G |
| TCONS_00025723 | −8.14 | K72 | 0.0002 | ABC G |

| | | | | |
|---|---|---|---|---|
| TCONS_00025446 | 2.58 | PK72 | 0.0423 | ABC B |
| TCONS_00025446 | 3.36 | LG48 | 0.0120 | ABC B |
| TCONS_00022448 | −3.52 | PR72 | 0.0275 | ABC G |
| TCONS_00022448 | 3.72 | H24 | 0.0212 | ABC G |
| TCONS_00022447 | −3.60 | PR72 | 0.0222 | ABC G |
| TCONS_00022447 | 3.85 | H24 | 0.0156 | ABC G |
| TCONS_00022445 | −3.61 | PR72 | 0.0196 | ABC G |
| TCONS_00022445 | 3.82 | H24 | 0.0146 | ABC G |
| TCONS_00020556 | −5.21 | K72 | 0.0002 | ABC C |
| TCONS_00020556 | 3.77 | LG48 | 0.0040 | ABC C |
| TCONS_00020549 | −4.74 | K72 | 0.0007 | ABC C |
| TCONS_00020549 | 3.43 | LG48 | 0.0085 | ABC C |
| TCONS_00019252 | 2.79 | PK72 | 0.0251 | ABC A |
| TCONS_00019252 | 4.22 | LG48 | 0.0015 | ABC A |
| TCONS_00019248 | 2.79 | PK72 | 0.0254 | ABC A |
| TCONS_00019248 | 4.28 | LG48 | 0.0013 | ABC A |
| TCONS_00018550 | 2.75 | S72 | 0.0417 | ABC A |
| TCONS_00018550 | 3.09 | LG48 | 0.0149 | ABC A |
| TCONS_00018548 | 2.80 | S72 | 0.0378 | ABC A |
| TCONS_00018548 | 3.35 | LG48 | 0.0088 | ABC A |
| TCONS_00018546 | 2.81 | S72 | 0.0371 | ABC A |
| TCONS_00018546 | 3.32 | LG48 | 0.0091 | ABC A |
| TCONS_00018545 | 2.80 | S72 | 0.0376 | ABC A |
| TCONS_00018545 | 3.37 | LG48 | 0.0084 | ABC A |
| TCONS_00015584 | −2.70 | PR48 | 0.0482 | ABC B |
| TCONS_00015584 | −2.91 | BA48 | 0.0332 | ABC B |
| TCONS_00015584 | −2.92 | H48 | 0.0223 | ABC B |
| TCONS_00015582 | −2.69 | PR48 | 0.0493 | ABC B |
| TCONS_00015582 | −2.90 | BA48 | 0.0338 | ABC B |
| TCONS_00015582 | −2.91 | H48 | 0.0228 | ABC B |
| TCONS_00013641 | −2.62 | K24 | 0.0368 | ABC G |
| TCONS_00013641 | −2.90 | BA48 | 0.0295 | ABC G |
| TCONS_00013641 | −2.90 | K48 | 0.0223 | ABC G |
| TCONS_00013641 | −3.09 | K72 | 0.0165 | ABC G |
| TCONS_00013641 | −3.13 | PR72 | 0.0193 | ABC G |
| TCONS_00013641 | −3.38 | PK48 | 0.0076 | ABC G |
| TCONS_00013641 | −3.66 | BA72 | 0.0045 | ABC G |
| TCONS_00013641 | −3.84 | H24 | 0.0048 | ABC G |
| TCONS_00013641 | −4.21 | H48 | 0.0015 | ABC G |
| TCONS_00013641 | −4.58 | PR48 | 0.0013 | ABC G |
| TCONS_00013641 | −5.51 | PR24 | 0.0013 | ABC G |
| TCONS_00013639 | −2.57 | K24 | 0.0400 | ABC G |
| TCONS_00013639 | −2.87 | BA48 | 0.0308 | ABC G |
| TCONS_00013639 | −2.87 | K48 | 0.0233 | ABC G |
| TCONS_00013639 | −3.09 | K72 | 0.0162 | ABC G |

| | | | | |
|---|---|---|---|---|
| TCONS_00013639 | −3.14 | PR72 | 0.0190 | ABC G |
| TCONS_00013639 | −3.39 | PK48 | 0.0075 | ABC G |
| TCONS_00013639 | −3.69 | BA72 | 0.0042 | ABC G |
| TCONS_00013639 | −3.86 | H24 | 0.0046 | ABC G |
| TCONS_00013639 | −4.23 | H48 | 0.0014 | ABC G |
| TCONS_00013639 | −4.57 | PR48 | 0.0013 | ABC G |
| TCONS_00013639 | −5.57 | PR24 | 0.0012 | ABC G |
| TCONS_00008590 | 2.88 | H72 | 0.0219 | ABC C |
| TCONS_00008590 | 3.29 | K48 | 0.0139 | ABC C |
| TCONS_00008590 | 3.47 | PR72 | 0.0140 | ABC C |
| TCONS_00008590 | 3.52 | H48 | 0.0064 | ABC C |
| TCONS_00008590 | 3.63 | PK24 | 0.0051 | ABC C |
| TCONS_00008590 | 3.90 | S72 | 0.0054 | ABC C |
| TCONS_00008590 | 4.14 | K72 | 0.0028 | ABC C |
| TCONS_00008590 | 4.86 | K24 | 0.0009 | ABC C |
| TCONS_00008583 | 2.58 | H72 | 0.0353 | ABC C |
| TCONS_00008583 | 2.84 | PR72 | 0.0324 | ABC C |
| TCONS_00008583 | 3.28 | PK24 | 0.0093 | ABC C |
| TCONS_00008583 | 3.38 | K48 | 0.0090 | ABC C |
| TCONS_00008583 | 3.68 | H48 | 0.0041 | ABC C |
| TCONS_00008583 | 3.85 | S72 | 0.0053 | ABC C |
| TCONS_00008583 | 4.47 | K72 | 0.0010 | ABC C |
| TCONS_00008583 | 4.94 | K24 | 0.0004 | ABC C |
| TCONS_00008582 | 2.53 | H72 | 0.0382 | ABC C |
| TCONS_00008582 | 2.82 | PR72 | 0.0333 | ABC C |
| TCONS_00008582 | 3.28 | PK24 | 0.0094 | ABC C |
| TCONS_00008582 | 3.44 | K48 | 0.0081 | ABC C |
| TCONS_00008582 | 3.71 | H48 | 0.0039 | ABC C |
| TCONS_00008582 | 3.87 | S72 | 0.0051 | ABC C |
| TCONS_00008582 | 4.35 | K72 | 0.0013 | ABC C |
| TCONS_00008582 | 4.98 | K24 | 0.0004 | ABC C |
| TCONS_00008581 | 2.62 | H72 | 0.0329 | ABC C |
| TCONS_00008581 | 2.99 | PR72 | 0.0253 | ABC C |
| TCONS_00008581 | 3.29 | PK24 | 0.0091 | ABC C |
| TCONS_00008581 | 3.42 | K48 | 0.0086 | ABC C |
| TCONS_00008581 | 3.72 | H48 | 0.0038 | ABC C |
| TCONS_00008581 | 3.87 | S72 | 0.0051 | ABC C |
| TCONS_00008581 | 4.33 | K72 | 0.0014 | ABC C |
| TCONS_00008581 | 4.93 | K24 | 0.0004 | ABC C |
| TCONS_00008580 | 2.60 | H72 | 0.0339 | ABC C |
| TCONS_00008580 | 2.83 | PR72 | 0.0326 | ABC C |
| TCONS_00008580 | 3.28 | PK24 | 0.0094 | ABC C |
| TCONS_00008580 | 3.41 | K48 | 0.0086 | ABC C |
| TCONS_00008580 | 3.69 | H48 | 0.0041 | ABC C |
| TCONS_00008580 | 3.85 | S72 | 0.0053 | ABC C |

| | | | | |
|---|---|---|---|---|
| TCONS_00008580 | 4.40 | K72 | 0.0012 | ABC C |
| TCONS_00008580 | 5.04 | K24 | 0.0004 | ABC C |
| TCONS_00006540 | −2.77 | BA48 | 0.0367 | ABC B |
| TCONS_00006540 | −2.97 | H48 | 0.0176 | ABC B |
| TCONS_00006540 | −3.16 | K24 | 0.0135 | ABC B |
| TCONS_00006540 | −3.46 | PR48 | 0.0117 | ABC B |
| TCONS_00006540 | −3.89 | H24 | 0.0043 | ABC B |
| TCONS_00006540 | −4.08 | PK48 | 0.0018 | ABC B |
| TCONS_00006540 | −4.12 | BA72 | 0.0017 | ABC B |
| TCONS_00006540 | −4.21 | PR72 | 0.0024 | ABC B |
| TCONS_00006540 | −4.26 | PR24 | 0.0121 | ABC B |
| TCONS_00006540 | −4.30 | K72 | 0.0017 | ABC B |
| TCONS_00006540 | −4.40 | LG48 | 0.0022 | ABC B |
| TCONS_00006540 | −4.82 | K48 | 0.0006 | ABC B |
| TCONS_00006539 | −2.78 | BA48 | 0.0363 | ABC B |
| TCONS_00006539 | −2.99 | H48 | 0.0171 | ABC B |
| TCONS_00006539 | −3.13 | K24 | 0.0144 | ABC B |
| TCONS_00006539 | −3.47 | PR48 | 0.0114 | ABC B |
| TCONS_00006539 | −3.88 | H24 | 0.0044 | ABC B |
| TCONS_00006539 | −4.09 | PK48 | 0.0018 | ABC B |
| TCONS_00006539 | −4.12 | BA72 | 0.0017 | ABC B |
| TCONS_00006539 | −4.20 | PR72 | 0.0025 | ABC B |
| TCONS_00006539 | −4.26 | PR24 | 0.0121 | ABC B |
| TCONS_00006539 | −4.31 | K72 | 0.0017 | ABC B |
| TCONS_00006539 | −4.40 | LG48 | 0.0022 | ABC B |
| TCONS_00006539 | −4.84 | K48 | 0.0006 | ABC B |
| TCONS_00006535 | −2.76 | BA48 | 0.0380 | ABC B |
| TCONS_00006535 | −2.96 | H48 | 0.0182 | ABC B |
| TCONS_00006535 | −3.11 | K24 | 0.0149 | ABC B |
| TCONS_00006535 | −3.47 | PR48 | 0.0115 | ABC B |
| TCONS_00006535 | −3.93 | H24 | 0.0040 | ABC B |
| TCONS_00006535 | −4.09 | PK48 | 0.0018 | ABC B |
| TCONS_00006535 | −4.09 | BA72 | 0.0018 | ABC B |
| TCONS_00006535 | −4.16 | PR72 | 0.0027 | ABC B |
| TCONS_00006535 | −4.22 | K72 | 0.0020 | ABC B |
| TCONS_00006535 | −4.25 | PR24 | 0.0123 | ABC B |
| TCONS_00006535 | −4.70 | K48 | 0.0008 | ABC B |
| TCONS_00006535 | −5.02 | LG48 | 0.0009 | ABC B |
| TCONS_00003170 | 2.68 | H48 | 0.0320 | ABC B |
| TCONS_00003170 | 2.72 | PR48 | 0.0419 | ABC B |
| TCONS_00003170 | 2.80 | S72 | 0.0378 | ABC B |
| TCONS_00003170 | 2.88 | LG48 | 0.0211 | ABC B |
| TCONS_00003170 | 2.92 | V48 | 0.0438 | ABC B |
| TCONS_00003170 | 2.95 | H24 | 0.0288 | ABC B |
| TCONS_00003170 | 3.17 | H72 | 0.0122 | ABC B |

| | | | | |
|---|---|---|---|---|
| TCONS_00003170 | 3.23 | PK72 | 0.0110 | ABC B |
| TCONS_00003170 | 3.39 | BA48 | 0.0138 | ABC B |
| TCONS_00003170 | 3.51 | K72 | 0.0067 | ABC B |
| TCONS_00003170 | 3.63 | PK48 | 0.0048 | ABC B |
| TCONS_00003170 | 3.82 | K24 | 0.0034 | ABC B |
| TCONS_00003170 | 3.93 | PR72 | 0.0040 | ABC B |
| TCONS_00003170 | 4.58 | K48 | 0.0007 | ABC B |
| TCONS_00003170 | 5.20 | BA72 | 0.0002 | ABC B |
| TCONS_00001949 | −3.74 | K72 | 0.0068 | ABC C |
| TCONS_00001949 | 2.52 | PK72 | 0.0461 | ABC C |
| TCONS_00001537 | −3.86 | BA72 | 0.0409 | ABC G |
| TCONS_00001537 | −4.30 | PR48 | 0.0317 | ABC G |
| TCONS_00001537 | −4.60 | H24 | 0.0324 | ABC G |
| TCONS_00001537 | −4.72 | PK48 | 0.0056 | ABC G |
| TCONS_00001537 | −5.50 | PR72 | 0.0129 | ABC G |
| TCONS_00093948 | 3.98 | LG48 | 0,02 | OCT |
| TCONS_00093947 | 4.25 | LG48 | 0,01 | OCT |
| TCONS_00091575 | -2.72 | LG48 | 0,04 | OCT |
| TCONS_00055955 | -4.89 | LG48 | 0,01 | NPF |
| TCONS_00055955 | -5.94 | PK48 | 0,00 | NPF |
| TCONS_00055955 | -3.91 | PR48 | 0,01 | NPF |
| TCONS_00044238 | -6.58 | K48 | 0,00 | OCT |
| TCONS_00044238 | -4.28 | PR72 | 0,01 | OCT |
| TCONS_00035448 | 3.72 | BA48 | 0,01 | STP |
| TCONS_00035448 | 3.83 | BA72 | 0,00 | STP |
| TCONS_00035448 | 2.56 | H48 | 0,04 | STP |
| TCONS_00035448 | 2.51 | H72 | 0,04 | STP |
| TCONS_00035448 | 4.50 | K48 | 0,00 | STP |
| TCONS_00035448 | 4.44 | K72 | 0,00 | STP |
| TCONS_00035448 | 3.74 | PK48 | 0,00 | STP |
| TCONS_00035448 | 2.84 | PK72 | 0,02 | STP |
| TCONS_00035448 | 2.65 | PR48 | 0,04 | STP |
| TCONS_00035448 | 3.99 | PR72 | 0,00 | STP |
| TCONS_00035153 | -2.95 | K48 | 0,02 | Other |
| TCONS_00035153 | -2.87 | K72 | 0,03 | Other |
| TCONS_00035152 | -2.95 | K48 | 0,02 | Other |
| TCONS_00035152 | -3.02 | K72 | 0,02 | Other |
| TCONS_00035149 | -2.93 | K48 | 0,02 | Other |
| TCONS_00035149 | -2.87 | K72 | 0,03 | Other |
| TCONS_00035147 | -2.91 | K48 | 0,03 | Other |
| TCONS_00035147 | -2.91 | K72 | 0,03 | Other |
| TCONS_00034998 | -2.78 | K48 | 0,03 | D-xylose-proton |
| TCONS_00023636 | -3.52 | K72 | 0,01 | Anion |
| TCONS_00023632 | -3.81 | K72 | 0,01 | Anion |

| | | | | |
|---|---|---|---|---|
| TCONS_00023627 | -3.75 | K72 | 0,01 | Anion |
| TCONS_00023625 | -3.87 | K72 | 0,01 | Anion |
| TCONS_00023595 | -2.77 | LG48 | 0,04 | OCT |
| TCONS_00023584 | 3.46 | LG48 | 0,02 | OCT |
| TCONS_00023577 | 3.28 | K72 | 0,01 | OCT |
| TCONS_00022996 | 3.45 | K48 | 0,02 | OCT |
| TCONS_00022996 | 3.70 | K72 | 0,01 | OCT |
| TCONS_00022996 | 2.98 | PR72 | 0,05 | OCT |
| TCONS_00021368 | 2.60 | K72 | 0,03 | STP |
| TCONS_00021368 | 3.01 | S72 | 0,02 | STP |
| TCONS_00002435 | 4.09 | LG24 | 0,03 | PTH5 |
| TCONS_00002435 | -3.63 | PR72 | 0,05 | PTH5 |
| TCONS_00000748 | -2.83 | PR48 | 0,04 | ASC |
| TCONS_00000748 | -3.67 | PR72 | 0,01 | ASC |
| TCONS_00000747 | -2.47 | BA72 | 0,05 | ASC |
| TCONS_00000747 | -2.87 | PR48 | 0,04 | ASC |
| TCONS_00000747 | -3.55 | PR72 | 0,01 | ASC |
| TCONS_00000745 | -2.45 | BA72 | 0,05 | ASC |
| TCONS_00000745 | -2.87 | PR48 | 0,04 | ASC |
| TCONS_00000745 | -3.45 | PR72 | 0,01 | ASC |
